# Steric Hindrance and Secondary Interactions Govern Reconfiguration Between Two Complex Cu^I^ Coordination Cages

**DOI:** 10.1002/anie.4287066

**Published:** 2026-04-14

**Authors:** Houyang Xu, Sudhakar Gaikwad, Tanya K. Ronson, Jonathan R. Nitschke

**Affiliations:** ^1^ Yusuf Hamied Department of Chemistry University of Cambridge Cambridge UK

**Keywords:** metal–organic cages, molecular capsules, secondary interactions, self‐assembly, supramolecular chemistry

## Abstract

A tetramine subcomponent featuring 1,5‐naphthylene arms was designed to exhibit extensive secondary interactions when assembled around stereochemically flexible tetracoordinate Cu^I^ ions. Using 6‐methyl‐2‐formylpyridine, a [Cu^I^_12_L_6_]^12+^ pseudo‐hexagonal prismatic cage formed, stabilized by 29–32 C─H···π interactions and 12 arene stacking interactions. Replacing the aldehyde component with 3‐methyl‐2‐formylpyridine introduced steric clashes that partially prevented these interactions, leading instead to the formation of a [Cu^I^_8_L_4_]^8+^ rectangular open prism. The system exhibited structural interconversion: adding 6‐methyl‐2‐formylpyridine to the [Cu^I^_8_L_4_]^8+^ cage transformed it into the [Cu^I^_12_L_6_]^12+^ structure through selective displacement of 8 out of 24 aldehyde residues per cage, matching the number of sterically‐hindered positions predicted from structural analysis. This work demonstrates rational control over cage architecture through fine‐tuning of steric factors and intermolecular interactions, providing design principles for generating diverse structures from identical building blocks.

## Graphical abstract

Fine‐tuning steric and C─H···π contacts directs assembly of two Cu^I^ cages. An identical naphthylene‐based subcomponent forms a [Cu^I^_12_L_6_]^12+^ pseudo‐hexagonal prism stabilized by 29–32 C─H···π contacts and 12 arene stacking pairs with 6‐methyl‐2‐formylpyridine, whereas the 3‐methyl analog imparts steric clashes, yielding a [Cu^I^_8_L_4_]^8+^ open prism. Subcomponent exchange converts the open prism into the hexagonal cage, demonstrating predictive control.

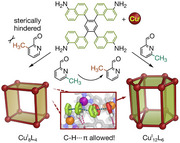

Molecular self‐assembly is the process whereby molecular building blocks are brought together to create larger, more complex supramolecular entities [1, 2, 3]. Steric factors can guide these building blocks to placements within these entities that minimize the overall free energy during assembly [1, 3, 4, 5].

Metal–organic cages are discrete polyhedral three‐dimensional structures, whose cavities can host a diverse range of guests [6, 7, 8, 9]. These cages are held together by coordination bonds between metal ions and multitopic ligands. The reversibility of metal coordination enables conversion between multiple distinct structures [10, 11, 12, 13], using stimuli that include concentration [14, 15, 16], temperature [17], and chemical stimuli such as metal ions [18, 19] or guest binding [20, 21, 22, 23, 24]. These factors reconfigure the metal‐ligand linkages to generate more thermodynamically favorable structures [25].

Secondary interactions and steric factors are important in directing the assembly and reconfigurations of supramolecular architectures. Non‐covalent forces such as aromatic stacking [26, 27, 28, 29], C─H···π contacts [30, 31, 32, 33, 34], solvent effects [35, 36, 37] and hydrogen bonding [38, 39, 40, 41, 42, 43, 44, 45] often act in concert to guide the formation of self‐assembled structures. Although individually weaker than coordination bonds, when acting cooperatively, these interactions can direct structural outcomes and stabilize specific architectures. Harnessing the synergy of these interactions to direct the formation of or impart responsiveness into supramolecular assemblies has thus been an area of active enquiry [12, 25, 40, 46]. Hao Li et al. demonstrated that multiple intramolecular C─H···π interactions can bias self‐assembly toward larger structures that might otherwise be entropically disfavoured, highlighting how accumulated weak interactions can reshape thermodynamic preferences [34]. Stefankiewicz et al. reported a multistate system where a single building block adopts five topologically distinct structures triggered by solvent, temperature, and guest molecules, demonstrating how accumulated weak interactions can be modulated by environmental changes [46]. Szumna et al. showed that hydrogen‐bonded capsules undergo solvent‐dependent structural transformations, with anion‐sealed assemblies forming in weakly polar solvents while dissociating in chloroform, demonstrating how accumulated weak interactions can be modulated by environmental changes [41, 42].

Steric factors often affect the outcome of molecular self‐assembly by directing the formation of favored structures and ruling out unfavorable structures [17, 47, 48, 49, 50, 51, 52]. Dan Li et al. showed that a 2‐methyl substituent on imidazolyl ligands directs formation of tetartoidal Co_20_ cages through pentagonal window formation, while its absence favors cubic structures [53]. Recent work by Liu et al. showed the use of steric hindrance to direct the assembly of large two‐component organic cages, where introducing bulky anthracene and phenazine substituents shifts equilibrium from small tubular structures toward larger capsules by thermodynamically disfavoring close packing [54]. Clever et al. employed steric constraints and hydrogen bonding interactions near coordination sites to direct the formation of low‐symmetry bowl‐shaped assemblies with selective guest binding capabilities [55, 56]. Chand et al. demonstrated that combining ligand rigidity with steric bulk at metal coordination sites enables selective formation of discrete Pd_6_L_6_ trigonal prisms, while more flexible analogues yield dynamic mixtures of products [50]. We have used sterics in combination with geometrical restraints and secondary interactions to generate more complex cage architectures as well [57, 58].

Here we report two Cu^I^ metal–organic cages, which correspond to unreported structure types. These cages interconvert through fine‐tuning of secondary interactions and steric bulk. They incorporate a tetramine subcomponent bearing 1,5‐naphthylene arms, which impose steric constraints and enable C─H···π interactions and arene stacking interactions to take place. One structure is a [Cu^I^_12_L_6_]^12+^ pseudo‐hexagonal prism, generated with assistance from a combination of these interactions. By blocking some with steric bulk, a distinct [Cu^I^_8_L_4_]^8+^ rectangular open prismatic structure forms instead, which can be transformed into the former structure by adding subcomponents whose incorporation re‐enables these interactions.

Steric requirements within tetramine subcomponent **A**, featuring four 1,5‐naphthylene arms joined at a crowded 1,2,4,5‐tetrasubstituted phenyl core, forcing the ligand arms to be perpendicular to potential cage faces. Following the two‐step synthesis of **A** (Scheme S1 and Figures S1–S6), we attempted the self‐assembly of **A** with Cu^I^ and 6‐methyl‐2‐formylpyridine (**B**)—a 2‐formylpyridine derivative known to stabilize Cu^I^ metal centers [59, 60] and sterically decrease vertex curvature. Heating a mixture of **A** (6 equiv.), **B** (24 equiv.) and Cu^I^(MeCN)_4_X (X^−^ = BF_4_^−^ or OTf^−^) (12 equiv.) at 343 K in acetonitrile yielded product **1**, as shown in Figure 1a.

**FIGURE 1:**
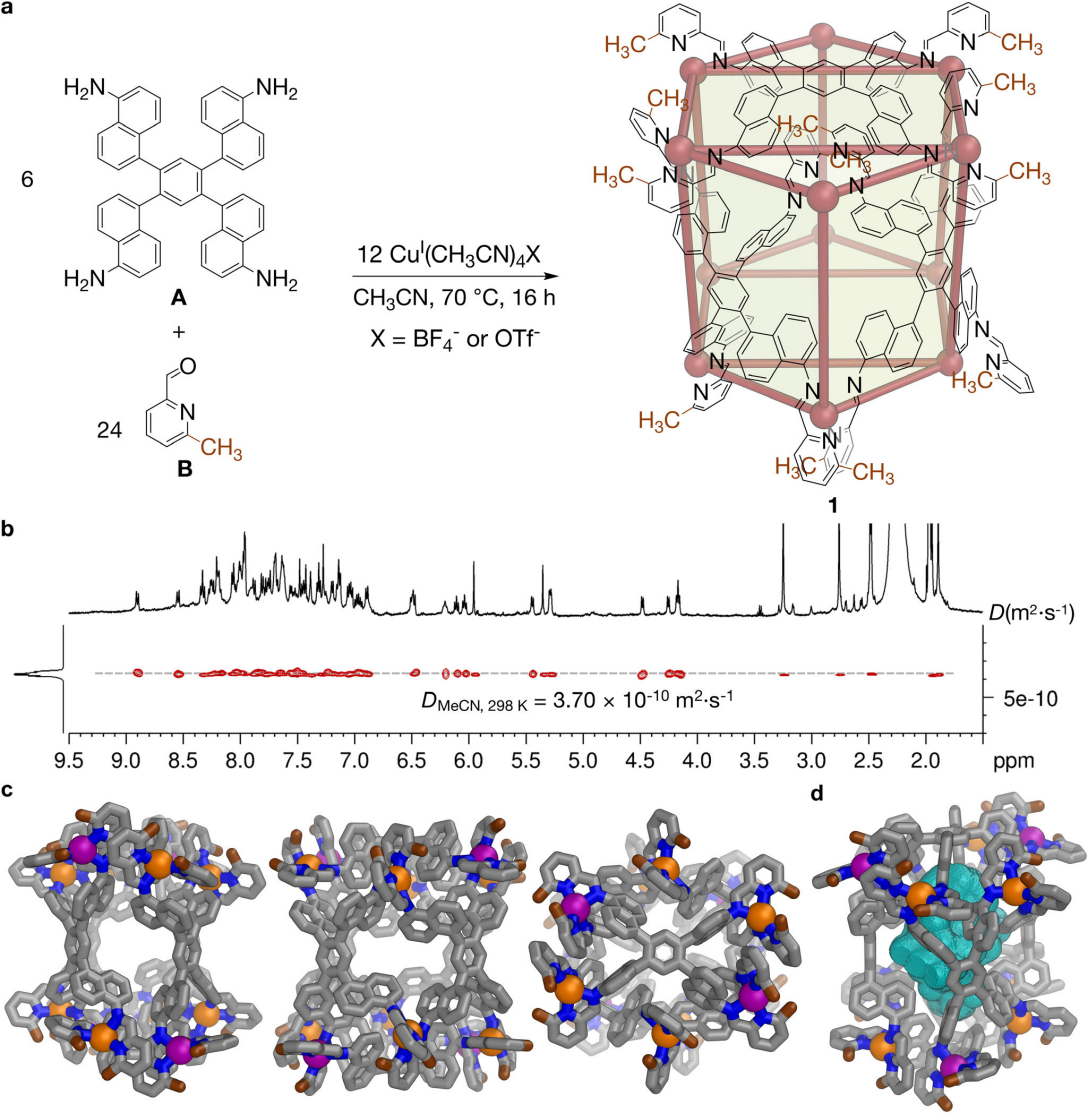
(a) The self‐assembly of Cu^I^_12_L_6_ pseudo‐hexagonal prism **1** from subcomponents **A** and **B** with Cu^I^. (b) ^1^H DOSY spectrum (400 MHz, 298 K, CD_3_CN) of cage **1**, revealing a diffusion coefficient (*D*) of 3.70 × 10^−10^ m^2^·s^−1^, corresponding to a solvodynamic radius of 34.6 Å for all signals attributed to **1**. (c) Views down the three orthogonal *C*_2_‐axes of the single‐crystal X‐ray structure of **1**·(BF_4_)_12_ [61]. (d) Oblique view of the structure, showing its internal cavity shown in teal mesh. Color codes: C = gray or brown (methyl groups on **B** residues), N = blue, Δ‐Cu = purple, Λ‐Cu = orange. Hydrogen atoms, counterions and solvent molecules have been omitted for clarity.

The tetrafluoroborate salt of cage **1** was characterized using nuclear magnetic resonance (NMR) techniques (Figures S7–15). As shown in Figure S13a,b, six unique methyl and imine signals were unambiguously identified in the ^1^H‐^13^C heteronuclear single‐quantum correlation (HSQC) spectrum. Two of the imine signals were unusually shielded (5.93 and 5.32 ppm), consistent with C─H···π interactions. This pattern suggests the presence of three unique vertex environments in **1**, as each vertex provides two distinct ligand arm environments.

As shown in Figure 1b, ^1^H diffusion ordered spectroscopy (^1^H DOSY) results confirmed that all non‐solvent ^1^H NMR signals in the sample showed the same diffusion constant of 3.70 × 10^−10^ m^2^ s^−1^, corresponding to a solvodynamic diameter of 34.6 Å, as calculated using the Stokes‐Einstein equation.

Electrospray ionization mass spectrometry (ESI‐MS) measurements revealed a single set of signals (Figure S16), corresponding to a [Cu^I^_12_L_6_]^12+^ composition, as further confirmed by Electrospray ionization high‐resolution mass spectrometry (ESI‐HRMS) in Figure S17.

Single‐crystal X‐ray diffraction (SCXRD) characterization further elucidated the structure of cage **1** [61]. Figure 1c provides three views of the crystal structure of **1**·(BF_4_)_12_. As the Cu^I^ vertices of **1**·(BF_4_)_12_ describe a hexagonal prism despite the structure being composed of tetratopic ligands, we refer to **1**·(BF_4_)_12_ as a pseudo‐hexagonal prism. Three orthogonal *C*_2_ axes within the structure of **1**·(BF_4_)_12_ indicate that the cage possesses *D*_2_ point group symmetry. The crystal structure of **1**·(BF_4_)_12_ also allowed for assignment of its ^1^H NMR signals with the spatial arrangement of protons revealed, with consistency in these data suggesting the presence of identical structure in the solution and the solid state.

The two enantiomers of **1**·(BF_4_)_12_ are related by inversion in the crystal. Three distinct vertex environments are present within the crystal structure of **1**·(BF_4_)_12_. On each pseudo‐hexagonal face, two Cu^I^ centers adopt Δ‐handedness, while the remaining four display Λ‐handedness, giving a ratio of Δ:Λ = 1:2 for the 12 metal vertices of each cage. Since each vertex links two iminopyridine chelating units, there are six distinct ligand arm environments overall, which matches the number of NMR signals observed in Figure S13a,b. Figure 1d visualizes the Molovol‐calculated [62] cavity, with a prolate shape and a volume of 568 Å^3^.

The trifluoromethanesulfonate salt of **1** was also characterized by ^1^H NMR (Figure S18), ^1^H DOSY (Figure S19) and ESI‐MS (Figure S20). Despite broader signals than **1**·(BF_4_)_12_, ^1^H DOSY (Figure S19) suggested the presence of a single species with a solvodynamic diameter of 35.2 Å. As with **1**·(BF_4_)_12_, a single species of [Cu^I^_12_L_6_]^12+^ composition was observed by ESI‐MS (Figure S20). The crystal structure of **1**·(OTf)_12_ was found to be isostructural to **1**·(BF_4_)_12_, as shown in Figure S33 and Section 3 of the Supporting Information [63]. The ^1^H NMR spectrum of **1**·(OTf)_12_ showed broadened signals (Figure S18), precluding detailed NMR characterization. Complete characterization in solution using NMR was thus undertaken on **1**·(BF_4_)_12_.

A dense network of secondary interactions is inferred to stabilize the structure of **1**. As illustrated in Figure 2a and summarized in Tables S4 and S5, each pseudo‐hexagonal face of **1**·(BF_4_)_12_ features eight aromatic panels undergoing stacking (interplanar distances 3.29–3.92 Å, C_g_···C_g_ distances 3.77‐4.13 Å) and multiple C─H···π interactions. Each end of the cage has four iminopyridine moieties close to adjacent naphthylene panels. Eight C─H···aromatic ring centroid distances were observed from 2.70 to 3.22 Å, falling within the 3.5–3.6 Å [64, 65] limit of weak hydrogen bonds. Figure 2b shows a side view of the cage, which reveals a further eight C─H···π interactions per side of the cage, with C─H···centroid distances ranging from 2.69 to 2.93 Å. Overall, 32 C─H···π interactions and 12 aromatic stacking can be identified in the structure of **1**·(BF_4_)_12_, which we infer to provide strong synergistic stabilization of its structure. Similarly, as shown in Tables S6 and S7; Figure S34, a dense network of 29 C─H···π contacts and 12 aromatic stacks is observed in the crystal structure of **1**·(OTf)_12_, further manifesting the strong tendency for secondary interactions to take place in the formation of **1**’s framework. To further demonstrate the importance of these interactions in the formation of **1**, self‐assembly using 6‐fluoro‐2‐formylpyridine (**D**) in place of **B** under analogous conditions was tested, where the C─H···π contacts may be perturbed by the electron withdrawing effects of fluorine. No discrete cage architecture analogous to **1** was observed to form, as shown by ^1^H NMR and ESI‐HRMS (Figures S43 and S44).

**FIGURE 2:**
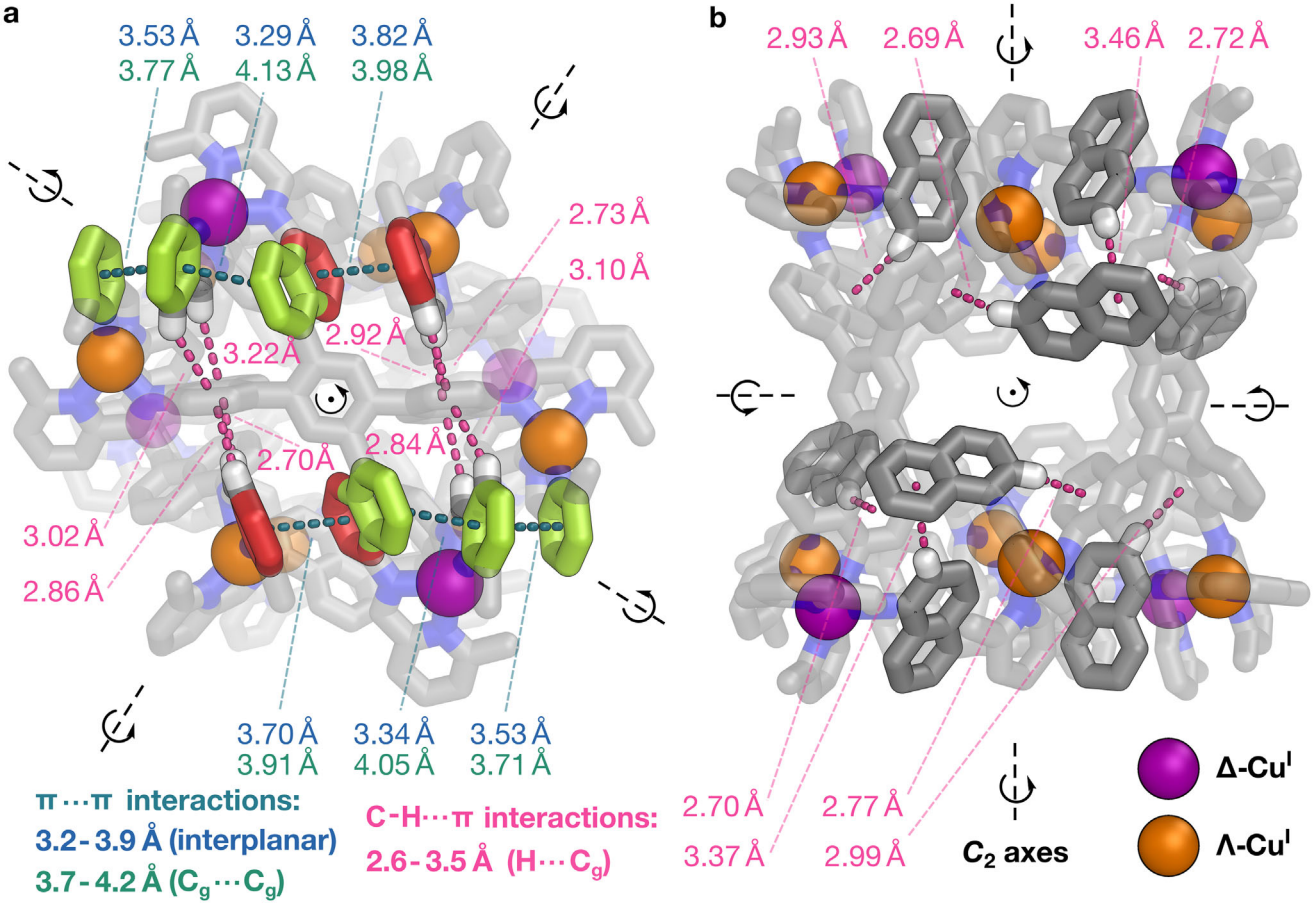
(a) Top and (b) side view of the crystal structure of **1**·(BF_4_)_12_, showing its *C*_2_ axes, and with π···π and C–H···π interactions highlighted in teal and pink, respectively. For π···π interactions, interplanar distances are highlighted in blue and centroid‐centroid (C_g_···C_g_) distances in green; for C–H···π interactions, the hydrogen‐centroid distances are highlighted in pink. Color codes: C = lime (one group of stacking aromatic rings), red (another pair of stacking aromatic rings) or gray, H = white, N = blue, Δ‐Cu = purple, Λ‐Cu = orange. Hydrogen atoms not involved in C–H···π interactions, counterions and solvent molecules have been omitted for clarity.

Given the apparent stabilization of **1** by dense secondary interactions, we investigated the role of these interactions in structure formation. Hydrogens at the 3‐positions of eight pyridyl rings from 6‐methyl‐2‐formylpyridine (**B**) residues participate in C─H···π interactions per cage, with C─H···π (aromatic ring centroid) distances of either 3.02 or 2.92 Å. We thus hypothesized that introducing bulky substituents at these positions would prevent the formation of **1** due to steric clashes and disruption of these stabilizing interactions.

Although the methyl substituents of the **B** residues were originally included to sterically stabilize Cu^I^ vertices within large structures [66], these methyl groups also provide a means to gauge the interplay between steric bulk, secondary interactions and self‐assembly outcomes. We thus generated a counterfactual structure (Figure S35b), to compare with the actual structure in Figure S35a, with the 6‐methyl groups of the pyridyl residues hypothetically moved to the 3‐positions, preserving all other atomic coordinates [67, 68]. This hypothetical structure reveals steric hindrance between the repositioned methyl groups and adjacent naphthylene moieties, with C···C distances between these methyl groups and the nearest naphthylene carbons measuring 2.04‐2.49 Å. Considering the van der Waals radii of methyl groups (∼2.0 Å [69]) and carbon atoms (∼1.77 Å [70]), these distances indicate severe steric clashes that would destabilize the framework of **1**. Therefore, attempting self‐assembly with 3‐methyl‐2‐formylpyridine **C** instead of 6‐methyl‐2‐formylpyridine **B** was undertaken to test whether C─H···π interactions are indeed essential for the stability of **1** by selectively disrupting these interactions through the introduction of steric interference.

As shown in Figure 3a, the self‐assembly of subcomponent **A** (4 equiv.), Cu^I^(MeCN)_4_OTf (8 equiv.) and 3‐methyl‐2‐formylpyridine (**C**, 16 equiv.) at 343 K in acetonitrile generated product **2**, which was characterized by NMR (Figures S21–S29) and ESI‐MS (Figures S30 and S31); its ^1^H DOSY spectrum is shown in Figure 3b. Under the same conditions, all non‐solvent proton signals exhibited the diffusion coefficient of 3.83 × 10^−10^ m^2^ s^−1^, corresponding to a solvodynamic diameter of 33.4 Å. This estimated size of **2** is smaller than **1** (34.6 Å). ESI‐MS measurements showed a single set of signals (Figures S30 and S31), corresponding to a [Cu^I^_8_L_4_]^8+^ composition, consistent with the smaller size indicated by DOSY. The self‐assembly of **A**, **C** and Cu^I^(MeCN)_4_BF_4_, however, was not found by NMR or ESI‐MS to generate a discrete product.

**FIGURE 3:**
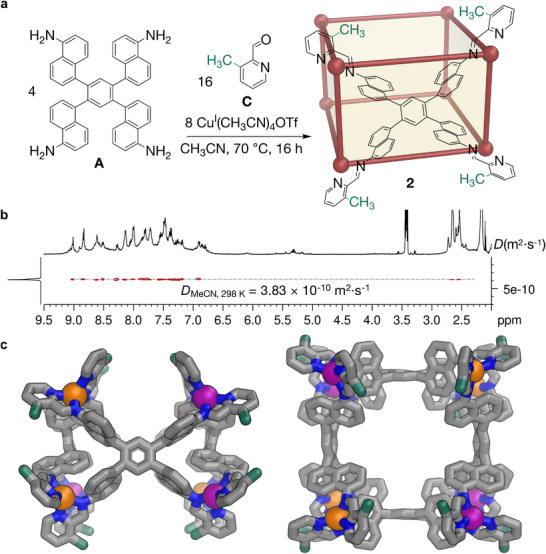
(a) Subcomponent self‐assembly of rectangular open prism **2** from tetramine subcomponent **A**, 3‐methyl‐2‐formylpyridine **C** and Cu^I^(MeCN)_4_OTf in acetonitrile. (b) ^1^H DOSY spectrum (400 MHz, 298 K, CD_3_CN) of cage **2**, revealing a diffusion coefficient (*D*) of 3.83 × 10^−10^ m^2^·s^−1^, which corresponds to a solvodynamic diameter of 33.4 Å. (c) The crystal structure of **2**, showing views down two *C*_2_ axes [71]. The methyl groups from the 3‐methyl‐2‐formylpyridine (C) residues in the structure are highlighted in green, illustrating that they all are positioned in sterically unhindered locations. Color codes: C = gray or green (methyl groups from **C** residues), N = blue, Δ‐Cu = purple, Λ‐Cu = orange. Hydrogen atoms, counterions and solvent molecules have been omitted for clarity.

The solid‐state structure of **2** was determined by SCXRD measurement [71]. As shown in Figure 3c, **2** adopted a rectangular open prismatic geometry. Four ligands occupy the sides of this structure, with the rectangular top and bottom faces uncapped. It possesses three *C*_2_ symmetry axes, each perpendicular to the other two, and three mirror planes, thus exhibiting *D*_2h_ point group symmetry. Each pair of cage vertices along a side edge of the prismatic structure share the same handedness, while the two adjacent vertices on each open quadrilateral face have opposite stereochemical configurations.

The methyl groups from the 3‐methyl‐2‐formylpyridine (**C**) residues are highlighted in green in Figures 3c and S36a. All the 3‐methyl groups of the imine‐pyridyl ligands are oriented towards void areas, out of contact with other moieties of the structures. We generated an alternative hypothetical model [67, 68] based on this structure, where the **C**‐residues are replaced by **B**‐residues, with methyls on the pyridine 6‐positions. As shown in Figure S36b, no significant steric hindrance was observed in either case, indicating that the framework of **2** is free of steric clash for either aldehyde subcomponent. Notably, no clear secondary interactions were found in the SCXRD structure of **2**. We infer that the adoption of this framework for **2** minimizes the steric hindrance incurred by the 3‐methyl groups of **C**, whose presence we hypothesize to destabilize a structure analogous to **1** (Figure S35b). The system may thus form **1** over **2**, as **1** can be further stabilized by dense secondary interactions. The system thus only expresses **2** when the framework of **1** is not feasible due to disruption by unfavorable secondary interactions.

As noted above, the presence and absence of secondary interactions in the structures of **1** and **2**, respectively, raises questions about the relative stabilities of these two structures. Under the assumption that the intermolecular interactions in **1** stabilize its structure significantly, we hypothesized that adding 6‐methyl‐2‐formylpyridine **B** to a sample of **2** might result in its reconfiguration into **1**. However, **1** is also expected to be entropically less favored than the smaller [Cu^I^_8_L_4_]^8+^ structure of **2**, due to both the greater number of components required for assembly and the formation of an enclosed cavity within **1** that restricts solvent mobility.

This structural conversion was monitored by ^1^H NMR and ESI‐MS, with the procedure summarized in Section 5 of the supporting information. To a solution of **2**·(OTf)_8_ in acetonitrile was added 36 equiv. of 6‐methyl‐2‐formylpyridine **B**, and the mixture heated to 343 K for 64 h. This process was tracked by ESI‐MS (Figure S37). The ESI‐MS signals representing the starting [Cu^I^_8_L_4_]^8+^ structure **2** completely disappeared after 64 h (Figure S37d). Instead, another set of signals appeared, corresponding to the [Cu^I^_12_L_6_]^12+^ formula of **1**. This observation suggests complete conversion of **2** to **1**, whereby by addition of **B** resulted in the displacement of the 3‐methyl‐2‐formylpyridine **C** residues within **2**. An intermediate species was identified from a set of signals that corresponded to a [Cu_10_L_5_]^10+^ composition, visible in mass spectra after 16 and 40 h (Figure S37b,c) but which had disappeared after 64 h. The presence of this [Cu_10_L_5_]^10+^ species, inferred to be intermediate in size between **1** and **2**, suggests that the structural reconfiguration may not consist of a full decomposition‐reassembly process.

Figure S38 shows the ^1^H NMR spectrum of a sample of **2**·(OTf)_8_ before and after addition of **B**, also supporting a structural reconfiguration pathway. As shown in Figure S38b, all signals from **2**·(OTf)_8_ had disappeared, and a new set of signals were observed instead. This product, referred to as **1’**, remained intact following workup (Figure S39); ^1^H DOSY NMR (Figure S40) indicates that peaks assigned to **1’** correspond to a structure with a single size. Comparison with the NMR spectrum of **1**·(OTf)_12_ (Figure S38c) suggests a strong resemblance between **1’** and **1**·(OTf)_12_, yet the two spectra do not match completely.

This observation is consistent with our hypothesis because only ∼8 out of 24 aldehyde residues per **1** are in sterically constrained positions (Figure S35b), these residues alone underwent substitution. Given the identical molecular weights of 6‐methyl‐2‐formylpyridine **B** and 3‐methyl‐2‐formylpyridine **C**, the complete reconfiguration observed by ESI‐MS (Figure S37), and the similar but not identical NMR spectra of the new transformed structure and **1**·(OTf)_12_ (Figure S38), it can be inferred that the structural reconfiguration occurred without displacement of all the **C** residues in **2**. As shown in Figure S39, the aldehyde region of the ^1^H NMR spectrum of a sample following reconfiguration showed a 0.30:1.00 ratio of displaced free **C** to remaining **B**. We thus calculated that only ∼8.3 **C** residues were actually replaced by **B** per [Cu^I^_12_L_6_]^12+^ cage (formed from 1.5 [Cu^I^_8_L_4_]^8+^ cages), with the remaining ∼15.7 being **C** residues. Taken together with the observation that no further change in product distribution occurred prior to workup, these results indicate that the final distribution effectively reflects the thermodynamically favored outcome under the conditions employed, although a contribution from kinetic factors cannot be definitively excluded. Additional mass spectrometric evidence was obtained from an analogous experiment using 3‐bromo‐2‐formylpyridine (**E**) instead of **C** for more precise mass spectrometric monitoring. Although this brominated system proved more complex and underwent further exchange after producing the framework of **1**, no species with fewer than eight exchanged residues were observed (Figures S45–S52).

This result lends further strength to the hypothesis that exchange between 6‐methyl‐2‐formylpyridine **B** and 3‐methyl‐2‐formylpyridine **C** residues is crucial for the system to select either the [Cu^I^_12_L_6_]^12+^ or [Cu^I^_8_L_4_]^8+^ framework. The former is favored by its dense network of secondary interactions, which do not occur in the latter structure, wherein **C** residues introduce steric hindrance that prevents the framework of **1** from forming. As shown in Figure 4, the experimental results presented above support the hypothesis that 8 out of 24 aldehyde residues per [Cu^I^_12_L_6_]^12+^ cage would incur severe steric hindrance if **C** were incorporated exclusively. Thus, replacement of these 8 **C** residues with **B** relieves the steric hindrance at these sites, thereby allowing formation of the pseudo‐hexagonal prismatic structure, with the ∼8 **C** residues replaced by **B** per [Cu^I^_12_L_6_]^12+^ cage matching closely the expected amount predicted from structural analysis in Figure S35. Thus, our results indicate that this structural reconfiguration is enabled by rational fine‐tuning of steric factors and intermolecular interactions within metal–organic cage frameworks. This strategy of positioning steric bulk at rationally‐identified spots to push the system towards other structures, and thus controlling dynamic reconfiguration by fine‐tuning the balance between secondary interactions and steric hindrance, may be generalized and applied in metal–organic cage systems featuring more diverse ligands and metal vertices. Similar design logic may also be applicable to other adaptive self‐assembled systems that rely on reversible metal coordination or dynamic covalent chemistry, where subcomponent exchange or post‐assembly modification might tune favorable interactions and steric effects to bias the system toward specific structural frameworks, thereby redirecting the preferred assembly outcome.

**FIGURE 4:**
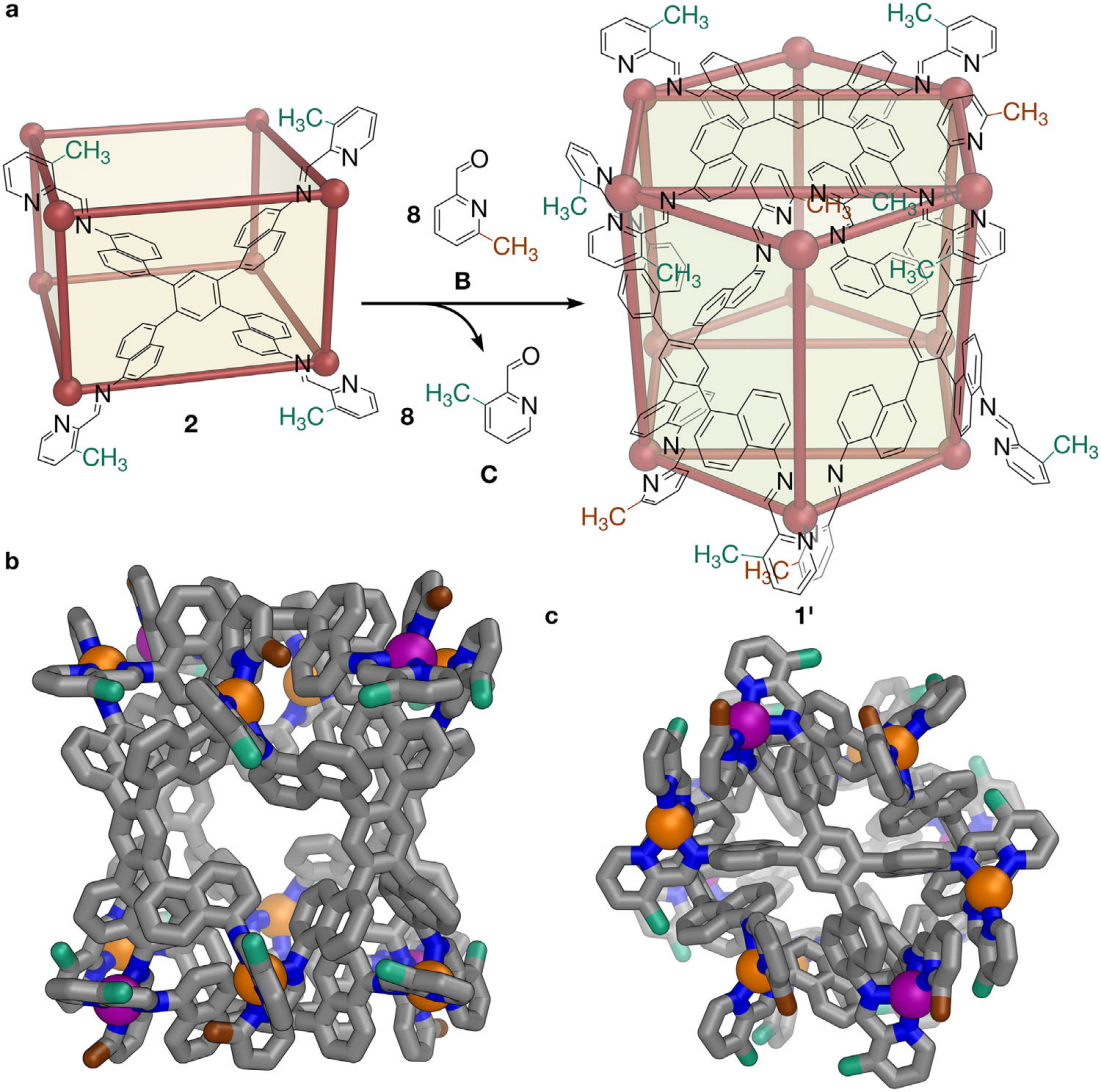
(a), The structural reconfiguration from **2** to **1’**, a [Cu^I^_12_L_6_]^12+^ cage analogous to **1**, by displacement of eight 3‐methyl‐2‐formylpyridine (C) residues with 6‐methyl‐2‐formylpyridine (**B)** per [Cu^I^_12_L_6_]^12+^ cage, as inferred from experimental results. (b) Top and (c) side views of the model of the putative [Cu^I^_12_L_6_]^12+^ cage generated by the structural reconfiguration shown in **a**, using Python with RDKit [67] and NumPy [68]. Based on the hypothetical [Cu^I^_12_L_6_]^12+^ structure **1’** analogous to **1** shown in Figure S35b, eight 3‐methyl‐2‐formylpyridine (C) residues must be displaced by 6‐methyl‐2‐formylpyridine (B) to remove the steric hindrance that destabilizes the structure, successfully driving the structural reconfiguration. Color scheme: C = gray, brown (methyl groups in 6‐methyl‐2‐formylpyridine (B) residues), or green (methyl groups in 3‐methyl‐2‐formylpyridine (C) residues); H = white; N = blue; Δ‐Cu = purple; Λ‐Cu = orange. Hydrogen atoms, counterions, and solvent molecules have been omitted for clarity.

This study demonstrates the use of secondary interactions and steric factors to control structural outcomes in Cu^I^ coordination‐directed self‐assembly. Using tetramine subcomponent **A** with 1,5‐naphthylene panels resulted in two distinct structures being obtained: a [Cu^I^_12_L_6_]^12+^ pseudo‐hexagonal prism **1**, stabilized by 29–32 C─H···π interactions and 12 pairs of aromatic stacking interactions, and a [Cu^I^_8_L_4_]^8+^ rectangular open prism **2** that formed when 3‐methyl‐2‐formylpyridine (**C**) was used to create steric clashes that hinder these interactions. The structural reconfiguration from **2** to a [Cu^I^_12_L_6_]^12+^ cage analogous to **1** was achieved by adding 6‐methyl‐2‐formylpyridine (**B**) to displace 3‐methyl‐2‐formylpyridine (**C**) residues, where ∼8 out of 24 aldehyde residues were replaced per [Cu^I^_12_L_6_]^12+^ cage, consistent with the predicted number of sterically hindered positions. These findings show that cage structure can be controlled by balancing favorable interactions with steric effects, providing design principles for generating distinct architectures from identical building blocks.

## Conflicts of Interest

The authors declare no conflict of interest.

## Supporting information



The authors have cited additional references within the Supporting Information [65, 66, 67, 69, 70, 71, 73, 74, 75, 76, 77, 78, 79, 80, 81, 82, 83, 84, 85, 86, 87, 88]. The scripts involved in this study can be found at https://github.com/houyang‐xu/15‐Naphthylene‐Cu‐Structures.
**Supporting File 1**: anie72157‐sup‐0001‐SuppMat.docx.

**Supporting File 2**: anie72157‐sup‐0002‐Data.zip.

## Data Availability

The data that supports the findings of this study are available in the supplementary material of this article. The scripts involved in this study can be found at https://github.com/houyang‐xu/15‐Naphthylene‐Cu‐Structures.
