# Supplementary material for: Steric Hindrance and Secondary Interactions Govern Reconfiguration Between Two Complex CuI Coordination Cages
Source: Angew Chem Int Ed Engl. 2026 Apr 14;65(21):e4287066. doi: 10.1002/anie.4287066 (PMC13182205; doi:10.1002/anie.4287066)
Supplement: Supplementary file 1 — The authors have cited additional references within the Supporting Information [65, 66, 67, 69, 70, 71, 73, 74, 75, 76, 77, 78, 79, 80, 81, 82, 83, 84, 85, 86, 87, 88]. The scripts involved in this study can be found at https://github.com/houyang‐xu/15‐Naphthylene‐Cu‐Structures. Supporting File 1: anie72157‐sup‐0001‐SuppMat.docx. [file ANIE-65-e4287066-s001.docx]

Table of Contents

[Table of Contents 1](#_Toc225293992)

[1 General Information 1](#_Toc225293993)

[2 Synthesis and characterization 2](#_Toc225293994)

[2.1 Subcomponent **A** 2](#_Toc225293995)

[2.2 Pseudo-hexagonal prism **1**·X 8](#_Toc225293996)

[2.3 Rectangular open prism **2** 23](#_Toc225293997)

[3 X-ray crystallography 36](#_Toc225293998)

[4 Analysis of Secondary Interactions and Steric Hindrance 43](#_Toc225293999)

[4.1 Analysis of Secondary Interactions 43](#_Toc225294000)

[4.2 Analysis of Steric Hindrance 50](#_Toc225294001)

[5 Reconfiguration from **2** to generate **1** 54](#_Toc225294002)

[6 Volume calculation 60](#_Toc225294003)

[7 Self-assembly and reconfiguration attempts with analogous subcomponents 62](#_Toc225294004)

[7.1 Self-assembly attempt towards analog of cage **1** with 6-fluoro-2-formylpyridine (**D**) 62](#_Toc225294005)

[7.2 Self-assembly attempt towards analog of cage **2** with 3-bromo-2-formylpyridine (**E**) 64](#_Toc225294006)

[7.3 Reconfiguration from the assembly mixture with 3-bromo-2-formylpyridine (**E**) to generate analogs of **1** 67](#_Toc225294007)

[References 73](#_Toc225294008)

# 1 General Information

Unless specified differently, all chemicals were purchased from commercial suppliers and used without further purification unless stated otherwise. NMR spectra were recorded using NMR spectrometers including: Bruker DRX-400, Bruker Avance 500 Cryo, Bruker 500 TCI-ATM Cryo, 500 MHz AVIII HD Smart Probe, and Bruker 700 TCI-ATM Cryo. Chemical shifts (δ) for ^1^H NMR spectra are reported in parts per million (ppm) and are reported relative to the solvent residual peak.

Signal multiplicity in ^1^H and ^13^C NMR spectra were reported with the following abbreviations: singlet (s), doublet (d), triplet (t), doublet of doublets (dd), triplet of doublets (td), doublet of doublet of doublets (ddd), multiplet (m), broad (br) and apparent (app.). HSQC spectra were recorded with edited pulse sequence, rendering CH/CH_3_ and CH_2_ cross peaks with opposite phases. All ^1^H DOSY experiments were run on a Bruker 400 MHz Avance III HD Smart Probe spectrometer. The maximum gradient strength was 5.35 G/cm A. These experiments were run with a standard Bruker pulse program, ledbpgp2s, employing a stimulated echo and longitudinal eddy-current delay (LED) using bipolar gradient pulses for diffusion using 2 spoil gradients. Rectangular gradients were used with a total duration of 1.5 ms. A gradient ramp of 10% or 90% were utilized. Gradient recovery delays were 1100 μs. Data were processed with Bruker Dynamic Center 2.8.0.1 with diffusion coefficients calculated as the mean value for all the peaks identified.

All high-resolution electrospray ionization mass spectra (ESI-HRMS) in this study were recorded using a Waters Synapt G2-Si instrument.

# 2 Synthesis and characterization

## 2.1 Subcomponent A

Scheme S1. The synthetic route of subcomponent A.

**S1** (Tert-butyl (5-(4,4,5,5-tetramethyl-1,3,2-dioxaborolan-2-yl)naphthalen-1- yl)carbamate) was synthesized from commercially available 1-amino-5-bromonaphthylene via a reported method.^[1]^

1,2,4,5-Tetrabromobenzene (100 mg, 0.254 mmol, 1.00 equiv.), **S1** (469 mg, 1.27 mmol, 5.0 equiv.), palladium diacetate (2.85 mg, 0.0127 mmol, 0.05 equiv.), triphenylphosphine (6.66 mg, 0.0254 mmol, 0.10 equiv.), and barium hydroxide octahydrate (801 mg, 2.54 mmol, 10.0 equiv.) were added to a Schlenk flask. A mixture of THF/H_2_O (3:1, 30 mL) was added to the flask, and the resulting solution was bubbled with N_2_ for 15 min before the mixture was heated at reflux for 40 h under nitrogen. After cooling to room temperature, the mixture was concentrated *in* *vacuo*, extracted with ethyl acetate (30 mL × 4), dried over MgSO_4_, and concentrated *in* *vacuo* again. A mixture of dichloromethane/petroleum ether (1:4, 40 mL) was added to the resulting solid. The suspension was sonicated and then centrifuged. The supernatant was removed, and the same process was repeated on the precipitate twice. The resulting solid was suspended in dichloromethane (5 mL), cooled to 0°C, and trifluoroacetic acid (1 mL) was added. The mixture was then stirred at room temperature for 16 h. The mixture was concentrated *in* *vacuo*, diluted with saturated sodium bicarbonate solution (50 mL), and filtered under reduced pressure. The residue was washed with dichloromethane/petroleum ether (1:3, 30 mL × 3), dried *in* *vacuo*, and obtained as a pale grey powder. Yield: 163 mg (quantitative over 2 steps).


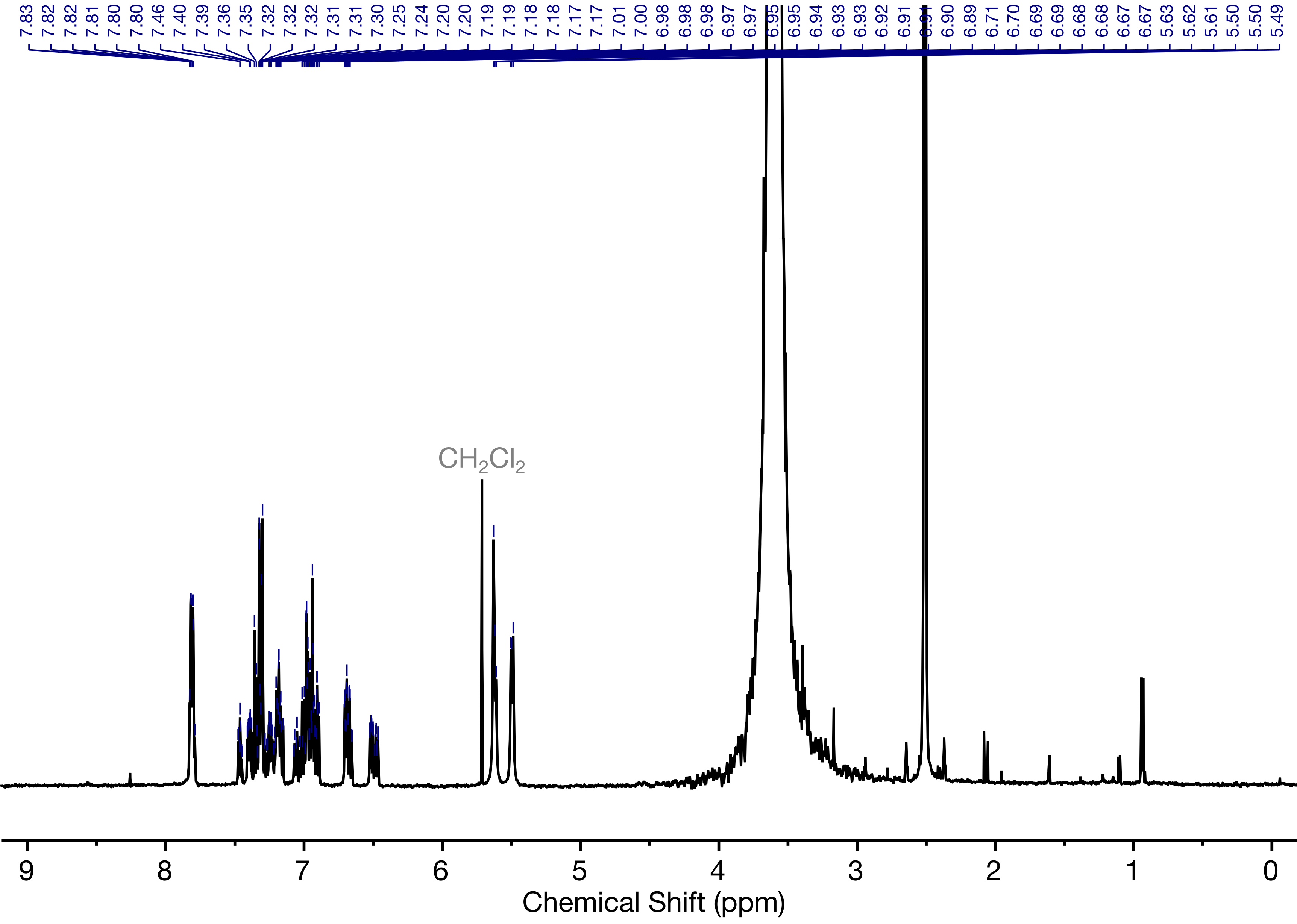


**Figure S1.** ^1^H NMR Spectrum (500 MHz, DMSO-*d_6_*, 298 K) of subcomponent A.

As shown in Figure S1, the ^1^H NMR spectrum of subcomponent **A** exhibits a much higher number of signals than expected, indicating the presence of multiple isomeric species. The sterically crowded nature of **A** is likely to be the cause this complex spectrum through atropisomerism. Collecting data at 373 K caused the signals to coalesce into two sets, as shown in Figure S2. Assignment of these signals at 373 K has been summarized below, but the high degree of exchange does not allow differentiating these signals into each isomer.

**^1^H NMR** (500 MHz, DMSO-*d*_6_, 373 K): δ 7.81-7.76 (*m*, 4H), 7.47-6.86 (*m*, 20H), 6.74-6.45 (*m*, 4H), 5.37-5.10 (*m*, 8H).

**^13^C NMR** (126 MHz, DMSO-*d*_6_, 373 K): δ 145.1, 144.7, 139.4, 139.4, 138.8, 138.7,

135.1, 134.9, 133.9, 133.2, 128.5, 128.4, 127.1, 126.7, 126.6, 126.4, 123.6, 122.9,

122.8, 121.8, 115.0, 114.8, 108.3, 108.2.

**ESI-HRMS** calcd *m/z* = 643.2856 for [C_36_H_28_N_3_]^+^, found *m/z* = 643.2900.


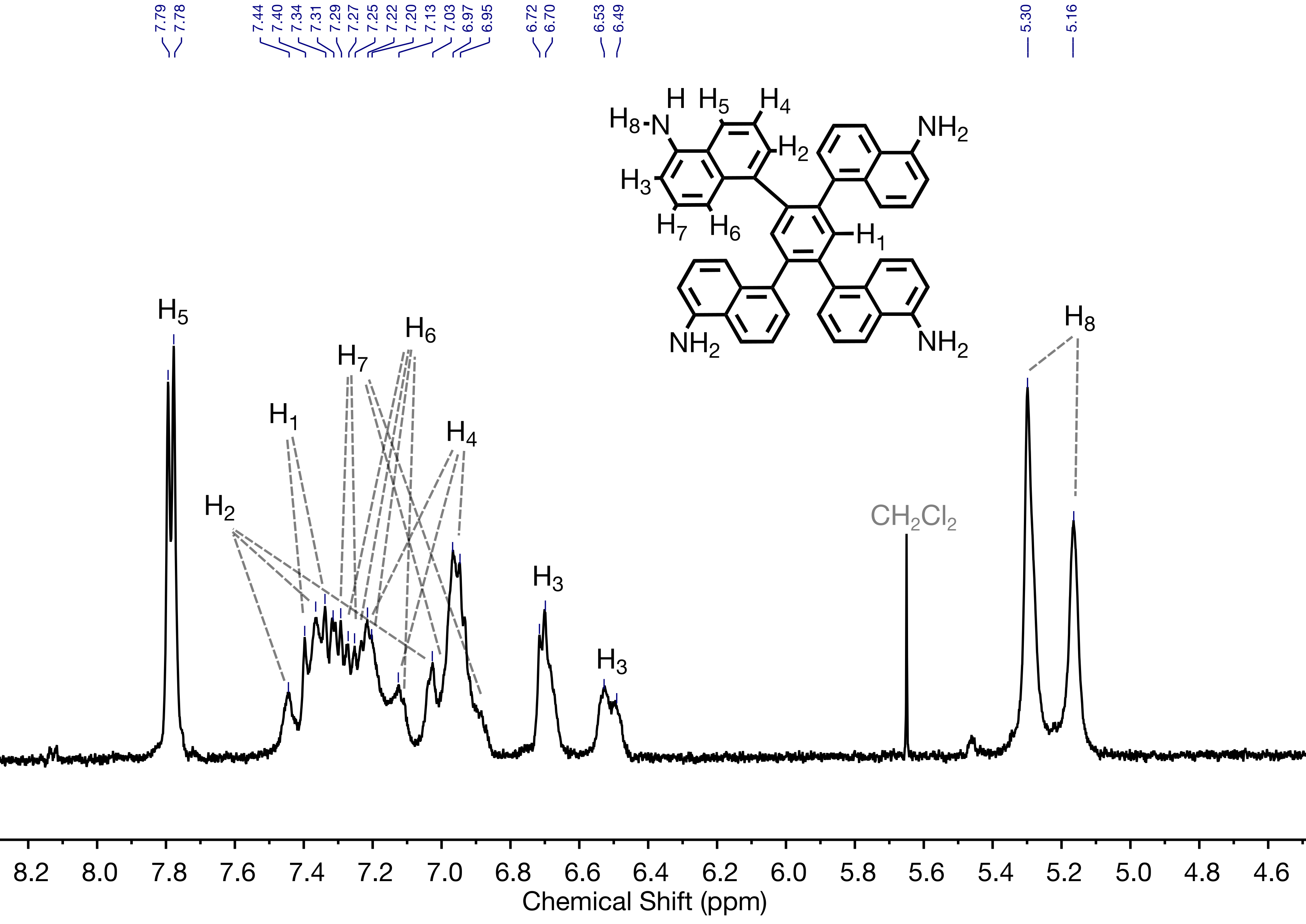


**Figure S2.** Aromatic region of the ^1^H NMR Spectrum (500 MHz, DMSO-*d_6_*, 373 K) of subcomponent A.


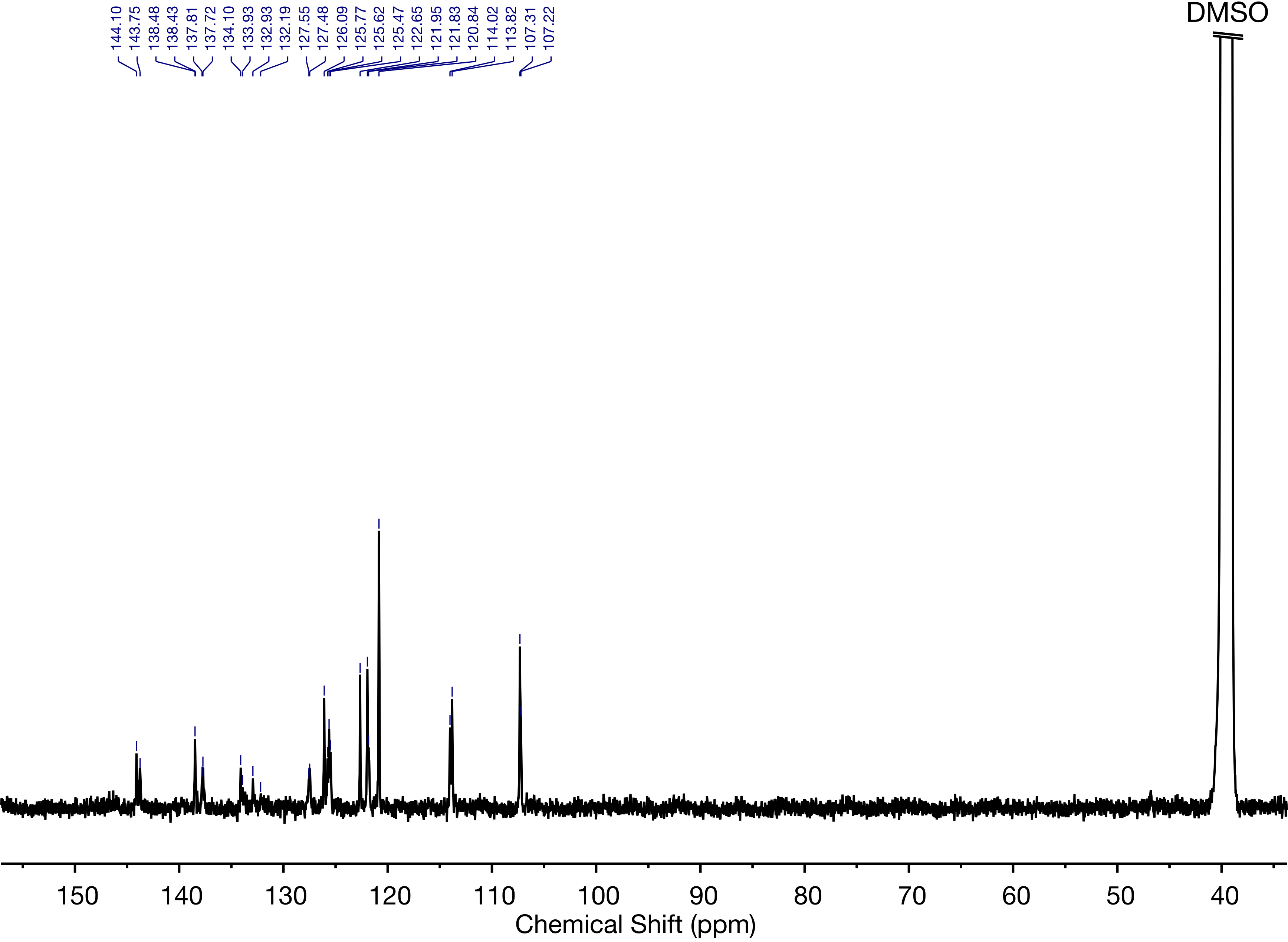


**Figure S3.** ^13^C NMR Spectrum (126 MHz, DMSO-*d_6_*, 373 K) of subcomponent A.


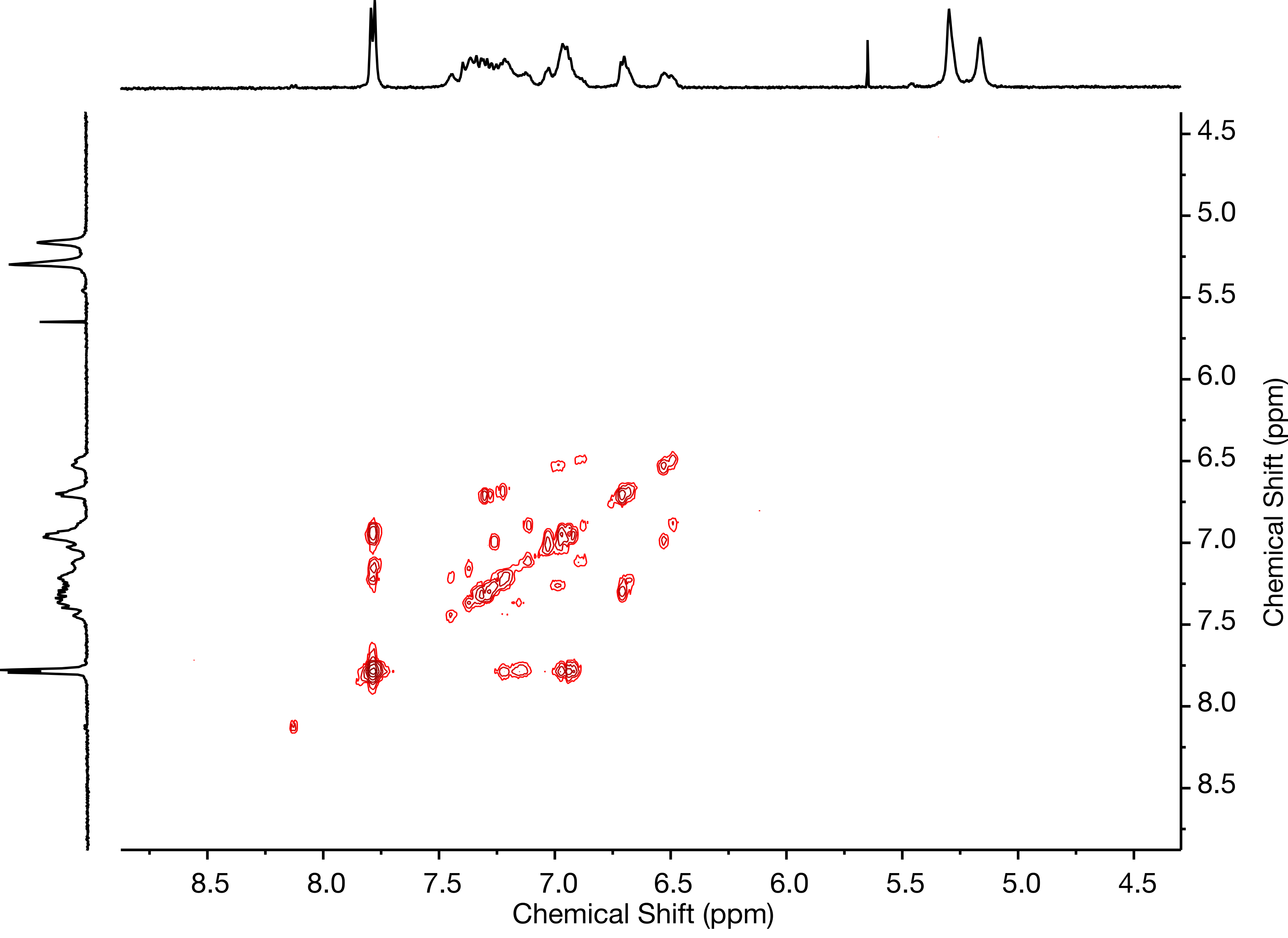


Figure S4. Aromatic region of the ^1^H-^1^H DQF-COSY spectrum (500 MHz, DMSO-*d_6_*, 373 K) of subcomponent A.


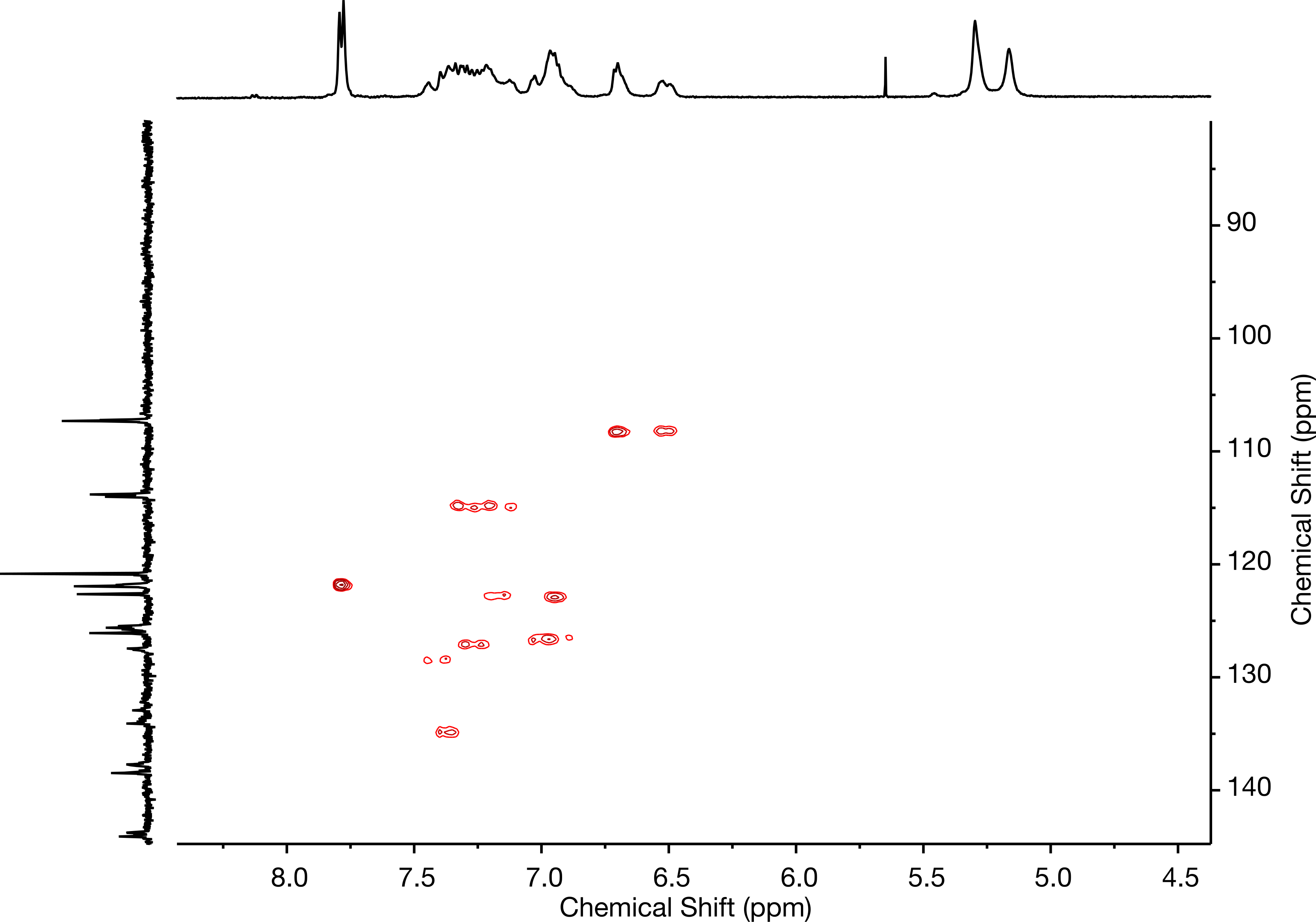


Figure S5. Aromatic region of the ^1^H-^13^C HSQC spectrum (500 MHz, DMSO-*d_6_*, 373 K) of subcomponent A.


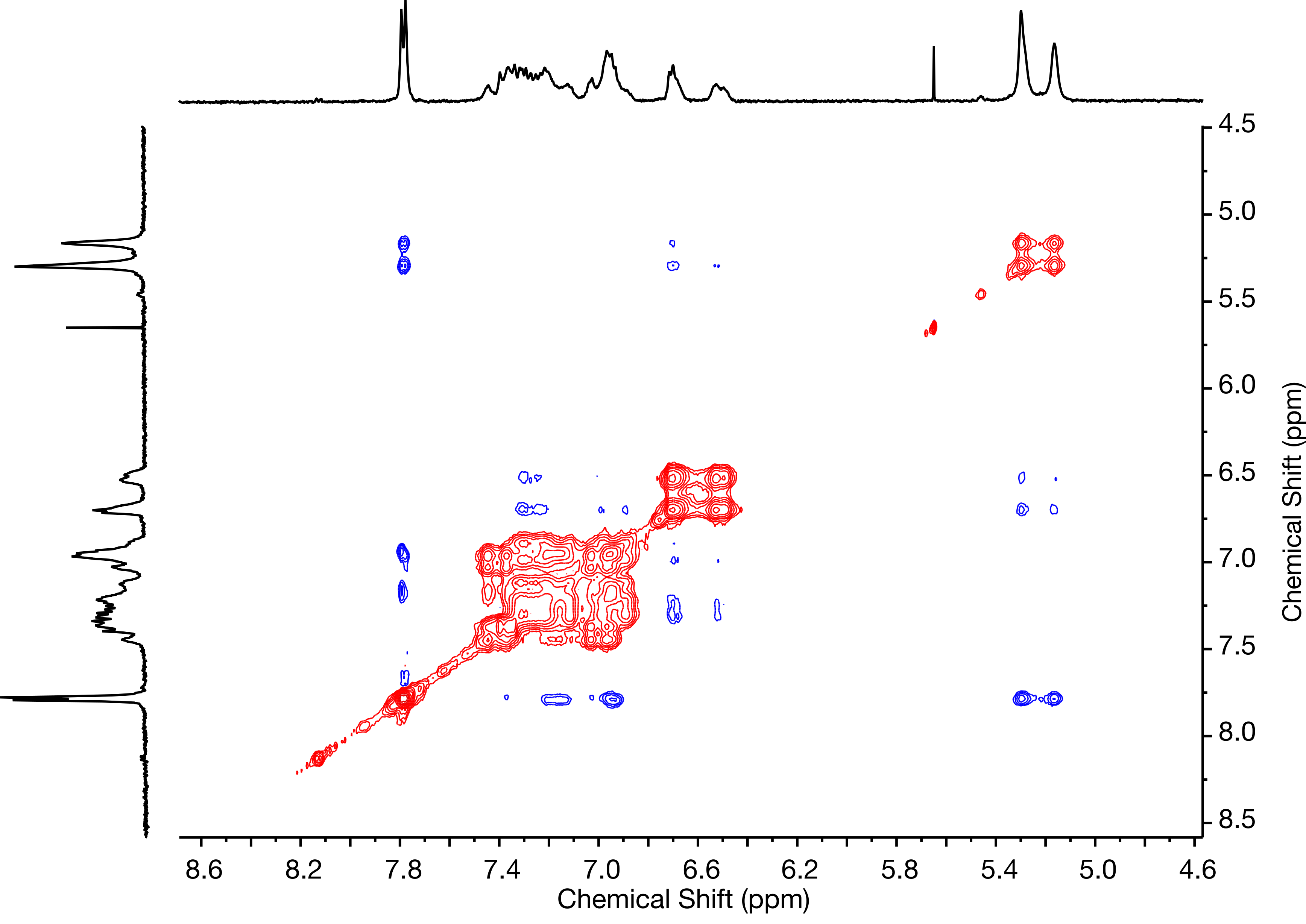


Figure S6. Aromatic region of the ^1^H-^1^H ROESY spectrum (500 MHz, DMSO-*d_6_*, 373 K) of subcomponent A.

## 2.2 Pseudo-hexagonal prism 1·X


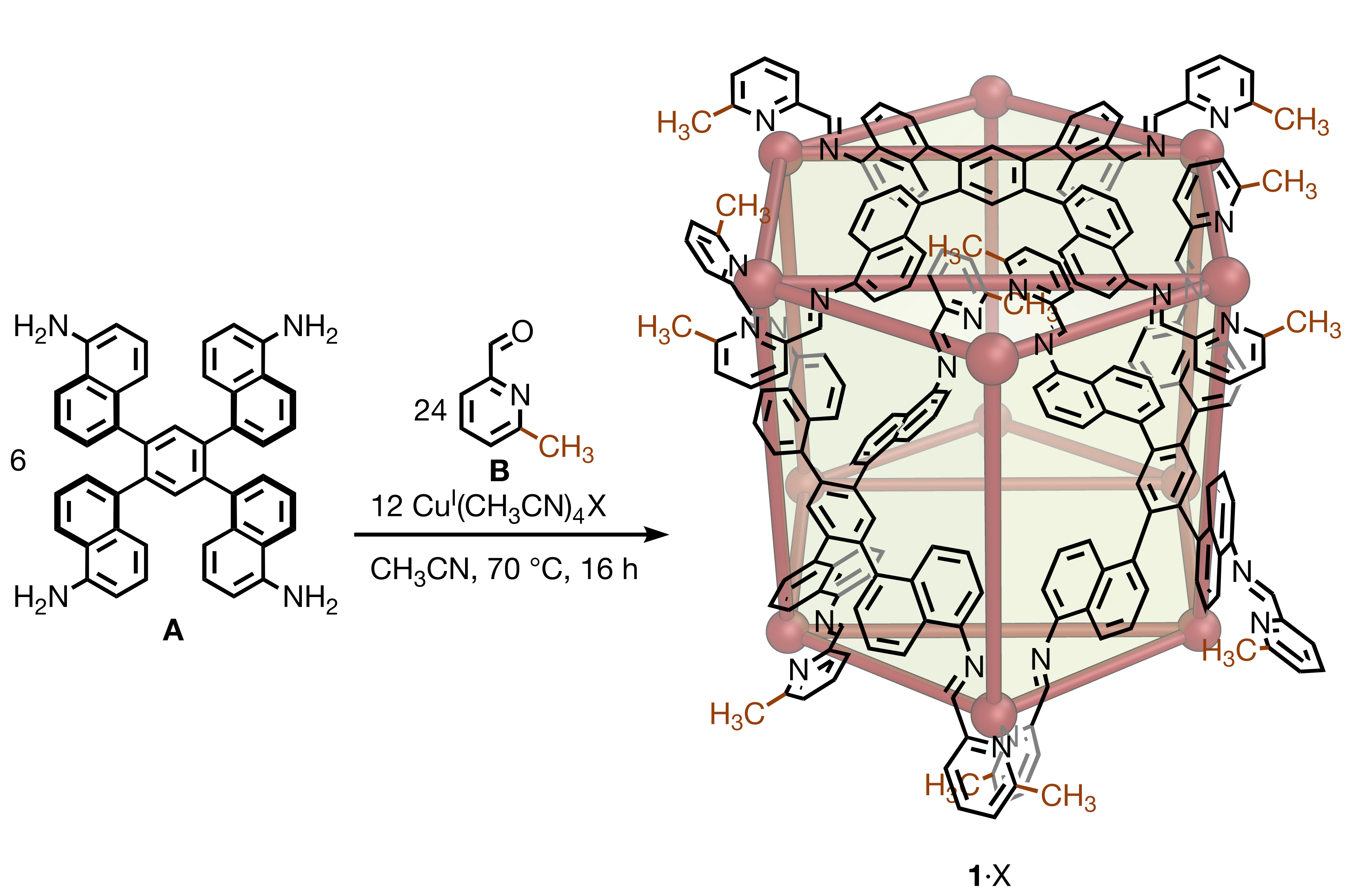


**Scheme S2.** The self-assembly of pseudo-hexagonal prism 1.

The preparation and characterization of **1**·(BF_4_)_12_ is summarized as below:

Tetramine subcomponent **A** (4.11 mg, 6.39 µmol, 1.0 equiv.) and Cu(CH_3_CN)_4_BF_4_ (4.02 mg, 12.8 µmol, 2.0 equiv.) were mixed in a Schlenk tube before a solution of 6-methyl-2-formylpyridine (**B**) in freshly distilled acetonitrile (60.0 mmol·L^-1^, 0.5 mL, 4.4 equiv.) was added. The mixture was degassed via three freeze-pump-thaw cycles, before being heated at 70 °C for 16 hours. The reaction was cooled to room temperature, concentrated to 0.2 mL under a flow of N_2_, and the product precipitated by addition of diethyl ether (15 mL). The mixture was then centrifuged and the supernatant was discarded. The precipitate was washed with diethyl ether (3 × 15 mL), dried *in vacuo*, affording cage **1**·(BF_4_)_12_ as a dark brown solid (8.23 mg, 1.01 µmol, 95%).

**^1^H NMR** (500 MHz, CD_3_CN, 298 K): δ = 8.88 (d, 4H, *J* = 9.0 Hz), 8.52 (d, 4H, *J* = 8.8 Hz), 8.32–8.29 (m, 8H), 8.26–8.13 (m, 24H*), 8.06–8.02 (m, 8H*), 8.01–7.92 (m, 28H*), 7.90–7.87 (m, 4H*), 7.85 (d, 4H*, *J* = 8.6 Hz), 7.85 (d, 4H*, *J* = 8.1 Hz), 7.76–7.72 (m, 8H*), 7.71 (s, 4H), 7.69–7.66 (m, 8H*), 7.63–7.59 (m, 12H*), 7.54 (d, 4H*, *J* = 7.5 Hz), 7.51–7.48 (m, 4H*), 7.46 (s, 4H), 7.43 (m, 4H*), 7.40 (s, 4H*), 7.36 (s, 4H*), 7.31–7.28 (m, 8H*), 7.25 (s, 4H*), 7.23–7.22 (m, 4H*), 7.17 (d, 4H*, *J* = 6.8 Hz), 7.14–7.10 (m, 12H*), 7.05–6.99 (m, 8H), 6.98–6.94 (m, 4H*), 6.94–6.90 (m, 4H*), 6.88–6.82 (m, 8H*), 6.50–6.44 (m, 8H), 6.20–6.16 (m, 4H), 6.11–6.06 (m, 4H), 6.05–6.00 (m, 4H), 5.93 (s, 4H), 5.42 (d, 4H, *J* = 7.5 Hz), 5.32 (s, 4H), 5.28–5.24 (m, 8H), 4.46 (d, 4H, *J* = 7.1 Hz), 4.23 (d, 4H, *J* = 7.0 Hz), 4.17–4.11 (m, 8H), 3.22 (s, 12H), 2.73 (s, 12H), 2.46 (s, 12H), 2.45 (s, 12H), 1.92 (s, 12H), 1.87 (s, 12H). *The relative intensities of these signals in the ^1^H NMR spectrum are slightly higher than expected (and quoted) due to signal overlap.

**^13^C NMR** (126 MHz, CD_3_CN, 298 K): δ 167.4, 165.9, 160.2, 159.6, 159.6, 159.5, 159.3, 159.2, 158.1, 156.8, 156.8, 156.0, 156.0, 150.9, 150.1, 149.9, 149.5, 149.4, 148.9, 148.0, 147.2, 146.3, 144.9, 143.1, 142.4, 141.3, 140.8, 140.4, 140.0, 139.8, 139.6, 139.4, 139.3, 139.1, 139.0, 138.8, 138.8, 138.7, 138.7, 137.4, 136.9, 135.2, 134.8, 134.1, 133.7, 133.5, 133.1, 132.8, 132.5, 132.0, 131.5, 131.2, 131.2, 131.1, 130.8, 130.8, 130.6, 130.3, 130.0, 129.9, 129.5, 129.5, 128.5, 128.3, 128.0, 127.9, 127.9, 127.9, 127.8, 127.7, 127.6, 127.4, 127.4, 127.3, 127.3, 127.2, 127.0, 126.8, 126.8, 126.4, 126.3, 126.3, 126.2, 126.1, 126.0, 125.7, 125.6, 125.4, 125.2, 124.0, 123.7, 123.4, 123.0, 122.5, 122.2, 122.2, 122.0, 121.7, 120.7, 116.4, 115.8, 115.7, 28.2, 27.7, 26.5, 26.4, 25.5, 24.3.

**ESI-LRMS** [**1**·(BF_4_)_12_] **=** (C_46_H_34_N_4_(C_7_H_5_N)_4_)_6_Cu_12_(BF_4_)_12_) *m/z* = 727.1 [**1**·(BF_4_)_2_]^10+^ (calc. 726.8) 817.6 [**1**·(BF_4_)_3_]^9+^ (calc. 817.2) 930.6 [**1**·(BF_4_)_4_]^8+^ (calc. 930.2) 1076.0 [**1**·(BF_4_)_5_]^7+^ (calc. 1075.5) 1269.8 [**1**·(BF_4_)_6_]^6+^ (calc. 1269.2) 1540.1 [**1**·(BF_4_)_7_]^5+^ (calc. 1540.4)

**ESI-HRMS** [**1**·(BF_4_)_12_] **=** (C_46_H_34_N_4_(C_7_H_5_N)_4_)_6_Cu_12_(BF_4_)_12_) *m/z* = 817.2062 [**1**·(BF_4_)_3_]^9+^ (calc. 817.1800) 930.2321 [**1**·(BF_4_)_4_]^8+^ (calc. 930.1781) 1075.4093 [**1**·(BF_4_)_5_]^7+^ (calc. 1075.4615) 1269.3113 [**1**·(BF_4_)_6_]^6+^ (calc. 1269.1726) 1540.1906 [**1**·(BF_4_)_7_]^5+^ (calc. 1540.3681)


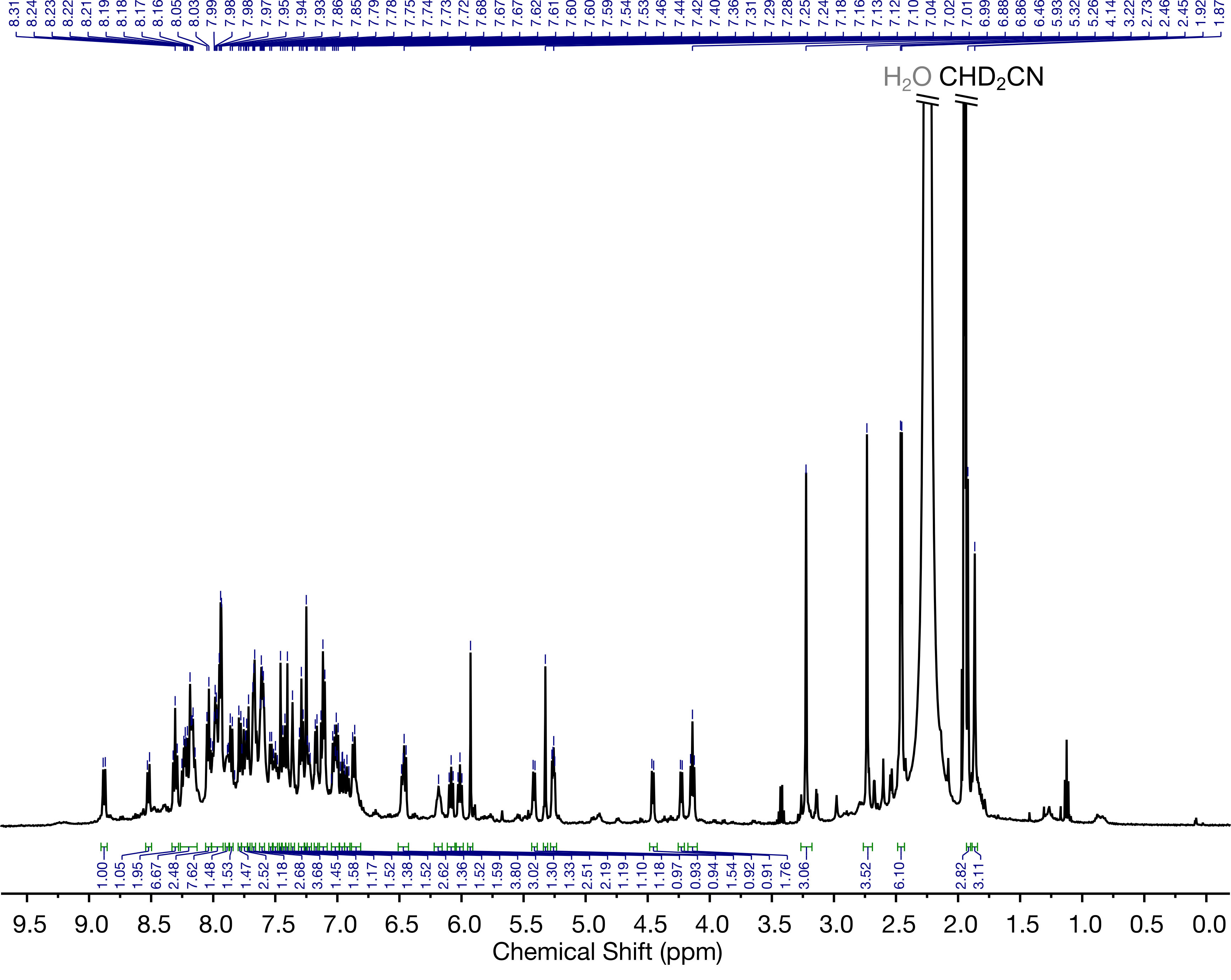


Figure S7. ^1^H NMR Spectrum (500 MHz, CD_3_CN, 298 K) of pseudo-hexagonal prismatic cage 1·(BF_4_)_12_.

The ¹H NMR signals of cage **1**·(BF_4_)_12_ are assigned and visualized in Figure S8, with detailed assignments listed in Table S1:


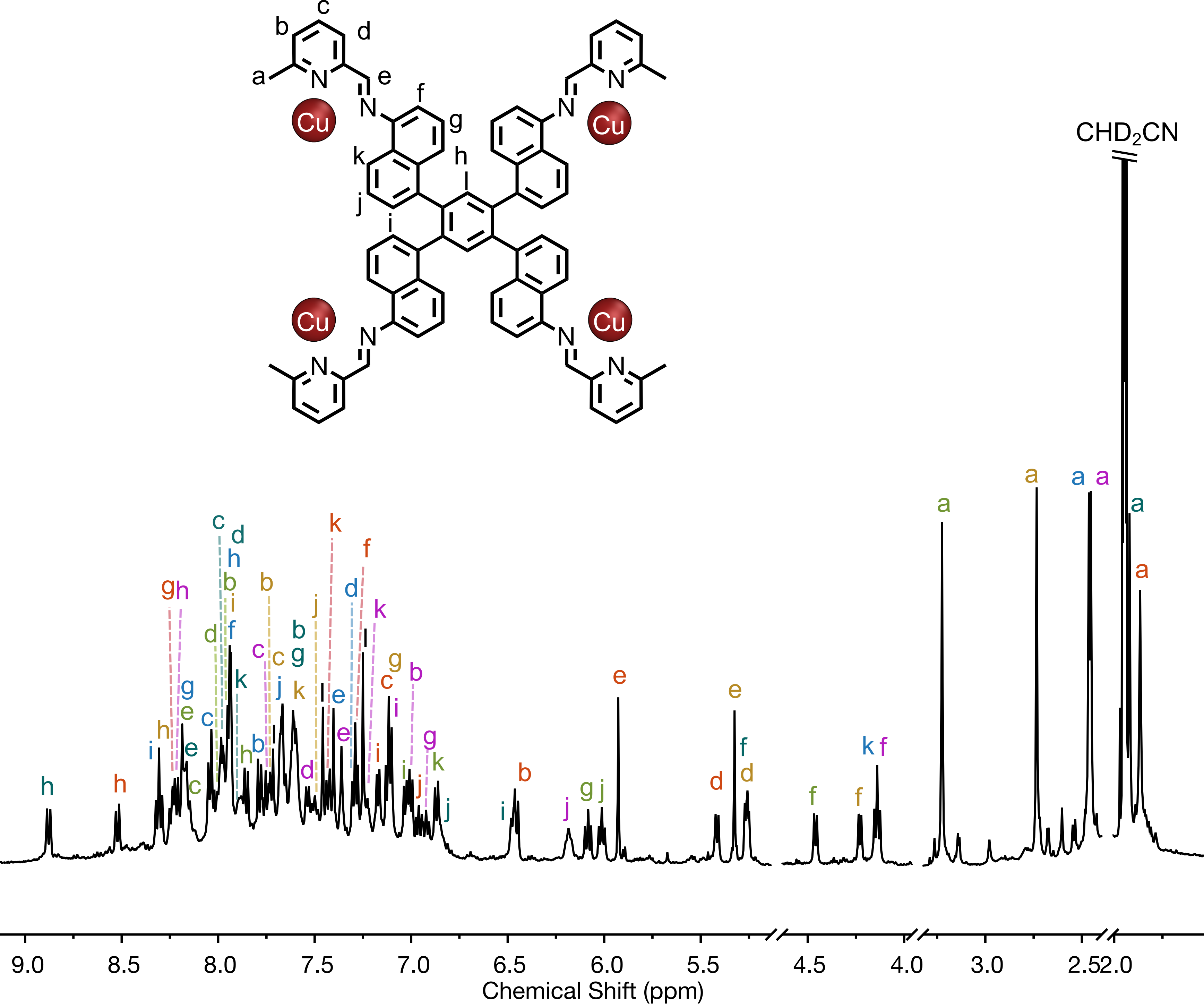


Figure S8. Aromatic region of the ^1^H NMR spectrum (500 MHz, CD_3_CN, 298 K) of cage 1·(BF_4_)_12_, with assignment of signals. The signals of protons in each distinct ligand arm could be distinguished and are marked with different colours. It was not possible to definitively attribute each set to a particular ligand arm within the structure due to the lack of a clear starting point for such assignments. However, the sets of signals highlighted with red and yellow labels are suspected to be highly likely those from the ligand capping the pseudo-hexagonal faces of the cage, due to their unusually strongly shielded imine signals.

Table S1. ^1^H NMR signal assignments for 1·(BF_4_)_12_ (500 MHz, CD₃CN, 298 K). The signals of protons in each distinct ligand arm could be distinguished and are colour-coded as in Figure S8: red, yellow, green, teal, blue, and magenta, with protons on the central 1,2,4,5-tetrasubstituted benzene rings in black. It was not possible to definitively attribute each set to a particular ligand arm within the structure.

| **Chemical Shift (δ, ppm)** | **Multiplicity** | **No. of H** | ***J* (Hz)** | **Assignment** |
| --- | --- | --- | --- | --- |
| 8.88 | d | 4H | 9.0 | h |
| 8.52 | d | 4H | 8.8 | h |
| 8.32–8.29 | m | 8H |  | i, h |
| 8.26–8.13 | m | 24H* |  | g, h, g, e, e, c |
| 8.06–8.02 | m | 8H* |  | c, d |
| 8.01–7.92 | m | 24H* |  | c, b, h, i, f, d |
| 7.90–7.87 | m | 4H* |  | k |
| 7.85 | d | 4H* | 8.6 | h |
| 7.85 | d | 4H* | 8.1 | b |
| 7.76–7.72 | m | 8H* |  | c, b |
| 7.71 | s | 4H |  | l |
| 7.69–7.66 | m | 8H* |  | c, j |
| 7.63–7.59 | m | 12H* |  | b, g, k |
| 7.54 | d | 4H* | 7.5 | d |
| 7.51–7.48 | m | 4H* |  | j |
| 7.46 | s | 4H |  | l |
| 7.43 | m | 4H* |  | k |
| 7.40 | s | 4H* |  | e (imine) |
| 7.36 | s | 4H* |  | e (imine) |
| 7.31–7.28 | m | 8H* |  | d, f |
| 7.25 | s | 4H* |  | l |
| 7.23–7.22 | m | 4H* |  | k |
| 7.17 | d | 4H* | 6.8 | i |
| 7.14–7.10 | m | 12H* |  | c, g, i |
| 7.05–6.99 | m | 8H |  | i, b |
| 6.98–6.94 | m | 4H* |  | j |
| 6.94–6.90 | m | 4H* |  | g |
| 6.88–6.82 | m | 8H* |  | k, j |
| 6.50–6.44 | m | 8H |  | i, b |
| 6.20–6.16 | m | 4H |  | j |
| 6.11–6.06 | m | 4H |  | g |
| 6.05–6.00 | m | 4H |  | j |
| 5.93 | s | 4H |  | e (imine) |
| 5.42 | d | 4H | 7.5 | d |
| 5.32 | s | 4H |  | e (imine) |
| 5.28–5.24 | m | 8H |  | f, d |
| 4.46 | d | 4H | 7.1 | f |
| 4.23 | d | 4H | 7.0 | f |
| 4.17–4.11 | m | 8H |  | k, f |
| 3.22 | s | 12H |  | a (CH_3_) |
| 2.73 | s | 12H |  | a (CH_3_) |
| 2.46 | s | 12H |  | a (CH_3_) |
| 2.45 | s | 12H |  | a (CH_3_) |
| 1.92 | s | 12H |  | a (CH_3_) |
| 1.87 | s | 12H |  | a (CH_3_) |

* These protons have slightly higher relative intensities than expected due to signal overlap.


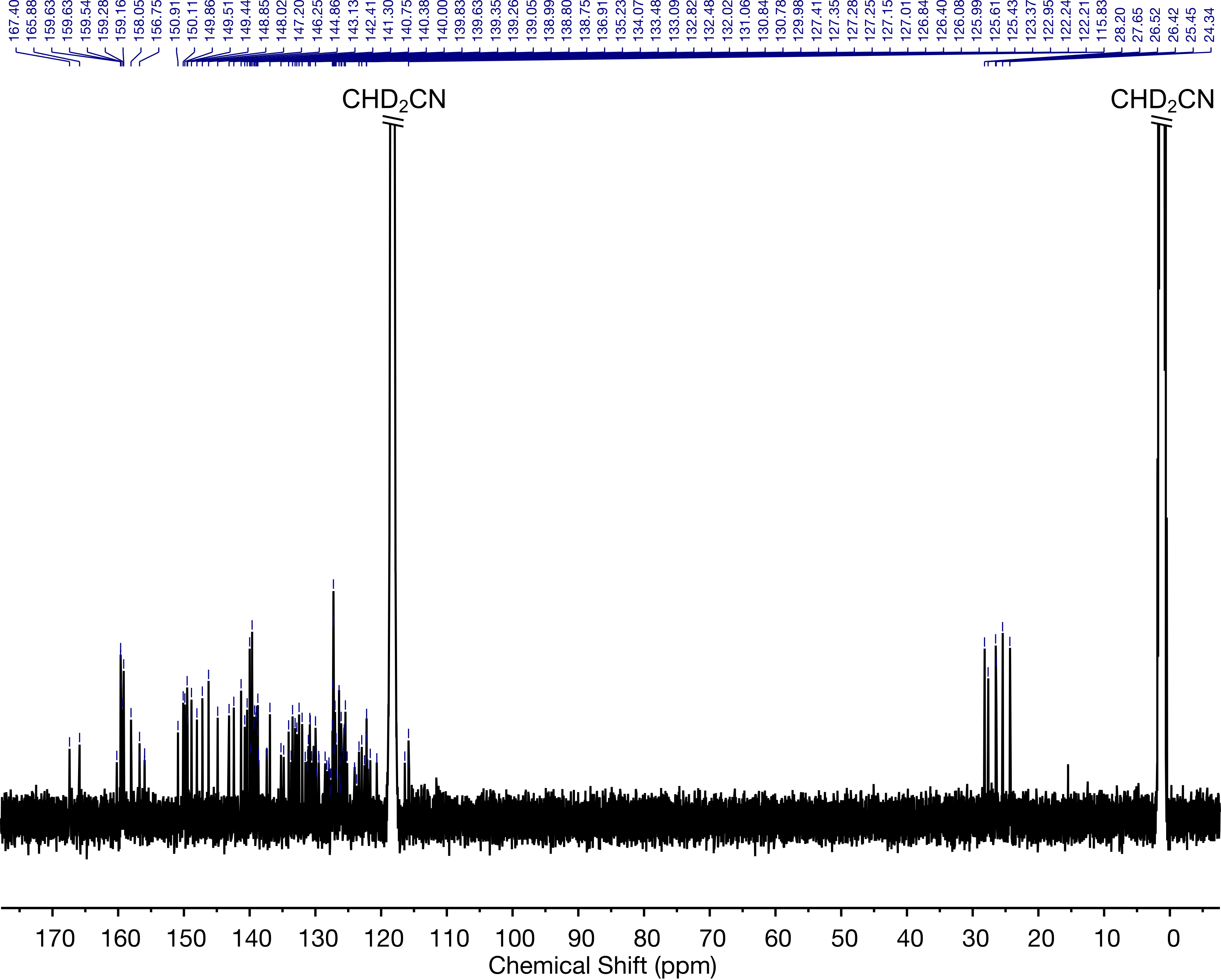


Figure S9. ^13^C NMR Spectrum (126 MHz, CD_3_CN, 298 K) of pseudo-hexagonal prismatic cage 1·(BF_4_)_12_.


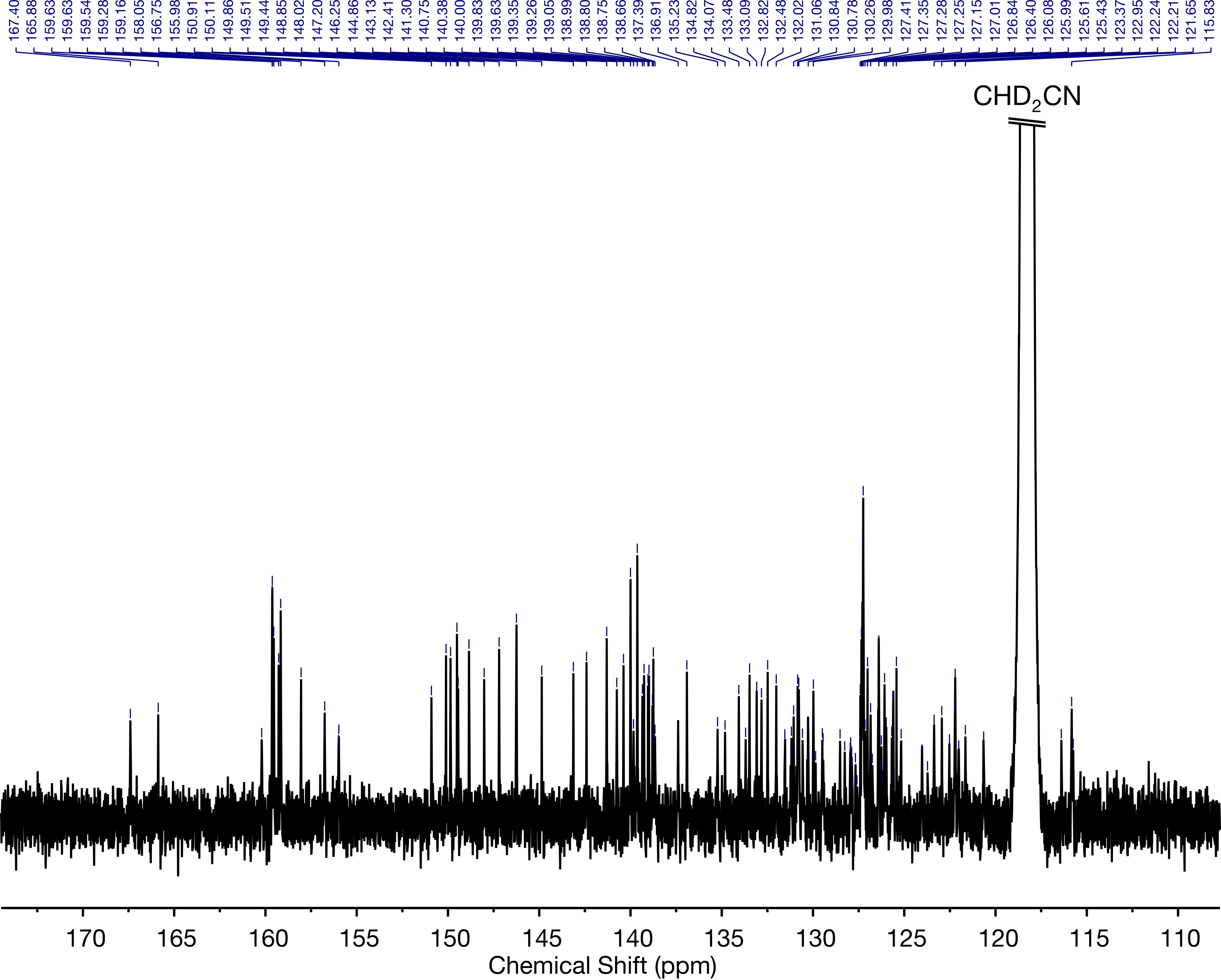


Figure S10. Aromatic region of the ^13^C NMR Spectrum (126 MHz, CD_3_CN, 298 K) of pseudo-hexagonal prismatic cage 1·(BF_4_)_12_.


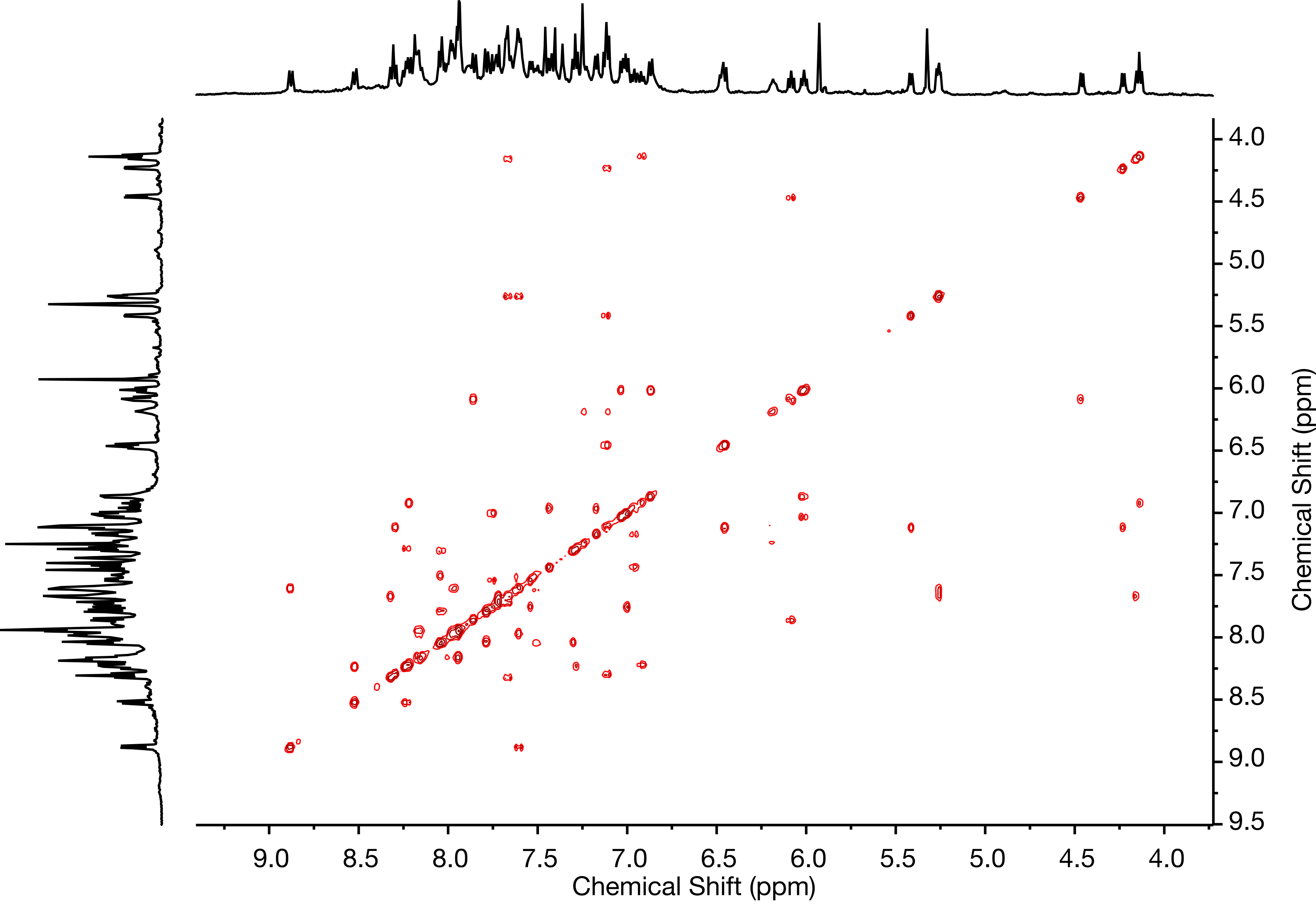


Figure S11. Aromatic region of the ^1^H-^1^H DQF-COSY spectrum (500 MHz, CD_3_CN, 298 K) of pseudo-hexagonal prismatic cage 1·(BF_4_)_12_.


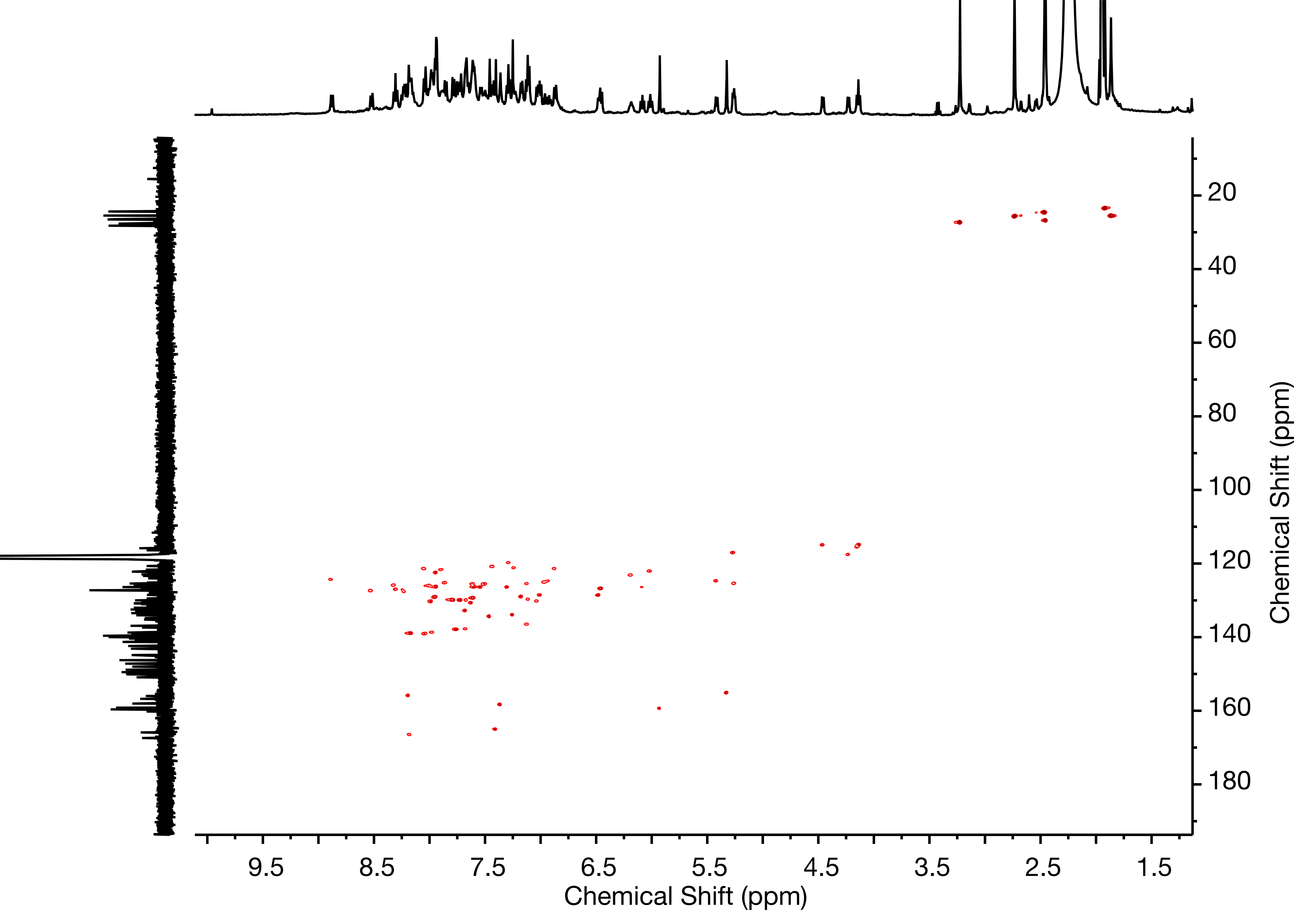


Figure S12. ^1^H-^13^C HSQC spectrum (500 MHz, CD_3_CN, 298 K) of pseudo-hexagonal prismatic cage 1·(BF_4_)_12_.


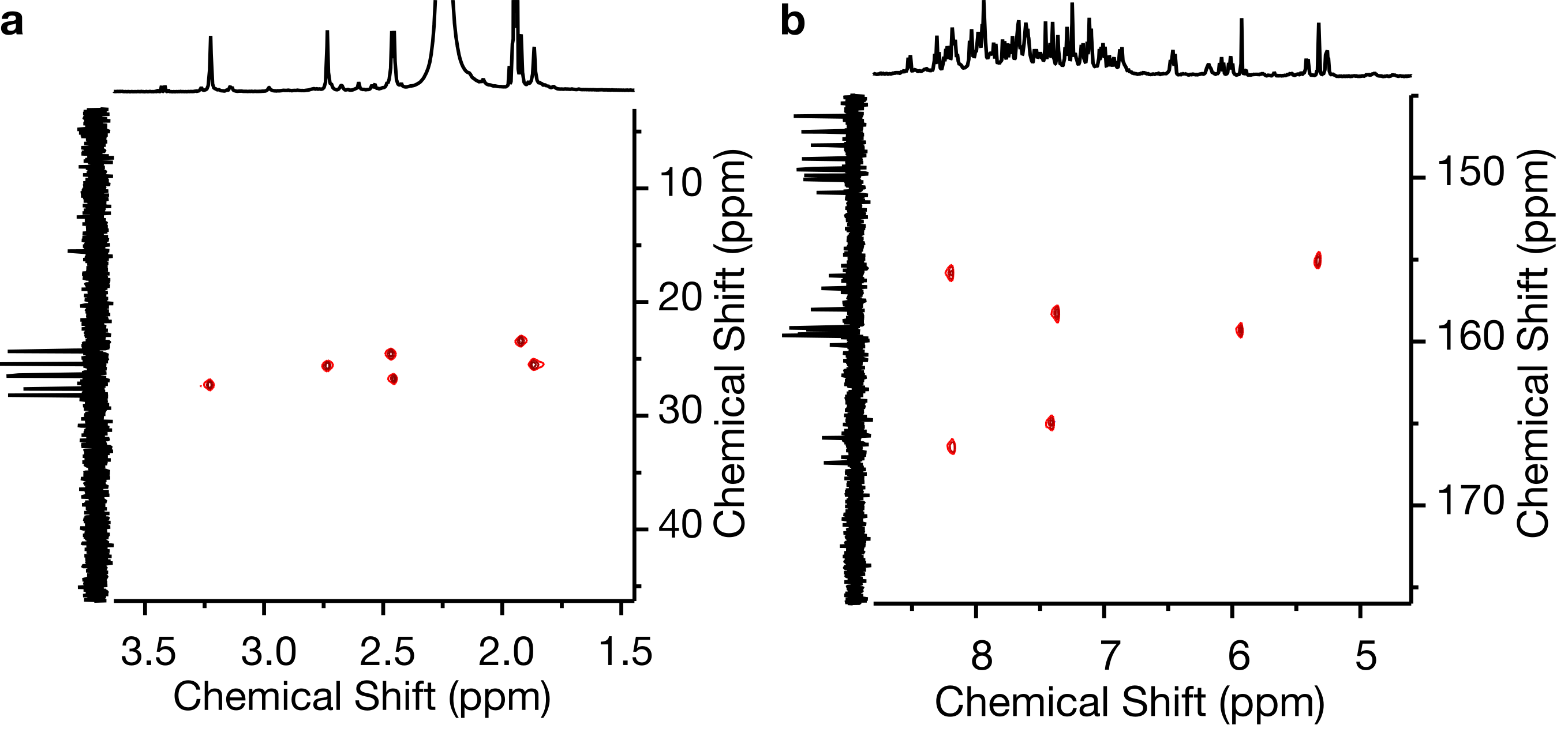


Figure S13. Partial ^1^H-^13^C HSQC spectrum (500 MHz, CD_3_CN, 298 K) of cage 1. a the region showing six methyl signals, b the region showing six imine signals.


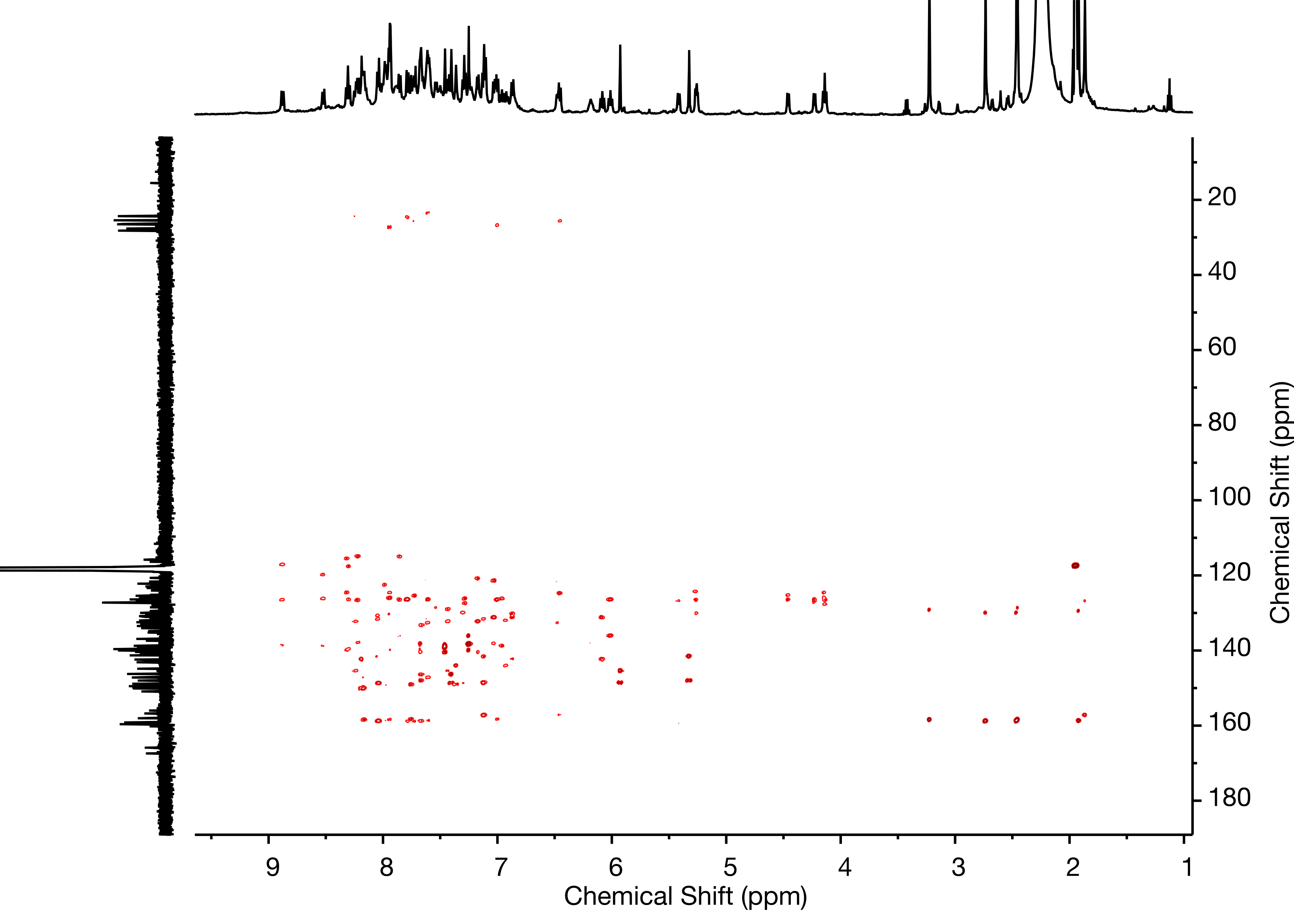


Figure S14. Partial ^1^H-^13^C HMBC spectrum (700 MHz, CD_3_CN, 298 K) of pseudo-hexagonal prismatic cage 1·(BF_4_)_12_. No cross peaks of the cage have been found in other regions.


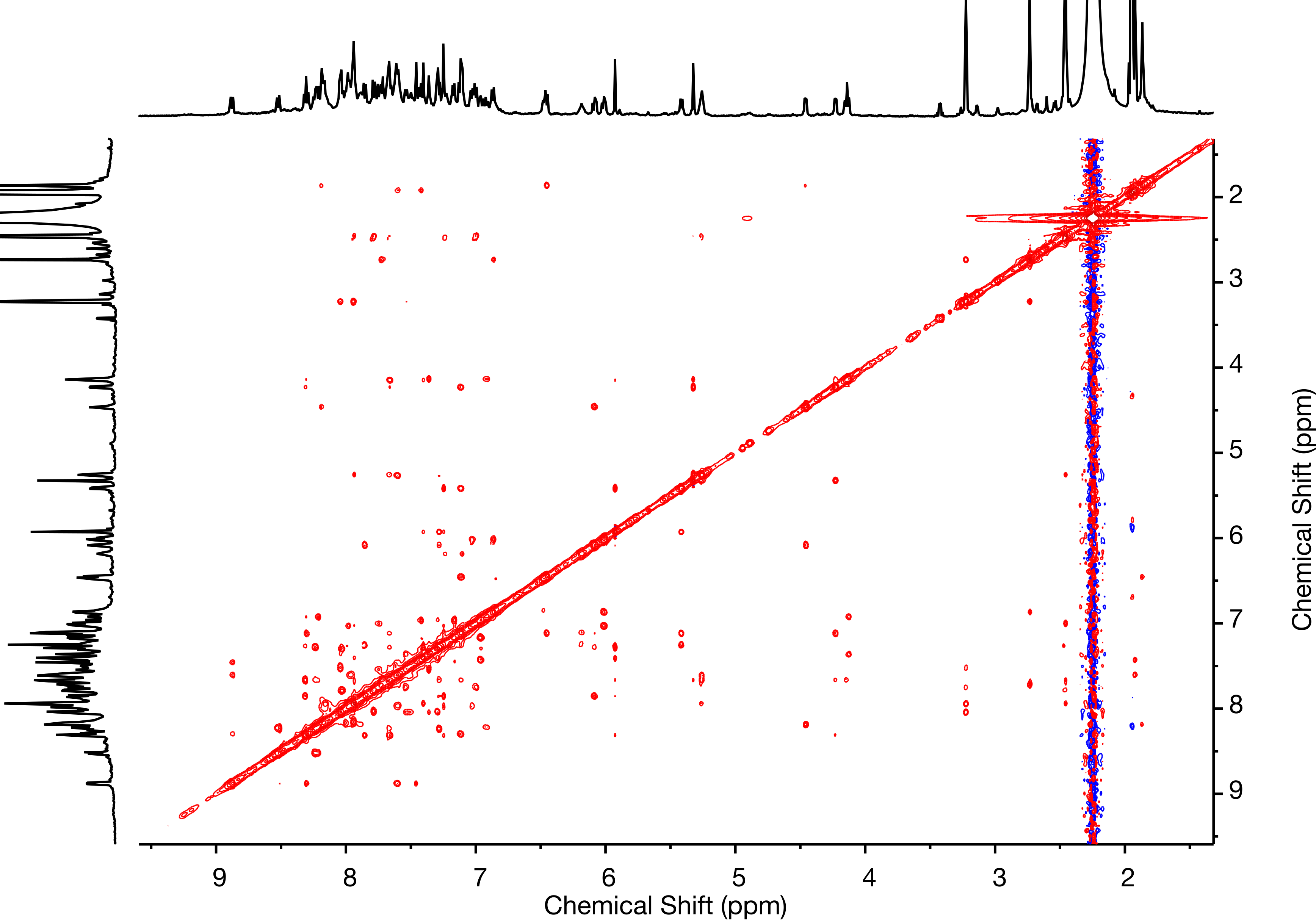


Figure S15. Aromatic region of the ^1^H-^1^H NOESY spectrum (700 MHz, CD_3_CN, 298 K) of pseudo-hexagonal prismatic cage 1·(BF_4_)_12_.


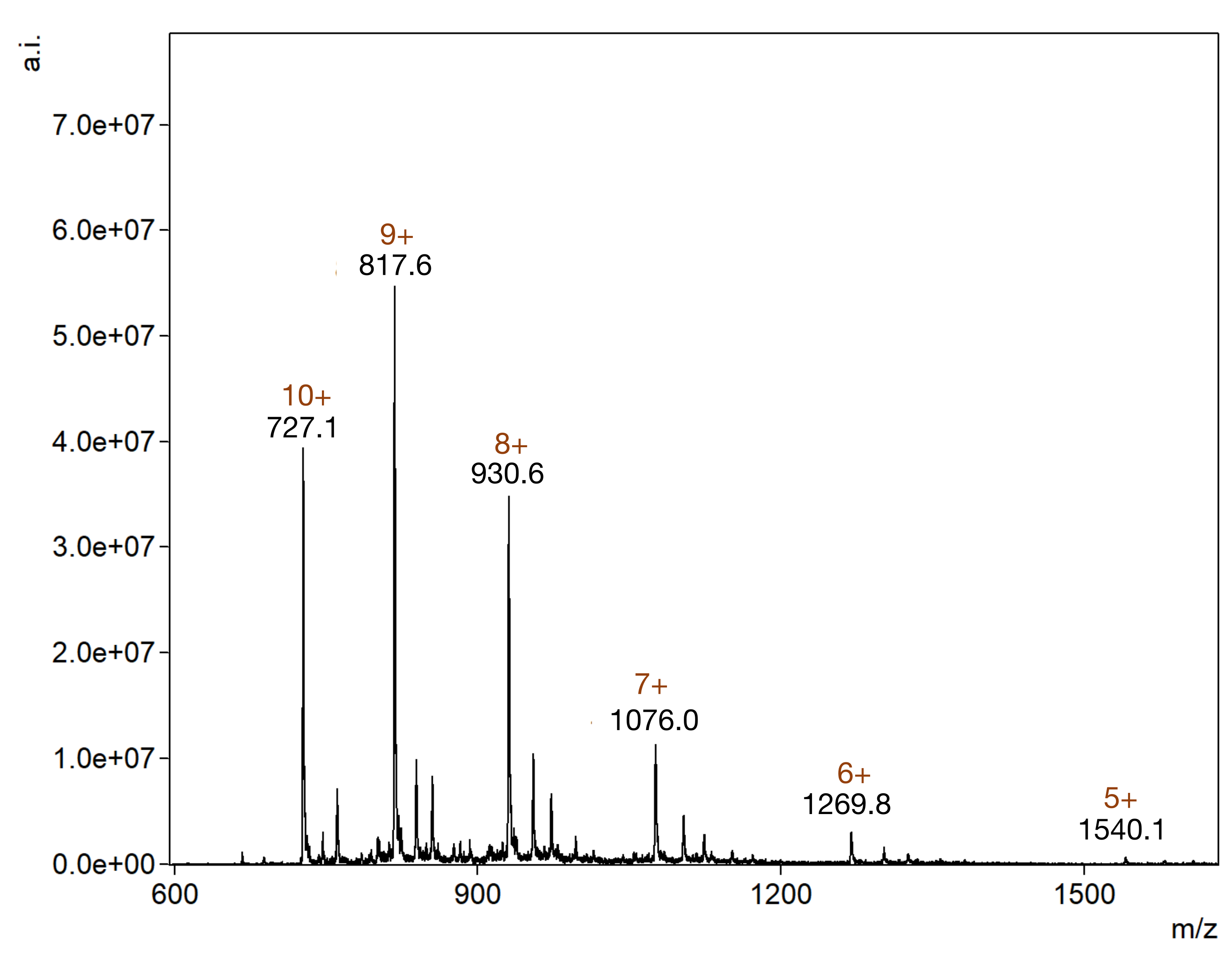


Figure S16. ESI-LRMS spectrum of pseudo-hexagonal prismatic cage 1·(BF_4_)_12_, a Cu_12_L_6_·(BF_4_)_12_ composition of the cage is evident.


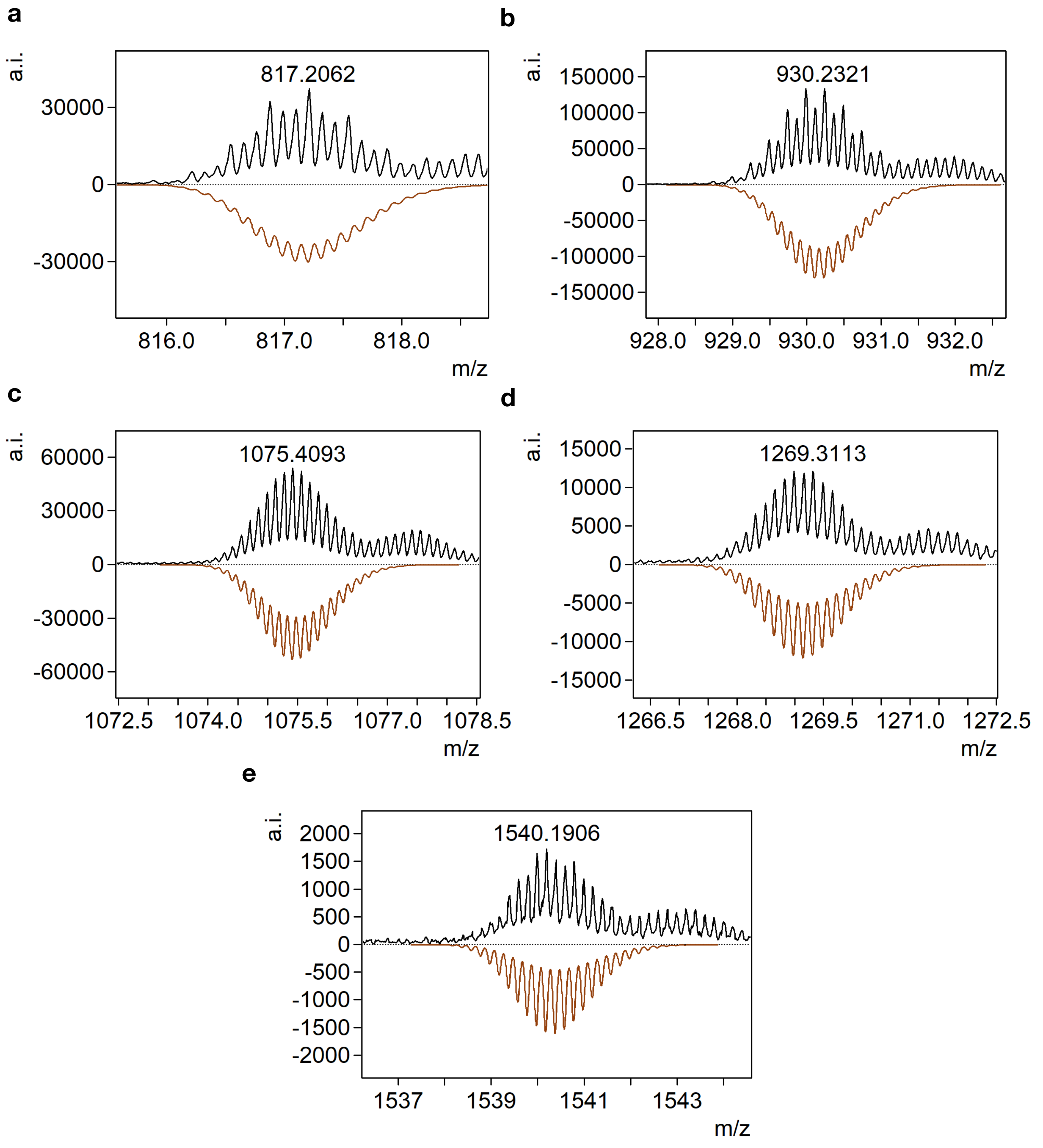


Figure S17. Signals from the high-resolution ESI-mass spectrum for pseudo-hexagonal prismatic cage 1·(BF_4_)_12_. Experimental (black) and calculated (brown) signals for a [1·(BF_4_)_3_]^9+^ b [1·(BF_4_)_4_]^8+^ c [1·(BF_4_)_5_]^7+^ d [1·(BF_4_)_6_]^6+^ e [1·(BF_4_)_7_]^5+^.

The preparation and characterization of **1**·(OTf)_12_ is summarized as below:

Tetramine subcomponent **A** (4.11 mg, 6.39 µmol, 1.0 equiv.) and Cu(CH_3_CN)_4_OTf (4.82 mg, 12.8 µmol, 2.0 equiv.) were mixed in a Schlenk tube before a solution of 6-methyl-2-formylpyridine (**B**) in freshly distilled acetonitrile (60.0 mmol·L^-1^, 0.5 mL, 4.4 equiv.) was added. The mixture was degassed via three freeze-pump-thaw cycles, before being heated at 70°C for 16 hours. The reaction was cooled to room temperature, concentrated to 0.2 mL under a flow of N_2_, and the product precipitated by addition of diethyl ether (15 mL). The mixture was then centrifuged and the supernatant was discarded. The precipitate was washed with diethyl ether (3 × 15 mL), dried *in vacuo*, affording cage **1**·(OTf)_12_ as a dark brown solid (8.68 mg, 0.97 µmol, 92%).

**^1^H NMR** (500 MHz, CD_3_CN, 298 K): δ = 9.18–9.04 (m, 4H), 8.87–8.75 (m, 4H), 8.74–8.64 (m, 4H*), 8.62–8.53 (m, 4H*), 8.35–8.24 (m, 8H), 8.20–8.15 (m, 8H), 8.14–8.10 (m, 4H), 8.09–8.05 (m, 8H), 8.01–7.93 (m, 16H*), 7.92–7.89 (m, 4H)*, 7.88–7.83 (m, 8H), 7.82–7.78 (m, 8H), 7.75–7.64 (m, 20H*), 7.62–7.57 (m, 8H*), 7.56–7.50 (m, 8H*), 7.43–7.35 (m, 3H), 7.31–7.20 (m, 20H*), 7.18–7.13 (m, 8H), 7.12–7.06 (m, 4H*), 7.02–6.90 (m, 16H), 6.78–6.70 (m, 4H), 6.69–6.62 (m, 4H), 6.61–6.55 (m, 4H), 6.54–6.44 (m, 8H), 6.31–6.20 (m, 4H*), 6.09–5.96 (m, 8H), 5.94–5.82 (m, 4H*), 5.74–5.62 (m, 4H*), 5.39–5.18 (m, 8H), 4.25–4.16 (m, 4H), 4.15–4.07 (m, 4H), 4.04–3.87 (m, 4H*), 3.74–3.59 (m, 4H*), 3.32–3.18 (m, 12H), 2.82–2.71 (m, 12H), 2.62–2.47 (m, 24H), 1.93–1.78 (m, 12H). *The relative intensities of these signals in the ^1^H NMR spectrum are slightly higher than expected (and quoted) due to signal overlap.

**ESI-LRMS** [**1**·(OTf)_12_] **=** (C_46_H_34_N_4_(C_7_H_5_N)_4_)_6_Cu_12_(OTf)_12_) *m/z* = 739.7 [**1**·(OTf)_2_]^10+^ (calc. 739.2) 838.4 [**1**·(OTf)_3_]^9+^ (calc. 837.9) 961.9 [**1**·(OTf)_4_]^8+^ (calc. 961.3) 1120.5 [**1**·(OTf)_5_]^7+^ (calc. 1119.9) 1332.2 [**1**·(OTf)_6_]^6+^ (calc. 1331.4) 1628.1 [**1**·(OTf)_7_]^5+^ (calc. 1627.5)


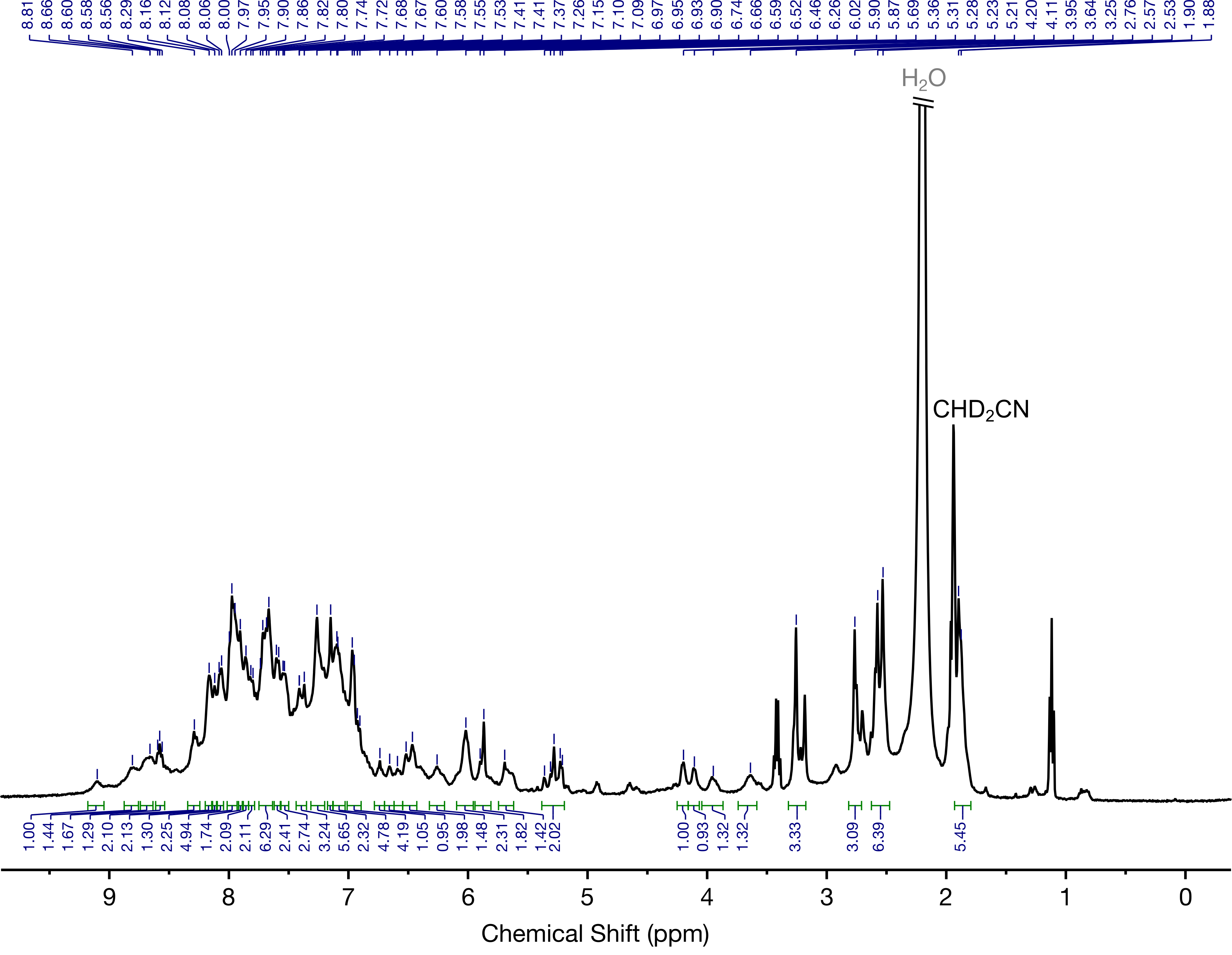


Figure S18. ^1^H NMR Spectrum (400 MHz, CD_3_CN, 298 K) of pseudo-hexagonal prismatic cage 1·(OTf)_12_.


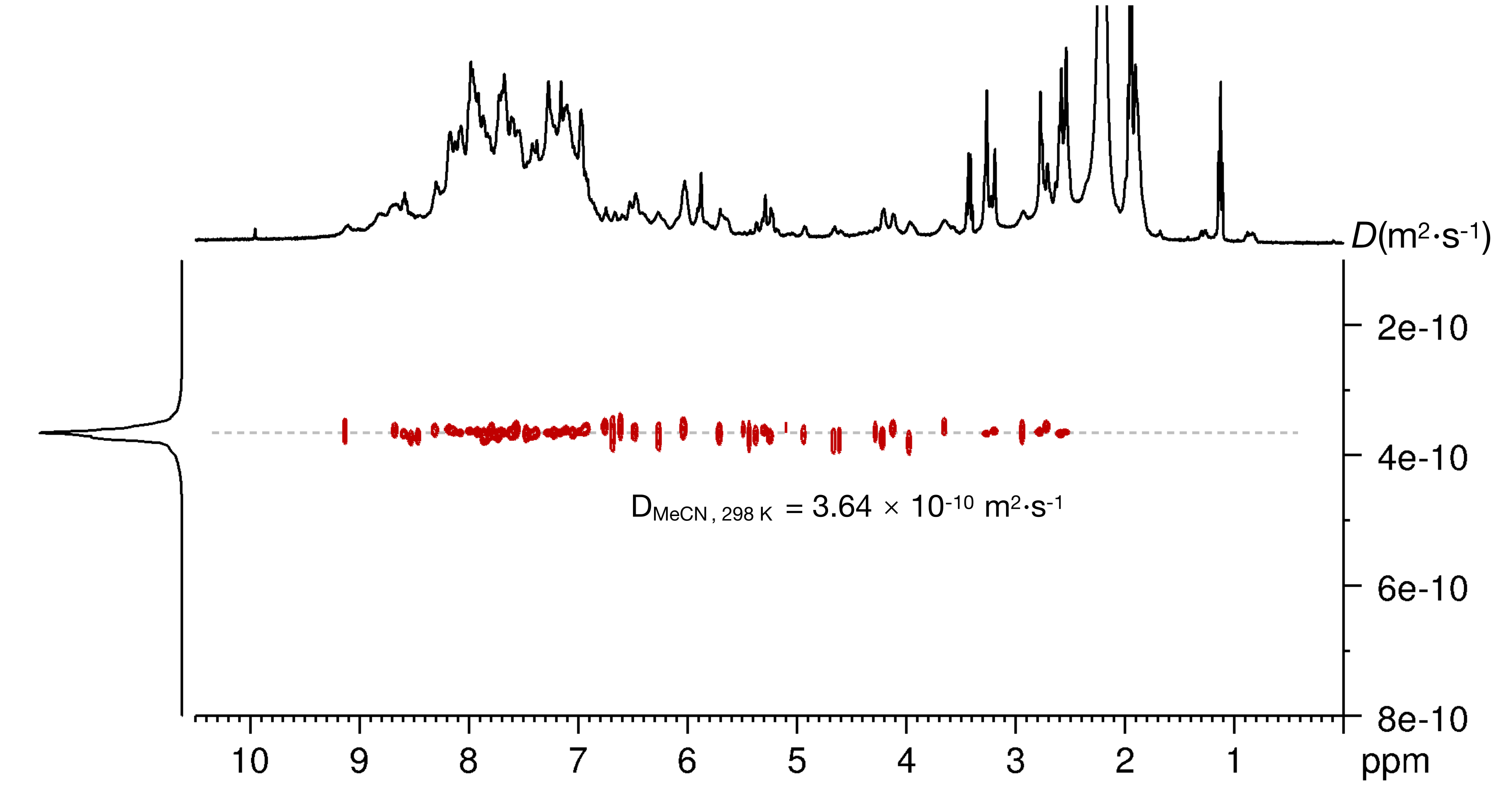


Figure S19. ^1^H-DOSY spectrum (400 MHz, CD_3_CN, 298 K) of pseudo-hexagonal prismatic cage 1·(OTf)_12_. The signals of the cage reveal a diffusion coefficient (*D*) of 3.64 × 10^-10^ m^2^ s^-1^ (400 MHz, 298 K, CD_3_CN).


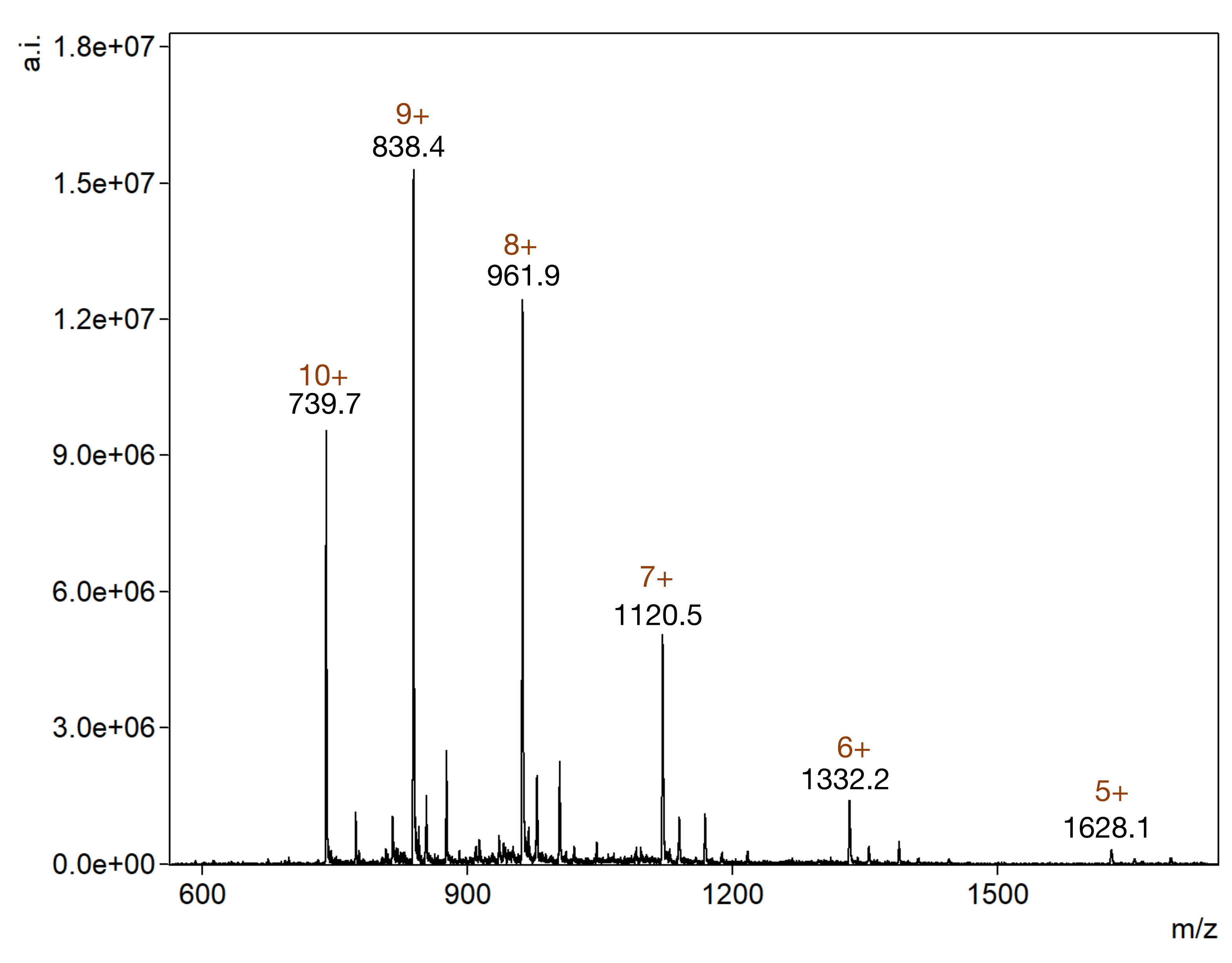


Figure S20. ESI-LRMS spectrum of pseudo-hexagonal prismatic cage 1·(OTf)_12_, a Cu_12_L_6_·(OTf)_12_ composition of the cage is evident.

## 2.3 Rectangular open prism 2


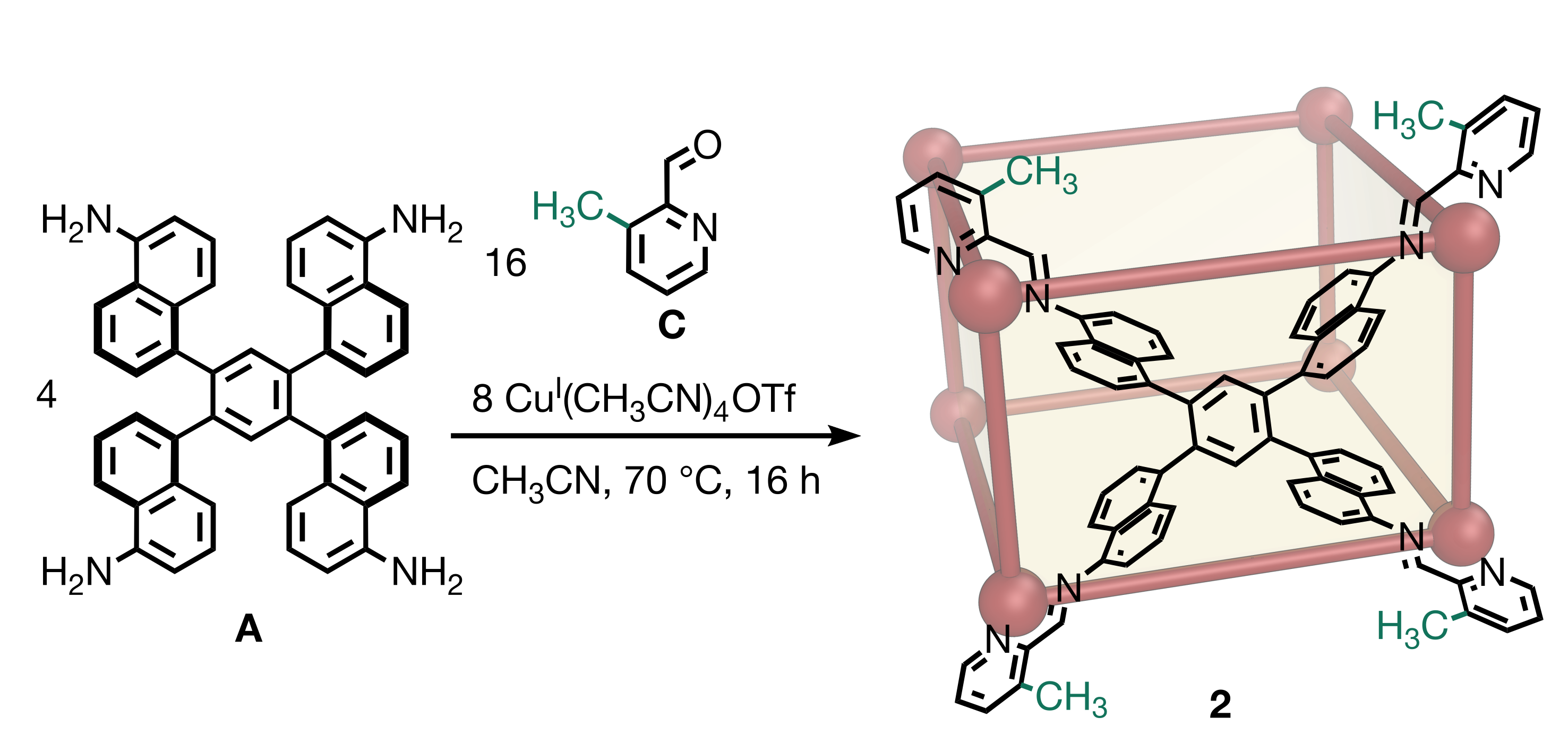


Scheme S3. The self-assembly of rectangular open prism 2.

Tetramine subcomponent **A** (4.11 mg, 6.39 µmol, 1.0 equiv.) and Cu(CH_3_CN)_4_OTf (4.82 mg, 12.8 µmol, 2.0 equiv.) were mixed in a Schlenk tube before a solution of 3-methyl-2-formylpyridine (**C**) in freshly distilled CH_3_CN (60.0 mmol·L^-1^, 0.5 mL, 4.4 equiv.) was added. The mixture was degassed via three freeze-pump-thaw cycles, before being heated at 70°C for 16 hours. The reaction was cooled to room temperature, concentrated to 0.2 mL under a flow of N_2_, and precipitated by addition of diethyl ether (15 mL). The mixture was then centrifuged and the supernatant was discarded. The precipitate was washed with diethyl ether (3 × 15 mL), dried *in vacuo*, affording cage **2**·(OTf)_8_ as a dark brown solid (8.34 mg, 1.60 µmol, 88%).

**^1^H NMR** (500 MHz, CD_3_CN, 298 K): δ = 8.98 (s, 8H), 8.80 (s, 8H), 8.59 (d, 8H*, *J* = 4.4 Hz), 8.49 (d, 8H*, *J* = 8.9 Hz), 8.24 (d, 8H*, *J* = 8.7 Hz), 8.11 (d, 8H*, *J* = 4.1 Hz), 8.02 (d, 8H*, *J* = 8.4 Hz), 7.97 (d, 8H*, *J* = 7.2 Hz), 7.84*–*7.80 (m, 8H*), 7.78 (d, 8H*, *J* = 7.9 Hz), 7.69*–*7.66 (m, 8H*), 7.53*–*7.50 (m, 8H*), 7.49*–*7.47 (m, 8H*), 7.46*–*7.45 (m, 8H*), 7.44*–*7.43 (m, 8H*), 7.40 (s, 8H), 7.34 (d, 8H*, *J* = 7.2 Hz), 7.23 (d, 8H*, *J* = 7.0 Hz), 7.16 (d, 8H, *J* = 6.9 Hz), 6.89*–*6.85 (m, 8H*), 6.79*–*6.75 (m, 8H), 5.31*–*5.26 (m, 8H), 2.62 (s, 24H*), 2.51 (s, 24H). Note that only the signals for the predominant species identified as cage **2**·(OTf)_8_ have been listed. *The relative intensities of these signals in the ^1^H NMR spectrum are slightly higher than expected (and quoted) due to signal overlap.

**^13^C NMR** (126 MHz, CD_3_CN, 298 K): δ 161.9, 161.8, 149.2, 147.6, 147.3, 147.2, 141.8, 141.5, 140.5, 140.4, 139.8, 139.3, 134.2, 133.6, 133.5, 133.1, 129.9, 129.4, 128.7, 128.5, 128.3, 128.2, 127.8, 127.6, 127.5, 127.3, 127.2, 127.0, 126.6, 126.3, 126.2, 126.0, 122.6, 122.2 (q, *J* = 321 Hz, OTf^-^), 121.5, 120.6, 17.6, 17.4.

**ESI-HRMS** [**2**·(OTf)_8_] **=** (C_46_H_34_N_4_(C_7_H_5_N)_4_)_4_Cu_8_(OTf)_8_) *m/z* = 696.8822 [**2**·(OTf)]^7+^ (calc. 696.9343) 837.8544 [**2**·(OTf)_2_]^6+^ (calc. 837.9352) 1035.2185 [**2**·(OTf)_3_]^5+^ (calc. 1035.3364) 1331.2690 [**2**·(OTf)_4_]^4+^ (calc. 1331.4382)


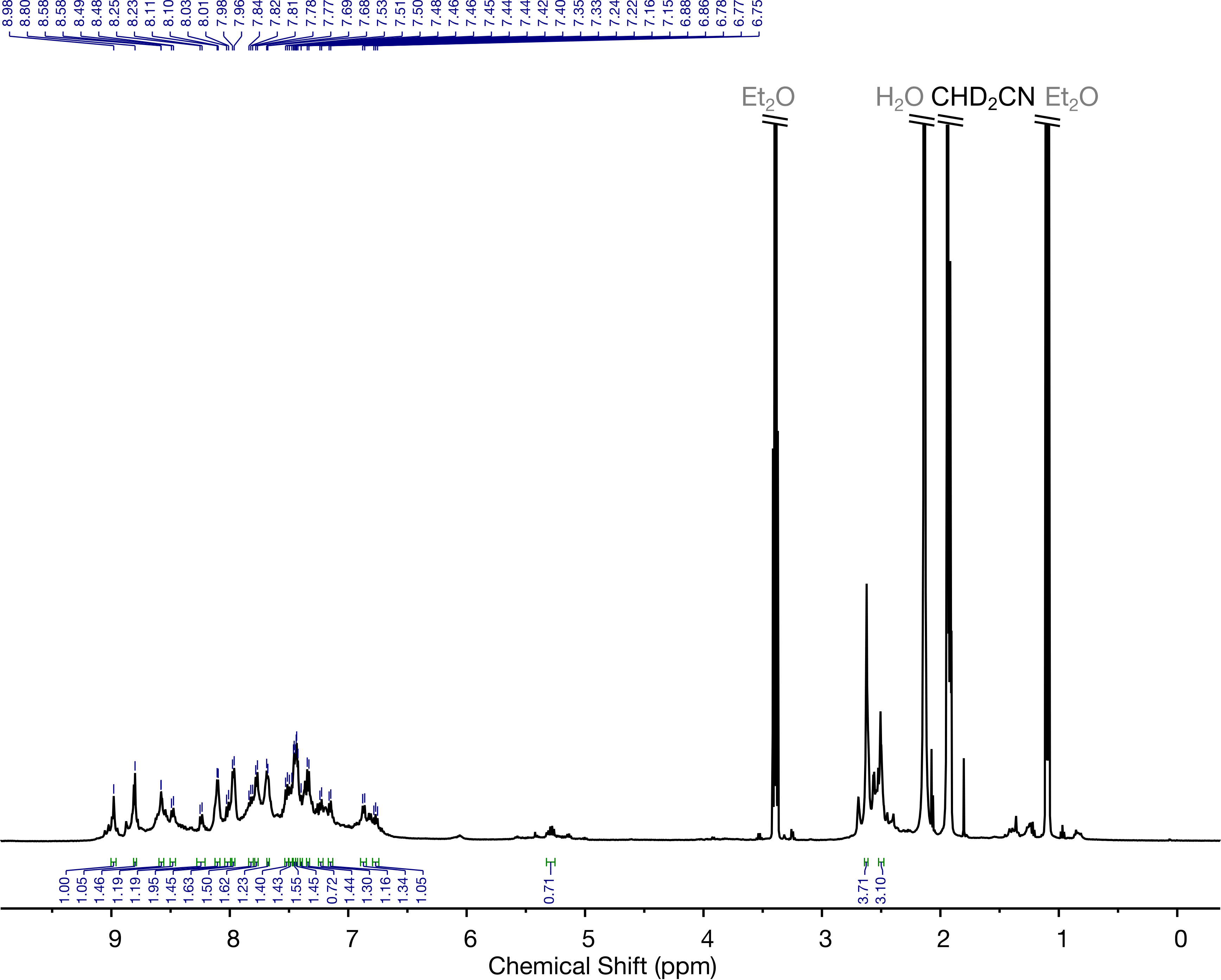


Figure S21. ^1^H NMR Spectrum (500 MHz, CD_3_CN, 298 K) of rectangular open prism 2·(OTf)_8_. Note that only the predominant set of signals corresponding to cage 2·(OTf)_8_ has been labelled.

The ¹H NMR signals of cage 2·(OTf)_12_ are assigned and visualized in Figure S22, with detailed assignments listed in Table S2:


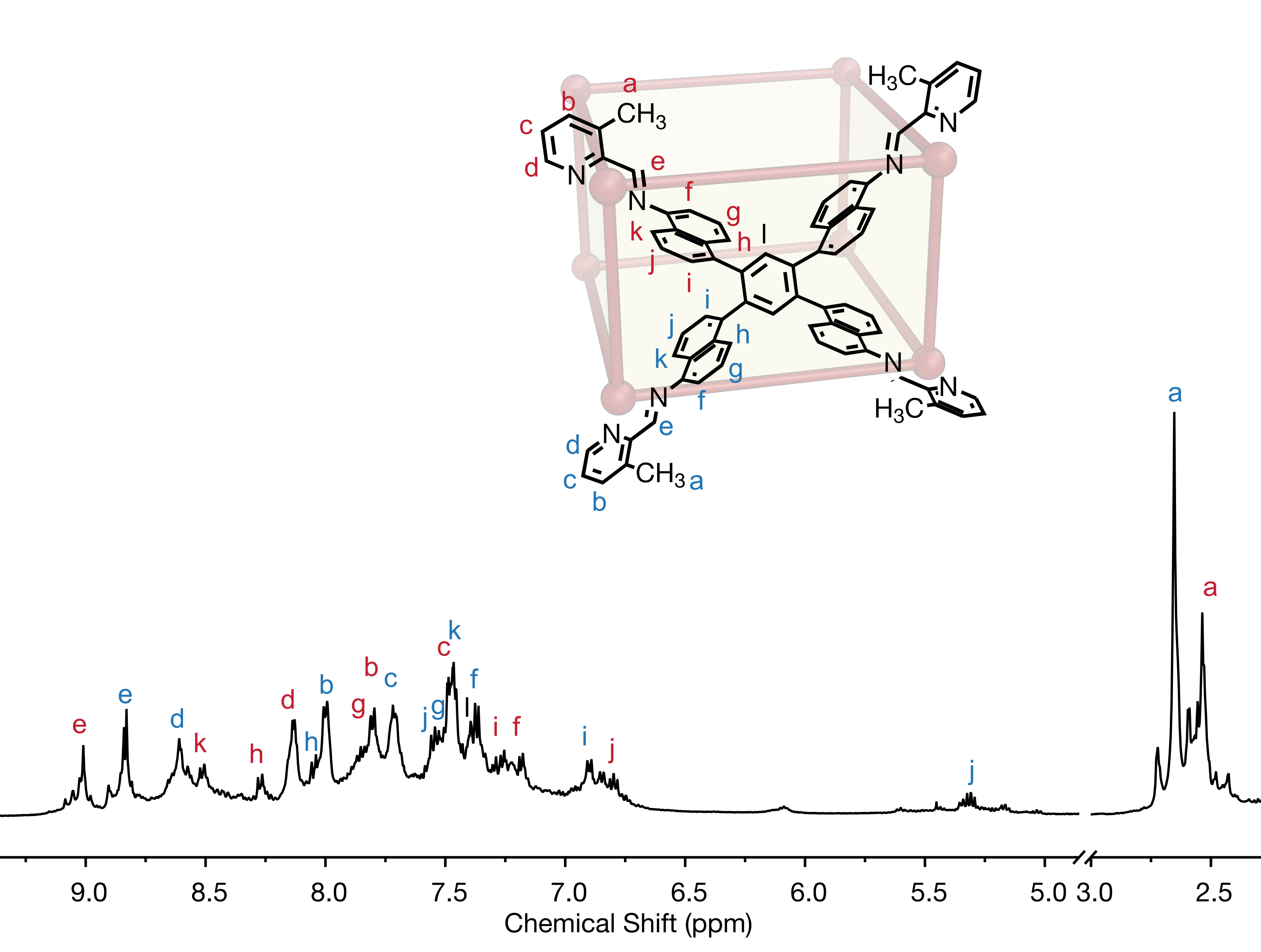


Figure S22. ^1^H NMR Spectrum (500 MHz, CD_3_CN, 298 K) of rectangular open prism 2·(OTf)_8_, with assignment of signals. Multiple species appear to be present, with the main set of signals identified and assigned as cage 2·(OTf)_8_. The signals of protons in each distinct ligand arm could be distinguished and are marked with different colours. These signals are attributed to particular ligand arms within the structure as shown in the figure, based on the SCXRD structure which revealed that proton h in the blue-denoted ligand arm is strongly shielded by another naphthylene, whereas the corresponding proton in the red ligand arm is not.

**Table S2.** ^1^H NMR signal assignments for **2**·(OTf)_8_ (500 MHz, CD₃CN, 298 K). Only signals for the predominant species identified as cage **2**·(OTf)_8_ have been listed. Two distinct ligand arm environments are present, denoted red and blue as in Figure S21. The blue-labelled arm has proton *h* strongly shielded by an adjacent naphthylene, while the corresponding proton in the red arm is not. Signals marked with * have slightly higher relative intensities than expected due to signal overlap.

| **Chemical Shift (δ, ppm)** | **Multiplicity** | **No. of H** | ***J* (Hz)** | **Assignment** |
| --- | --- | --- | --- | --- |
| 8.98 | s | 8H |  | e (imine) |
| 8.80 | s | 8H |  | e (imine) |
| 8.59 | d | 8H* | 4.4 | d |
| 8.49 | d | 8H* | 8.9 | k |
| 8.24 | d | 8H* | 8.7 | h |
| 8.11 | d | 8H* | 4.1 | d |
| 8.02 | d | 8H* | 8.4 | h |
| 7.97 | d | 8H* | 7.2 | b |
| 7.84–7.80 | m | 8H* |  | g |
| 7.78 | d | 8H* | 7.9 | b |
| 7.69–7.66 | m | 8H* |  | c |
| 7.53–7.50 | m | 8H* |  | j |
| 7.49–7.47 | m | 8H* |  | g |
| 7.46–7.45 | m | 8H* |  | c |
| 7.44–7.43 | m | 8H* |  | k |
| 7.40 | s | 8H |  | l |
| 7.34 | d | 8H* | 7.2 | f |
| 7.23 | d | 8H* | 7.0 | i |
| 7.16 | d | 8H | 6.9 | f |
| 6.89–6.85 | m | 8H* |  | i |
| 6.79–6.75 | m | 8H |  | j |
| 5.31–5.26 | m | 8H |  | j |
| 2.62 | s | 24H* |  | a (CH_3_) |
| 2.51 | s | 24H |  | a (CH_3_) |

* These protons have slightly higher relative intensities than expected due to signal overlap.


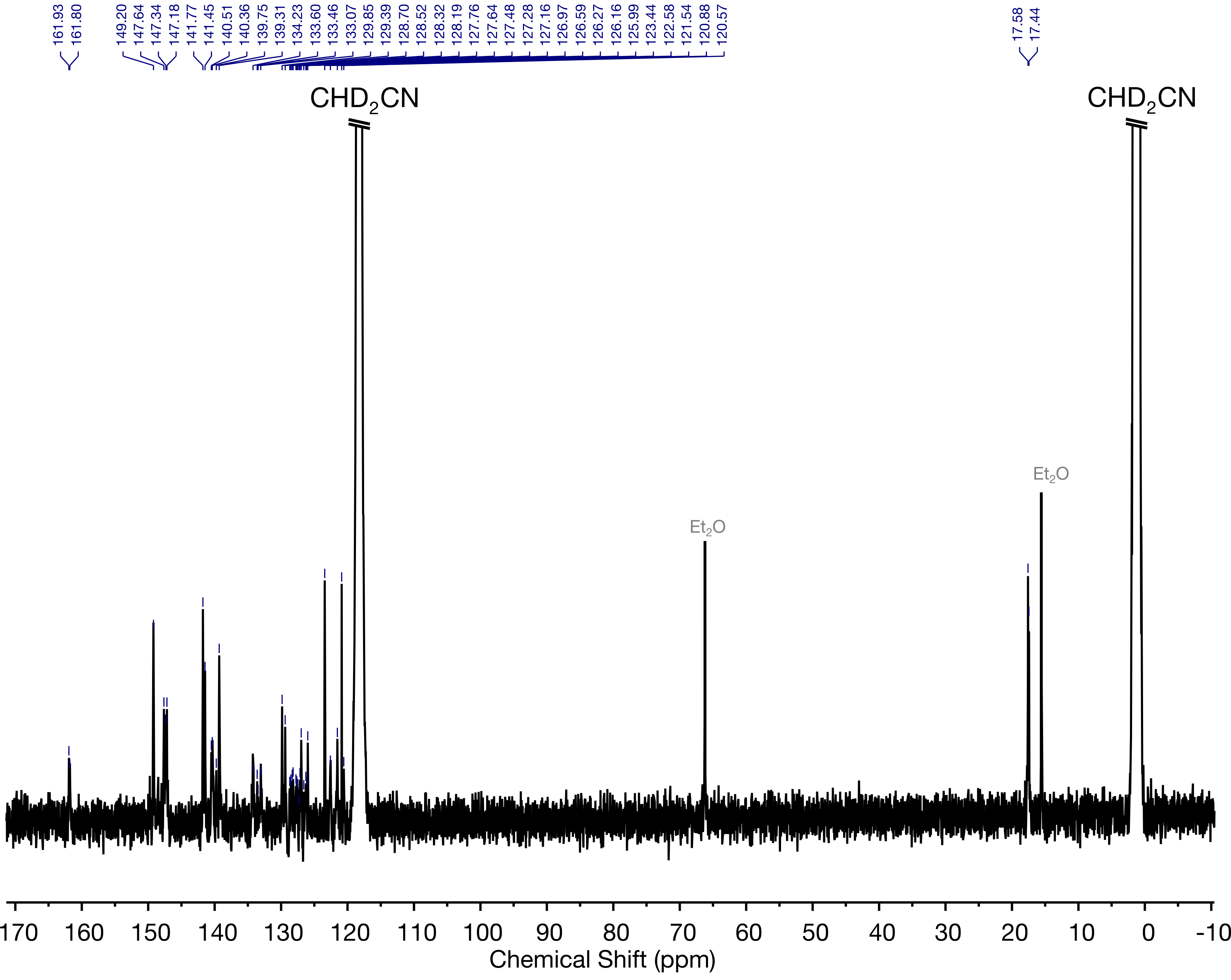


Figure S23. ^13^C NMR Spectrum (126 MHz, CD_3_CN, 298 K) of rectangular open prism 2·(OTf)_8_.


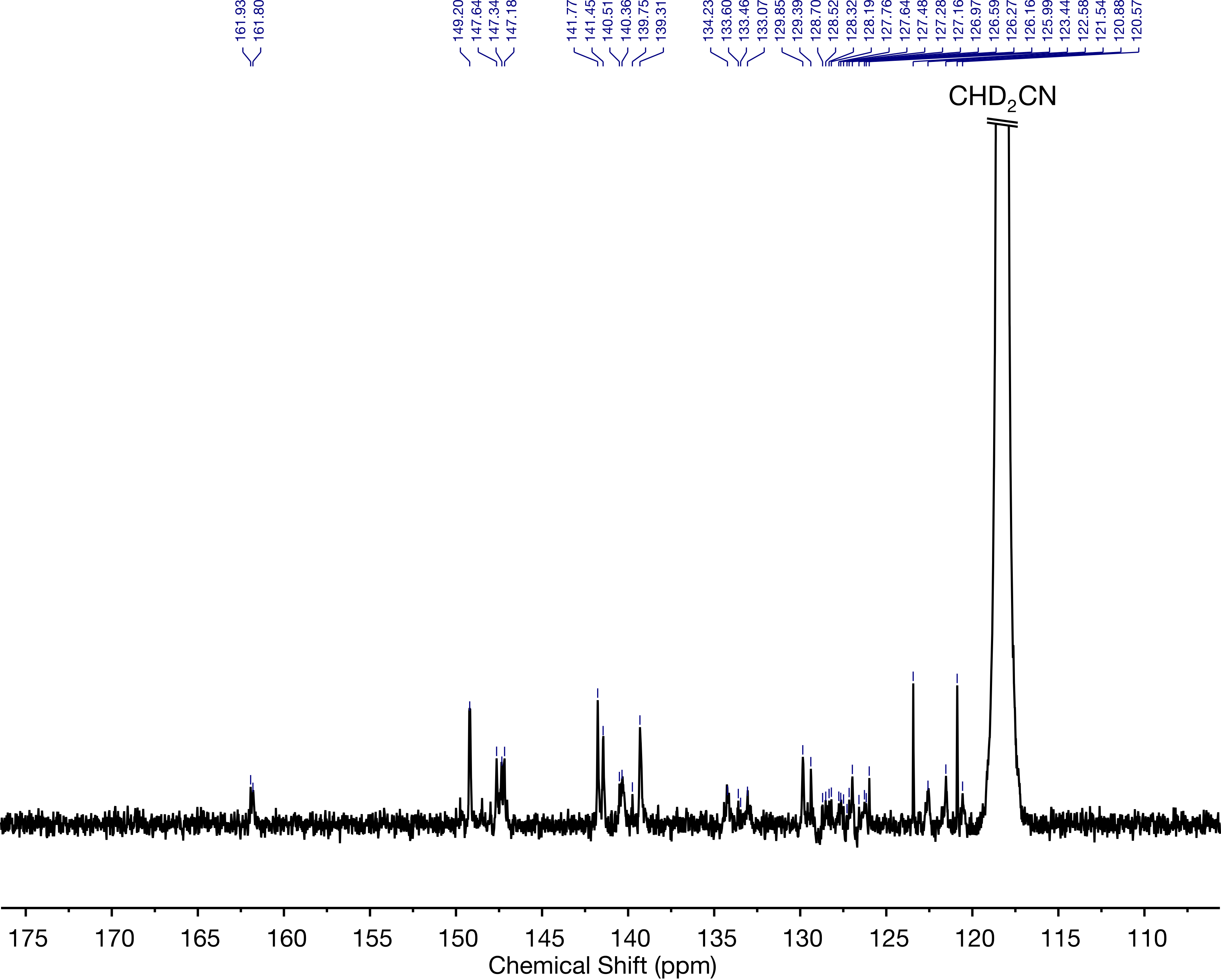


Figure S24. Aromatic region of the ^13^C NMR Spectrum (126 MHz, CD_3_CN, 298 K) of rectangular open prism 2·(OTf)_8_.


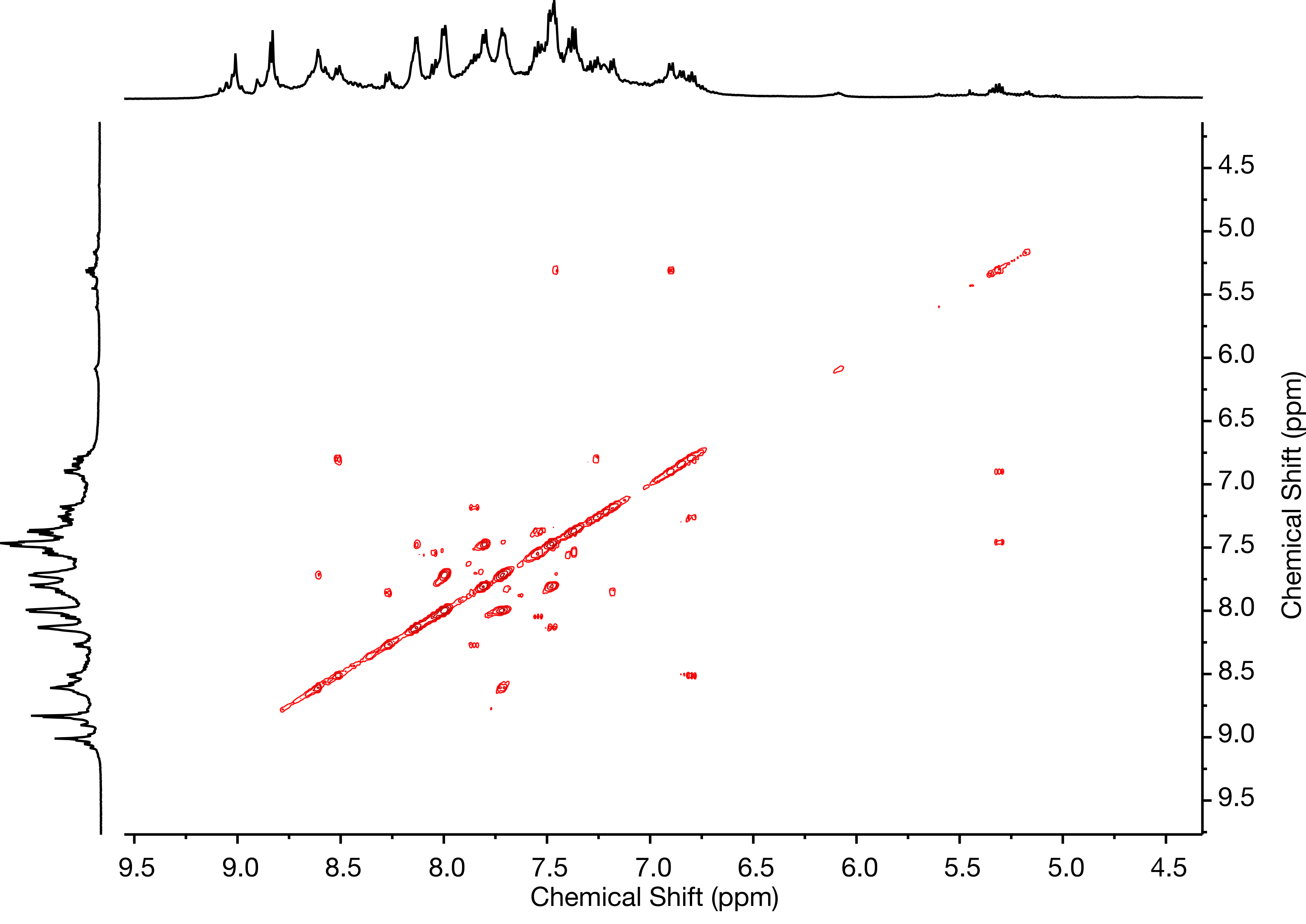


Figure S25. Aromatic region of the ^1^H-^1^H DQF-COSY spectrum (500 MHz, CD_3_CN, 298 K) of rectangular open prism 2·(OTf)_8_.


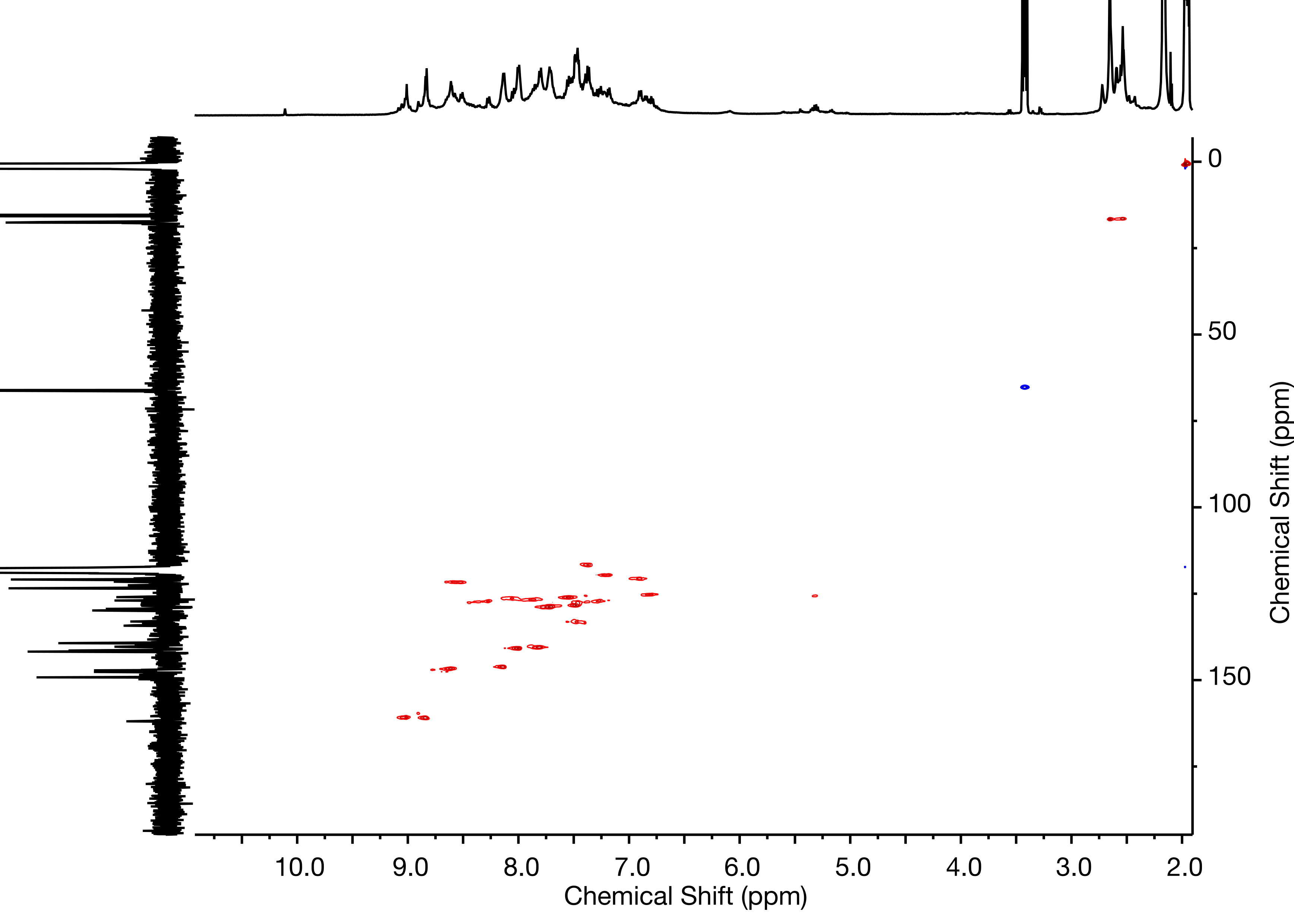


Figure S26. The ^1^H-^13^C HSQC spectrum (500 MHz, CD_3_CN, 298 K) of rectangular open prism 2·(OTf)_8_.


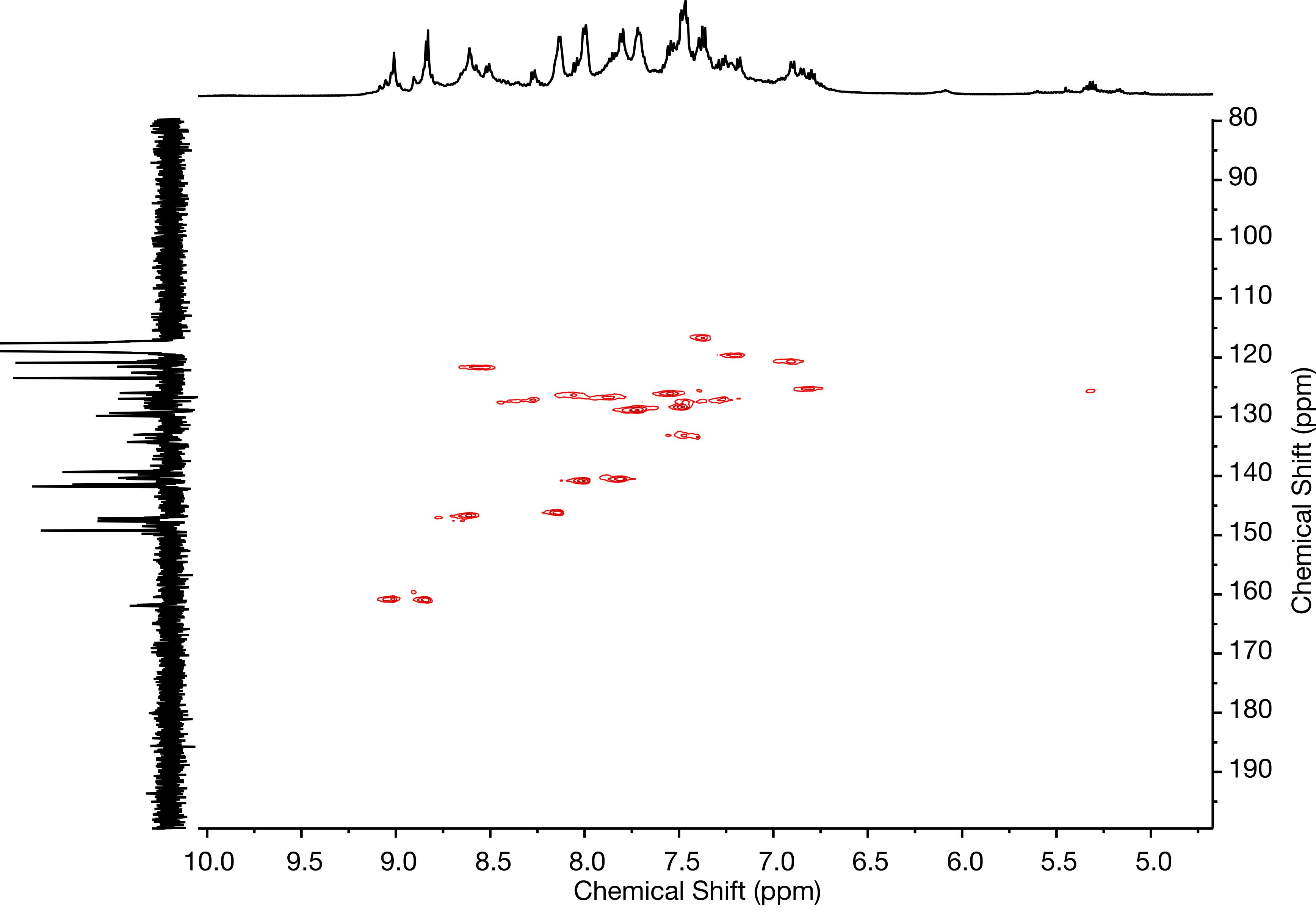


Figure S27. Aromatic region of the ^1^H-^13^C HSQC spectrum (500 MHz, CD_3_CN, 298 K) of rectangular open prism 2·(OTf)_8_.


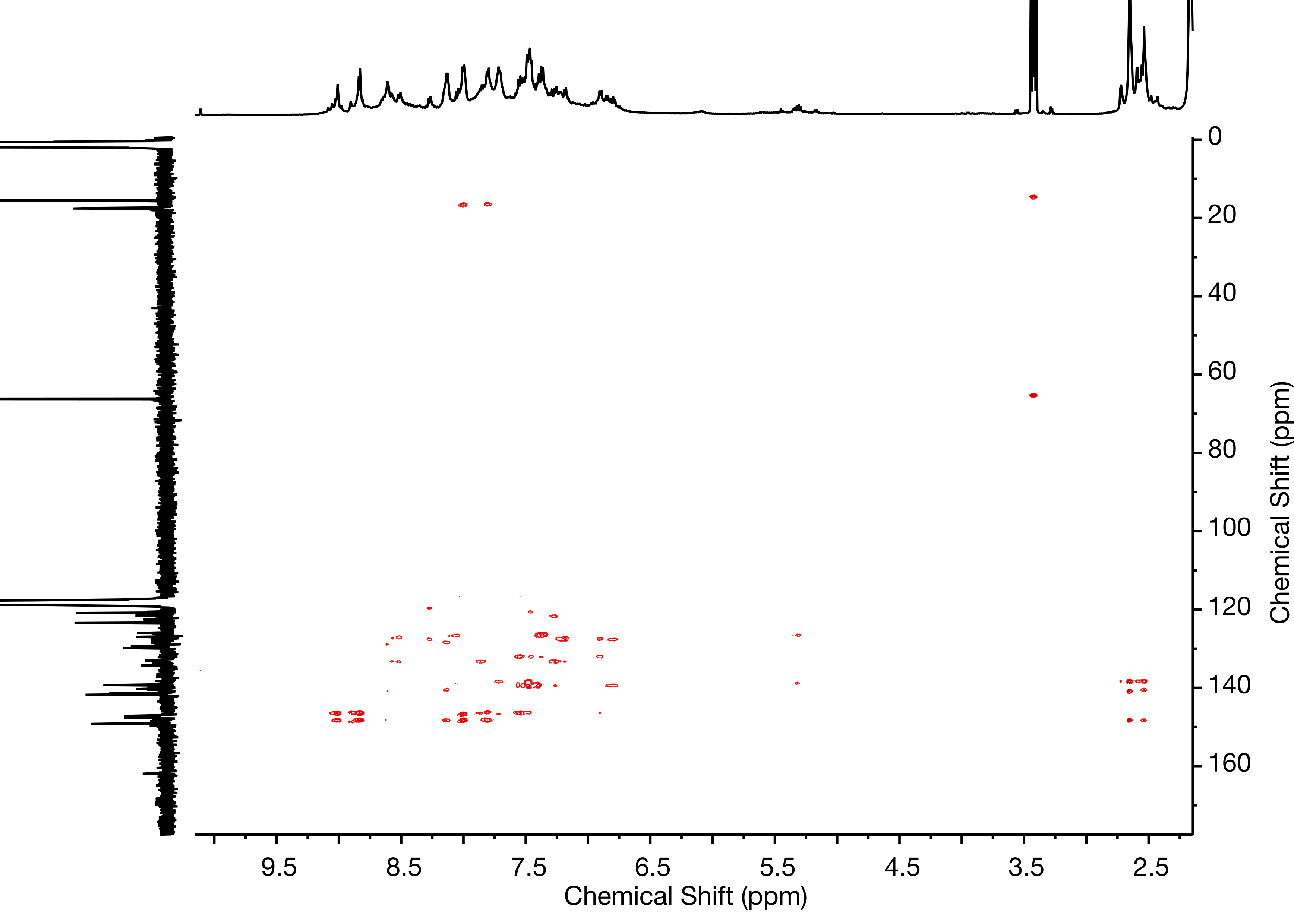


Figure S28. Partial ^1^H-^13^C HMBC spectrum (500 MHz, CD_3_CN, 298 K) of rectangular open prism 2·(OTf)_8_. No cross peaks of the cage have been found in other regions.


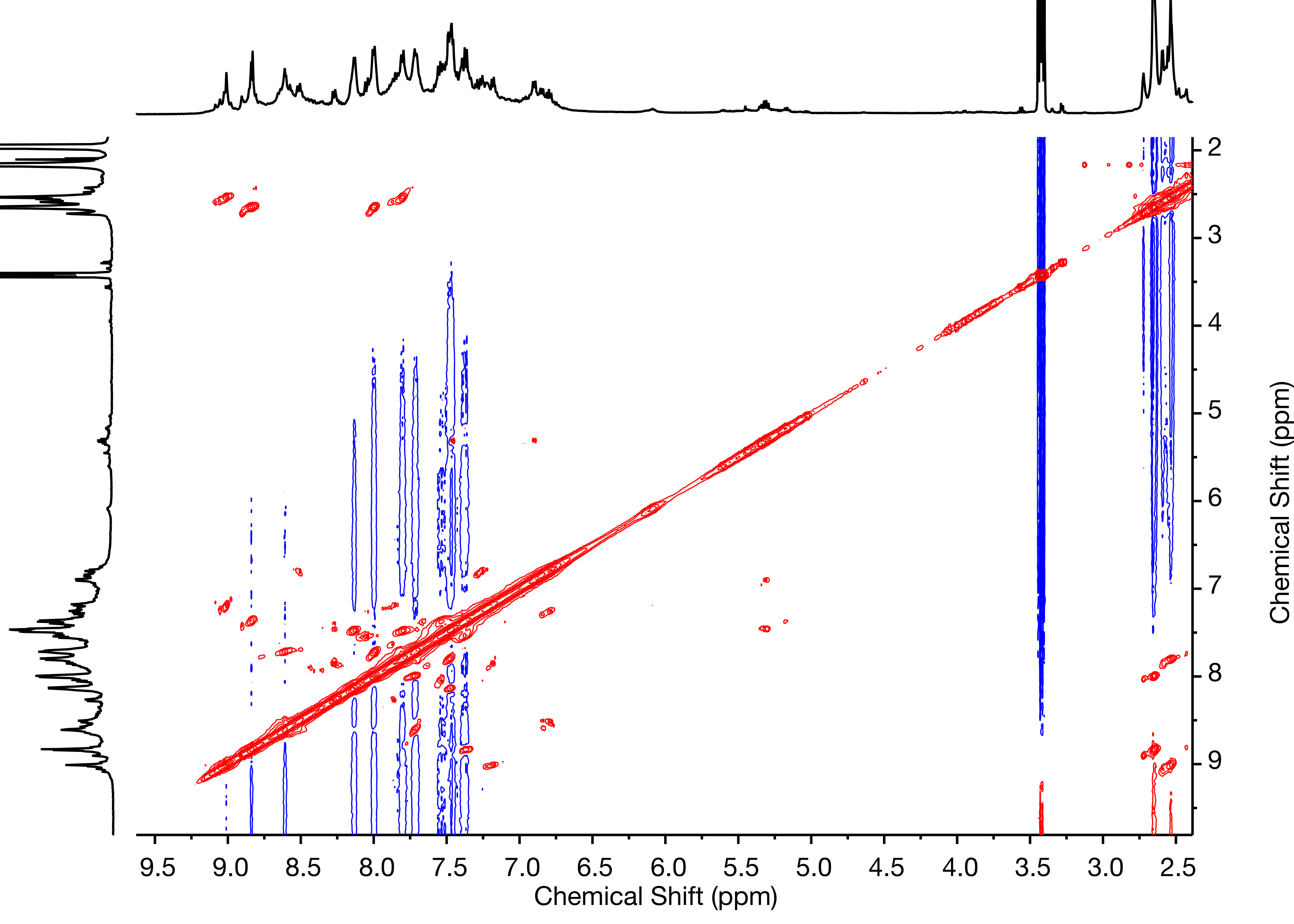


Figure S29. The ^1^H-^1^H NOESY spectrum (500 MHz, CD_3_CN, 298 K) of rectangular open prism 2·(OTf)_8_.


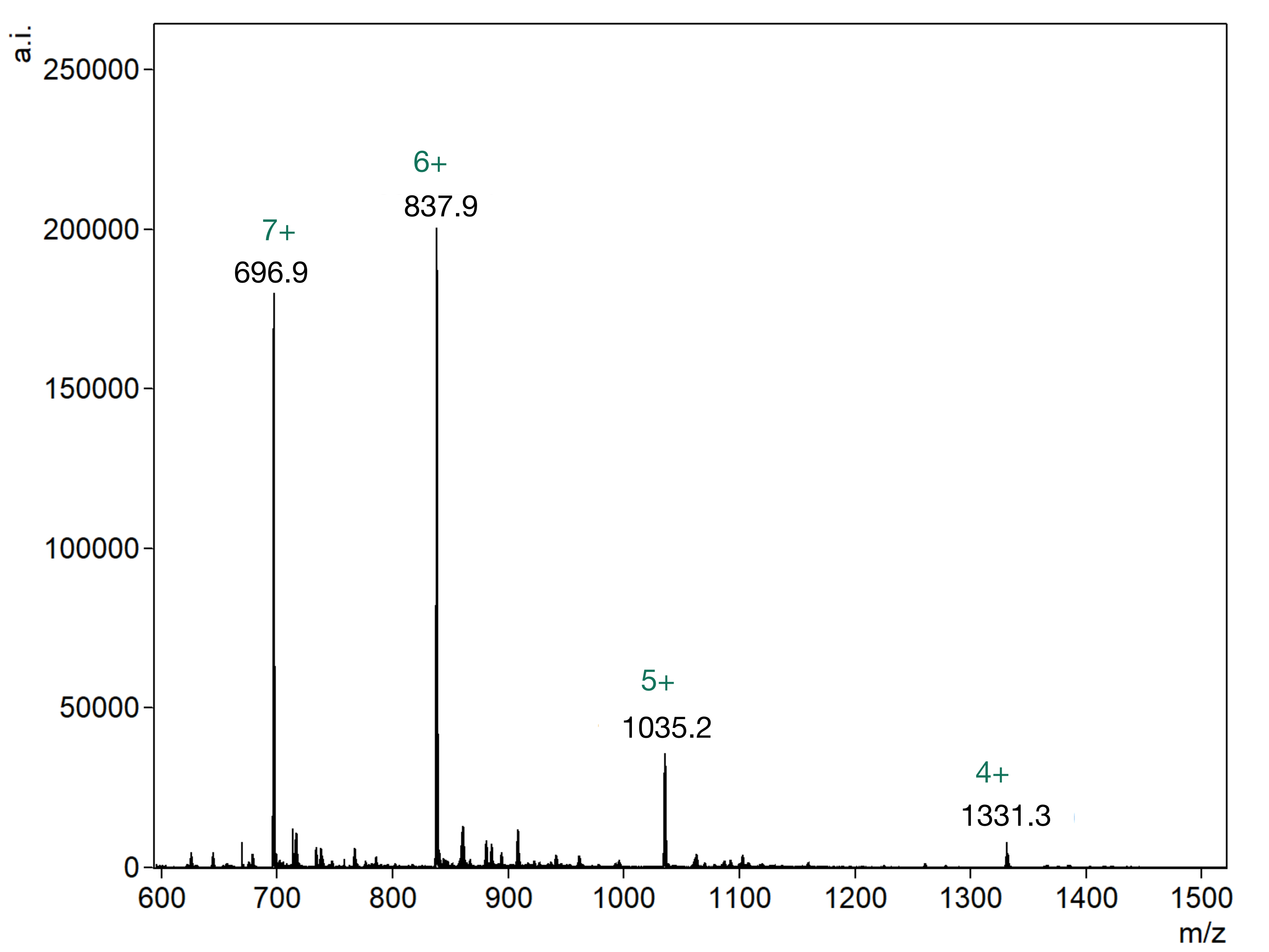


Figure S30. ESI-HRMS spectrum of rectangular open prism 2·(OTf)_8_, a Cu_8_L_4_·(OTf)_8_ composition of the cage is evident.


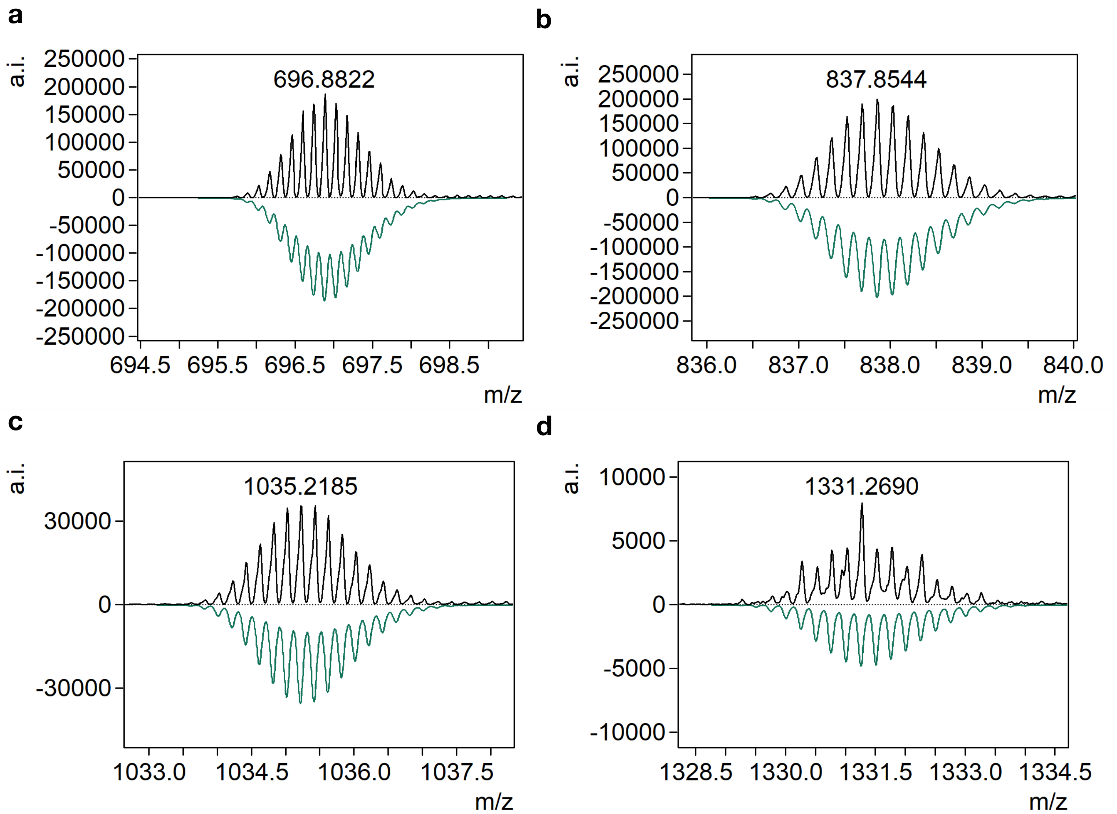


Figure S31. Signals from the high-resolution ESI-mass spectrum for 2·(OTf)_8_. Experimental (black) and calculated (blue) signals for a [2·(OTf)]^7+^ b [2·(OTf)_2_]^6+^ c [2·(OTf)_3_]^5+^ d [2·(OTf)_4_]^4+^.

# 3 X-ray crystallography

Data were collected at Beamline I19 of Diamond Light Source employing silicon double crystal monochromated synchrotron radiation (0.6889 Å) with ω and ψ scans at 100(2) K.^[2]^ Data integration and reduction were undertaken with Xia2.^[3]^ Subsequent computations were carried out using the Olex2 graphical user interface. Multi-scan empirical absorption corrections were applied to the data using the Dials software package.^[3c, 4]^ The structures were solved by direct methods using SHELXT^[5]^ then refined and extended with SHELXL.^[6]^ In general, non-hydrogen atoms with occupancies greater than 0.5 were refined anisotropically. Carbon-bound hydrogen atoms were included in idealized positions and refined using a riding model. Disorder was modelled using standard crystallographic methods including constraints, restraints, and rigid bodies where necessary. Crystallographic data along with specific details pertaining to the refinement follow. Crystallographic data have been deposited in the CCDC database (2499903-2499904, and 2531235).

Table S3. Crystal Data, Solution and Refinement Parameters

|  | **1**·(BF_4_)_12_ | **1**·(OTf)_12_ | **2** |
| --- | --- | --- | --- |
| CCDC number | 2499903 | 2531235 | 2499904 |
| Formula | C_490.47_H_413.96_N_61.10_  Cu_12_B_2_F_48_O_5.07_ | C_489_H_324_Cu_12_F_36_  N_58.10_O_42.40_S_12_ | C_296_H_242.70_Cu_8_F_24_  N_40.90_O_24_S_8_ |
| Formula weight | 9049.33 | 9579.39 | 6287.25 |
| Temperature/K | 100(2) | 100(2) | 100(2) |
| Crystal system | monoclinic | triclinic | tetragonal |
| Space group | P2/n | P-1 | P4_1_2_1_2 |
| *a*/Å | 25.0173(3) | 22.5909(2) | 25.7697(7) |
| *b*/Å | 24.3329(3) | 27.2506(2) | 25.7697(7) |
| *c*/Å | 44.8159(5) | 40.8453(3) | 63.036(3) |
| *α*/° | 90 | 100.5920(10) | 90 |
| *β*/° | 97.356(1) | 96.3970(10) | 90 |
| *γ*/° | 90 | 103.4240(10) | 90 |
| Volume/Å^3^ | 27056.8(6) | 23727.0(3) | 41861(3) |
| Z | 4 | 2 | 4 |
| *ρ*_calc_ g/cm^3^ | 1.068 | 1.341 | 0.998 |
| μ/mm^‑1^ | 0.486 | 0.613 | 0.466 |
| F(000) | 8981.0 | 9848.0 | 12911.0 |
| Crystal size/mm^3^ | 0.30 × 0.30 × 0.30 | 0.10 × 0.10 × 0.10 | 0.10 × 0.10 × 0.10 |
| Radiation | Synchrotron  (λ = 0.6889) | Synchrotron  (λ = 0.6889) | Synchrotron  (λ = 0.6889) |
| 2*θ* range for data collection/° | 1.622 to 47.812 | 0.996 to 45.004 | 2.424 to 33.36 |
| Index ranges | -29 ≤ h ≤ 29, -26 ≤ k ≤ 28, -44 ≤ l ≤ 52 | -23 ≤ h ≤ 25, -30 ≤ k ≤ 30, -42 ≤ l ≤ 45 | -21 ≤ h ≤ 21, -21 ≤ k ≤ 21, -52 ≤ l ≤ 52 |
| Reflections collected | 259493 | 212280 | 84611 |
| Independent reflections | 45982 [R_int_ = 0.0572, R_sigma_ = 0.1041] | 67305 [R_int_ = 0.0568, R_sigma_ = 0.0797] | 12688 [R_int_ = 0.0892, R_sigma_ = 0.1107] |
| Goodness-of-fit on F^2^ | 1.001 | 1.109 | 1.114 |
| Final R indexes [I>=2σ (I)] | R_1_^[a]^= 0.1166,  *w*R_2_^[b]^ = 0.3264 | R_1_^[a]^= 0.1192,  *w*R_2_^[b]^ = 0.3358 | R_1_^[a]^= 0.1426,  *w*R_2_^[b]^ = 0.3501 |
| Final R indexes [all data] | R_1_^[a]^ = 0.1631,  *w*R_2_^[b]^ = 0.3435 | R_1_^[a]^ = 0.1668,  *w*R_2_^[b]^ = 0.3565 | R_1_^[a]^ = 0.1944,  *w*R_2_^[b]^ = 0.3768 |

[a] R_1_ = Σ||*F_o_*| - |*F_c_*|| /Σ |*F_o_*|;

[b] R_2_*w* = [Σ*w*(*F_o_*^2^ - *F_c_*^2^)^2^ / *w*(*F_o_*^2^)^2^]]^1/2^, where *w* = q[*σ^2^(Fo^2^) + (aP)^2^ + bP*]^-1^.

Specific refinement details of **1**·(BF_4_)_12_:

The crystals of **1**·(CH_3_CN)_13.10_·(C_4_H_10_O)_5.07_·(BF_4_)_12_ were grown by diffusion of diethyl ether into an acetonitrile solution of **1**·(BF_4_)_12_. The crystals employed immediately lost solvent after removal from the mother liquor but rapid handling prior to flash cooling in liquid nitrogen and the use of synchrotron radiation enabled data to be collected to approximately 0.85 Å resolution. The asymmetric unit was found to contain a Cu_6_L_3_ moiety, which comprises one half of a Cu_12_L_6_ assembly and associated counterions and solvent molecules.

In order to obtain a reasonable model for the organic parts of the structure, DFIX restraints were applied to parts of the structure displaying a higher degree of thermal motion. Due to the thermal motion, thermal parameter restraints (SIMU, RIGU) were applied to some pyridyl-imine moieties and counterions to facilitate anisotropic refinement. Geometry restraint dictionaries for certain ligands were generated using GRADE and GRADE2.^[7]^

The anions within the structure show some degree of disorder. The BF_4_^−^ anions were restrained to be tetrahedral. Only the major occupancy parts of counterions and solvent molecules were refined anisotropically. The strongest remaining electron density peaks are close to the modelled counterions and may indicate minor unresolved disorder. The occupancies of all located anions were allowed to freely refine which resulted in a discrepancy of ca. 4.08 anions per Cu_12_L_6_ assembly.

Further reflecting the solvent loss and poor diffraction properties there is a significant amount of void volume in the lattice containing smeared electron density from disordered solvent and the remaining anions. Consequently the SQUEEZE^[8]^ function of PLATON^[9]^ was employed to remove the contribution of the electron density associated with these remaining anions and further highly disordered solvent, which gave a potential solvent accessible void of 4186 Å^3^ per unit cell (a total of approximately 1281 electrons). The molecular weight and density given above may be underestimated due to insufficient resolution of the solvent molecules.


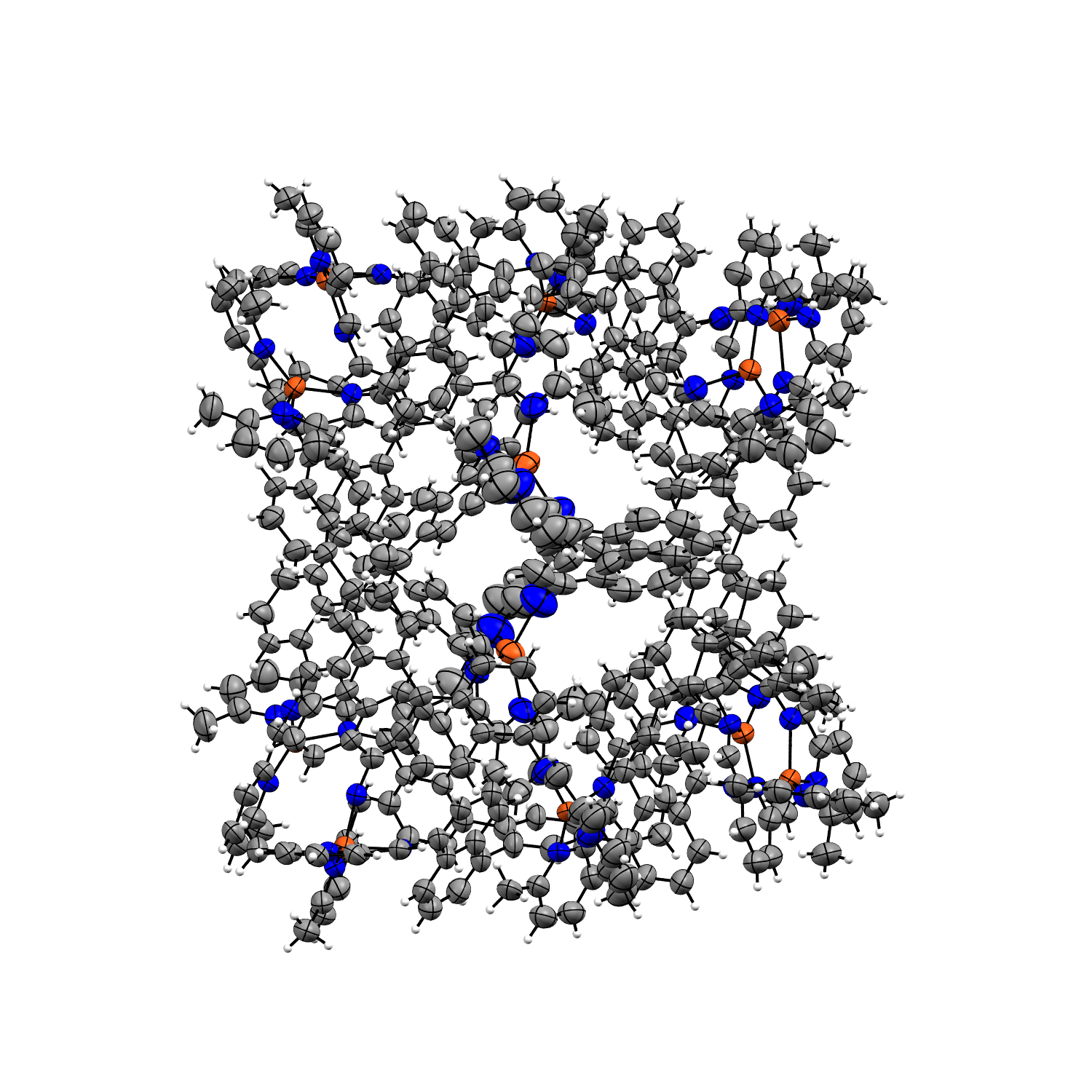


Figure S32. ORTEP plot of the cationic portion of the crystal structure of 1·(BF_4_)_12_, showing thermal ellipsoids at 50%. The asymmetric unit contains one half of the structure (color codes: C = gray, N = light blue, Zn = yellow, H = white).

Specific refinement details of **1**·(OTf)_12_:

The crystals of **1**·(CH_3_CN)_10.10_·(C_4_H_8_O_2_)_3.20_·(OTf)_12_ were grown by diffusion of ethyl acetate into an acetonitrile solution of **1**·(OTf)_12_. The crystals employed immediately lost solvent after removal from the mother liquor but rapid handling prior to flash cooling in liquid nitrogen and the use of synchrotron radiation was required to collect data. Despite these measures and the use of synchrotron radiation few reflections at greater than 0.9 Å resolution were observed and the data were trimmed accordingly. Nevertheless, the quality of the data is easily sufficient to establish the connectivity of the structure. The asymmetric unit was found to contain one complete Cu_12_L_6_ assembly and associated counterions and solvent molecules.

In order to obtain a reasonable model for the organic parts of the structure, DFIX restraints were applied to parts of the structure displaying a higher degree of thermal motion. Due to the thermal motion, thermal parameter restraints (SIMU, RIGU) were applied to some pyridyl-imine moieties and counterions to facilitate anisotropic refinement.

The anions within the structure show some degree of disorder. Only the major occupancy parts of counterions and solvent molecules were refined anisotropically. Some strong remaining electron density peaks are close to the modelled counterions or solvent molecules and may indicate minor unresolved disorder. The occupancies of all located anions were allowed to freely refine which resulted in a discrepancy of ca. 2.45 anions per Cu_12_L_6_ assembly.

Further reflecting the solvent loss and poor diffraction properties there is a significant amount of void volume in the lattice containing smeared electron density from disordered solvent and the remaining anions. Consequently the SQUEEZE^[8]^ function of PLATON^[9]^ was employed to remove the contribution of the electron density associated with these remaining anions and further highly disordered solvent, which gave a potential solvent accessible void of 1723 Å^3^ per unit cell (a total of approximately 504 electrons). The molecular weight and density given above may be underestimated due to insufficient resolution of the solvent molecules.


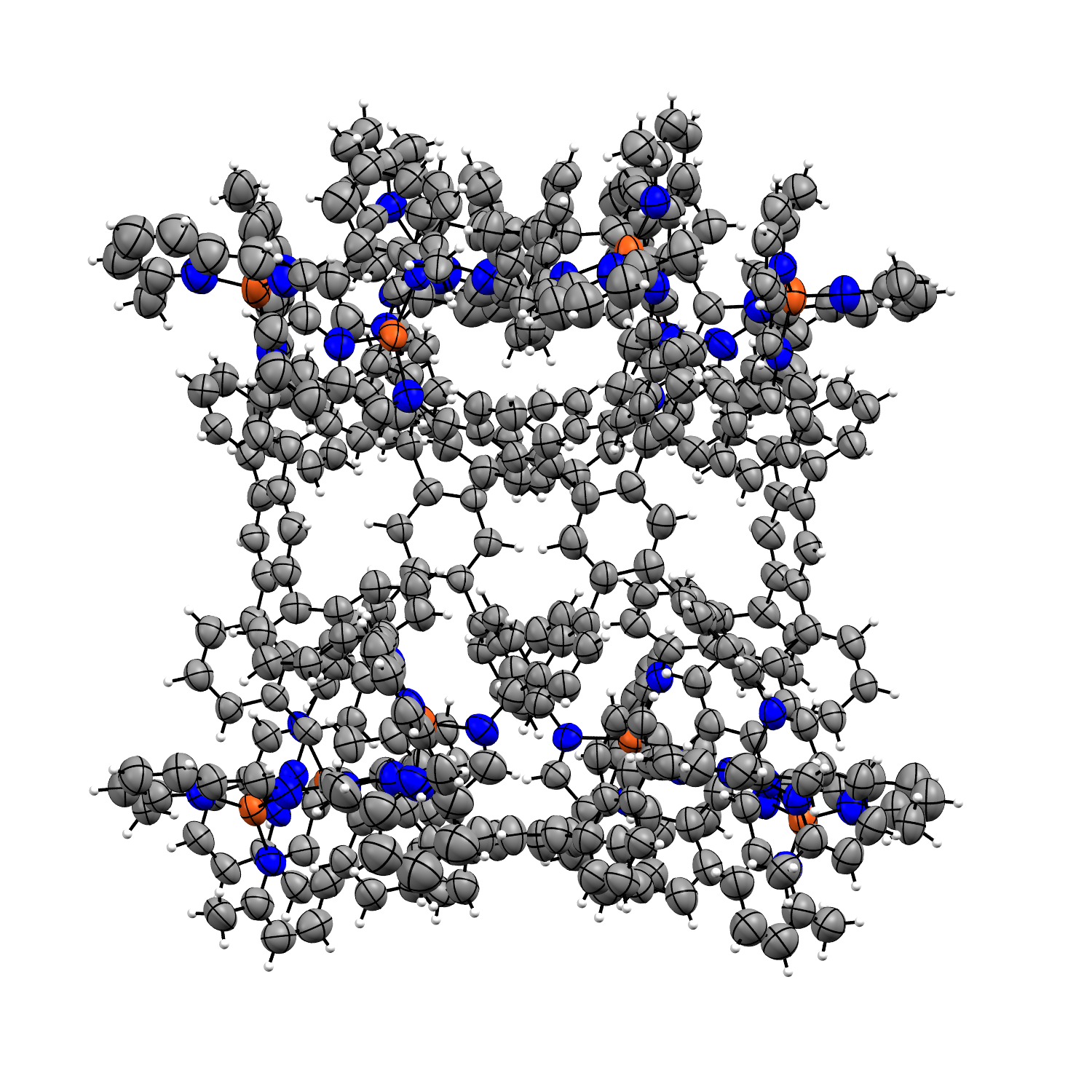


**Figure S33.** ORTEP plot of the cationic portion of the crystal structure of 1·(OTf)_12_, showing thermal ellipsoids at 50%. The asymmetric unit contains one half of the structure (color codes: C = grey, N = light blue, Zn = yellow, H = white).

Specific refinement details of **2**:

The crystals of **2**·(CH_3_CN)_8.90_·(CF_3_SO_3_)_8_ were grown by diffusion of ethyl acetate into an acetonitrile solution of **2**·(OTf)_8_. The crystals employed immediately lost solvent after removal from the mother liquor and rapid handling prior to flash cooling in liquid nitrogen was required to collect data. Despite these measures and the use of synchrotron radiation few reflections at greater than 1.2 Å resolution were observed and the data were trimmed accordingly. Nevertheless, the quality of the data is easily sufficient to establish the connectivity of the structure. The asymmetric unit was found to contain a Cu_4_L_2_ moiety, which comprises half of a Cu_8_L_4_ assembly and associated counterions and solvent molecules.

In order to obtain a reasonable model for the organic parts of the structure, DFIX restraints were applied to parts of the structure displaying a higher degree of thermal motion. Due to the thermal motion and less than ideal resolution, thermal parameter restraints (SIMU, RIGU) were applied to some pyridyl-imine moieties and counterions to facilitate anisotropic refinement. Geometry restraint dictionaries for certain ligands were generated using GRADE and GRADE2.^[7a]^ Due to the insufficient resolution of the data, all atoms except the Cu cations are refined isotropically in order to maximize the data to parameter ratio. The strongest remaining electron density peaks are close to the modelled counterions and may indicate minor unresolved. The hydrogen atoms of some acetonitrile molecules could not be located in the electron density map and were therefore not included in the model. The limited data resolution and significant amount of thermal motion also led to low precision on the length of C-C bonds throughout the structure.

The anions within the structure show some degree of disorder. The OTf^−^ anions were restrained to be in approximately staggered conformations. All anions were modelled with isotropic thermal parameters. The occupancies of all located anions were allowed to freely refine which resulted in a discrepancy of ca. 6.40 anions per Cu_8_L_4_ assembly.

Further reflecting the solvent loss and poor diffraction properties there is a significant amount of void volume in the lattice containing smeared electron density from disordered solvent and the remaining anions. Consequently the SQUEEZE^[8]^ function of PLATON^[9]^ was employed to remove the contribution of the electron density associated with these remaining anions and further highly disordered solvent, which gave a potential solvent accessible void of 18383 Å^3^ per unit cell (a total of approximately 6414 electrons). The molecular weight and density given above may be underestimated due to insufficient resolution of solvent molecules.

CheckCIF gives two A-level and three B-level alerts. These alerts result from the limited resolution of the data and the poor diffraction properties. One B level alert results from the acetonitrile molecules for which hydrogen atoms were not modelled.

# 4 Analysis of Secondary Interactions and Steric Hindrance

## 4.1 Analysis of Secondary Interactions

The unique C–H···π and π···π interactions identified in the single-crystal X-ray structures of cage **1**·(BF_4_)_12_ and **1**·(OTf)_12_ are summarised in the tables below. Geometric parameters were measured from the final refined structures using Mercury 2025.3.2.^[10]^ Aromatic stacking interactions were identified using a centroid-to-centroid distance cutoff of 4.5 Å.^[11]^ C–H···π interactions were identified using a C–H···centroid distance cutoff of 3.5 Å.^[12]^ For π···π contacts involving naphthalene units, centroids were defined using the six-membered ring directly involved in the interaction, whereas least-squares planes were defined using the full naphthalene unit. Only crystallographically unique interactions are listed. In cage **1**·(BF_4_)_12_, each asymmetric unit contains one half of the cage. The full cage thus contains twice the number of analogous secondary interactions listed in Tables S4-S5. In cage **1**·(OTf)_12_, each asymmetric unit contains one complete cage, so the number of secondary interactions corresponds directly to the entries in Tables S6-S7. Detailed definitions of ring codes and plane assignments are provided in the table footnotes.

Table S4. The C–H···π interactions in the crystal structure of cage 1·(BF_4_)_12_.

| **Donor Hydrogen^[a]^** | **Acceptor Ring^[b]^** | **H-Centroid Distance (H···C_g_, Å)** | **H-Plane Distance (Å)^[c]^** |
| --- | --- | --- | --- |
| H3B7 | 1A | 3.02 | 2.76 |
| H3A4 | 1A | 2.86 | 2.82 |
| H1B7 | 1B | 3.22 | 3.19 |
| H1A4 | 1B | 2.70 | 2.69 |
| H3A2 | 2A | 2.93 | 2.84 |
| H3A3 | 2A | 2.84 | 2.57 |
| H1A2 | 2B | 2.73 | 2.70 |
| H1A3 | 2B | 3.10 | 3.06 |
| H22C | 3A | 2.93 | 2.80 |
| H24A | 4A | 2.79 | 2.67 |
| H23B | 5A | 2.70 | 2.67 |
| H71C | 6A | 3.37 | 2.90 |
| H73C | 7A | 3.46 | 3.05 |
| H21A | 8A | 2.72 | 2.68 |
| H71B | 9A | 2.77 | 2.72 |
| H74C | 10A | 2.99 | 2.86 |

[a] Atom labels follow the corresponding .cif file (CCDC/KIZ deposition number: 2499903).

[b] Acceptor ring codes denote the six membered ring used for centroid calculation: 1A = C92C C82C C72C C62C C52C C110; 1B = C110 C42C C32C C22C C12C C92C; 2A = C112 C44C C34C C24C C14C C94C; 2B = C94C C84C C74C C64C C54C C112; 3A = C93B C107 C43B C33B C23B C13B; 4A = C94B C14B C24B C34B C44B C0AA; 5A = C106 C92B C12B C22B C32B C42B; 6A = C41B C31B C21B C11B C105 C91B; 7A = C84A C94A C104 C54A C64A C74A; 8A = C83A C73A C63A C53A C103 C93A; 9A = C22A C12A C92A C102 C42A C32A; 10A = C41A C91A C101 C11A C21A C31A.

[c] H···plane distances were measured to the least squares plane of the full naphthylene unit containing the corresponding acceptor ring: 1A/1B = N1 (C12C C22C C32C C42C C52C C62C C72C C82C C92C C110); 2A/2B = N2 (C14C C24C C34C C44C C54C C64C C74C C84C C94C C112); 3A = N3 (C13B C23B C33B C43B C53B C63B C73B C83B C93B C107); 4A = N4 (C14B C24B C34B C44B C54B C64B C74B C84B C94B C0AA); 5A = N5 (C12B C22B C32B C42B C52B C62B C72B C82B C92B C106); 6A = N6 (C11B C21B C31B C41B C51B C61B C71B C81B C91B C105); 7A = N7 (C14A C24A C34A C44A C54A C64A C74A C84A C94A C104); 8A = N8 (C13A C23A C33A C43A C53A C63A C73A C83A C93A C103); 9A = N9 (C12A C22A C32A C42A C52A C62A C72A C82A C92A C102); 10A = N10 (C11A C21A C31A C41A C51A C61A C71A C81A C91A C101).

Table S5. The aromatic stacking interactions in the crystal structure of cage 1·(BF_4_)_12_.

| **Ring 1^[a]^** | **Ring 2^[a]^** | **Plane-Plane Distance (Mean Centroid-Plane Distance, Å)^[b]^** | **Centroid-Centroid Distance (C_g_···C_g_, Å)** | **Slippage (Å)^[b]^** | **Dihedral Angle *θ*(°)^[b]^** |
| --- | --- | --- | --- | --- | --- |
| 8A | 9A | 3.53 | 3.77 | 1.33 | 7.76 |
| 9A | 10A | 3.29 | 4.13 | 2.49 | 27.79 |
| 10B | 11A | 3.82 | 3.98 | 1.13 | 18.19 |
| 12A | 13A | 3.53 | 3.71 | 1.15 | 5.72 |
| 13A | 14A | 3.34 | 4.05 | 2.28 | 27.61 |
| 14B | 15A | 3.70 | 3.91 | 1.24 | 18.05 |

[a] Ring codes denote the six membered ring used for centroid calculation; atom labels follow the corresponding .cif file (CCDC/KIZ deposition number: 2499903): 8A = N2A6 C6A6 C5A6 C4A6 C3A6 C2A6; 9A = C4B7 C3B7 C2B7 N2B7 C6B7 C5B7; 10A = C21C C11C C31C C41C C109 C91C; 10B = C91C C109 C51C C61C C71C C81C; 11A = C4A2 C3A2 C2A2 N2A2 C6A2 C5A2; 12A = C5A1 C4A1 C3A1 C2A1 N2A1 C6A1; 13A = C6A3 N2A3 C5A3 C4A3 C3A3 C2A3; 14A = C111 C93C C13C C23C C33C C43C; 14B = C53C C111 C93C C83C C73C C63C; 15A = N2A4 C6A4 C5A4 C4A4 C3A4 C2A4.

[b] Plane based distances were calculated to the least squares planes of the full pyridyl or naphthylene unit containing the corresponding ring code. For pyridyl rings, the plane is defined by the same six atoms listed in [a]. For naphthylenes: 10A/10B = N8 (C11C C21C C31C C41C C51C C61C C71C C81C C91C C109); 14A/14B = N9 (C13C C23C C33C C43C C53C C63C C73C C83C C93C C111).


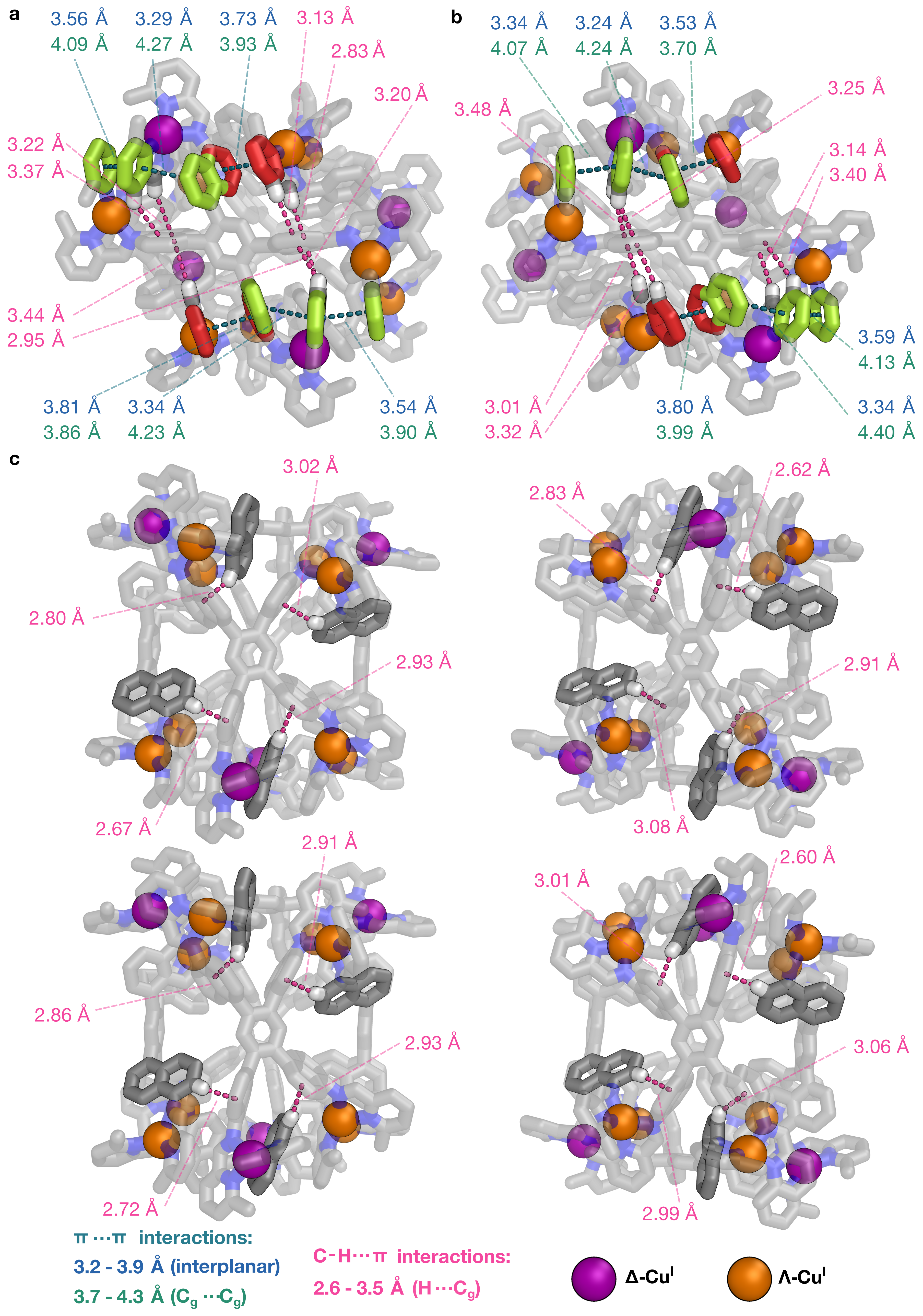


Figure S34. a Top, b bottom and c side views of the crystal structure of 1·(OTf)_12_, showing π···π and C–H···π interactions highlighted in teal and pink, respectively. For π···π interactions, interplanar distances are highlighted in blue and centroid-centroid (C_g_···C_g_) distances in green; for C–H···π interactions, the hydrogen-centroid distances are highlighted in pink. Color codes: C = lime (one group of stacking aromatic rings), red (another pair of stacking aromatic rings) or gray, H = white, N = blue, Δ-Cu = purple, Λ-Cu = orange. Hydrogen atoms not involved in C–H···π interactions, counterions and solvent molecules have been omitted for clarity.

Table S6. The C–H···π interactions in the crystal structure of cage 1·(OTf)_12_.

| **Donor Hydrogen^[a]^** | **Acceptor Ring^[b]^** | **H-Centroid Distance (H···C_g_, Å)** | **H-Plane Distance (Å)^[c]^** |
| --- | --- | --- | --- |
| H5B9 | 1A | 3.22 | 2.72 |
| H3B9 | 1B | 3.37 | 3.19 |
| H3A7 | 1B | 3.44 | 3.15 |
| H5AC | 2A | 3.13 | 2.99 |
| H5BB | 2A | 2.95 | 2.66 |
| H3AC | 2B | 2.83 | 2.74 |
| H3BB | 2B | 3.20 | 3.13 |
| H5A4 | 3A | 3.25 | 2.85 |
| H5B1 | 3A | 3.32 | 3.09 |
| H3A4 | 3B | 3.48 | 3.27 |
| H3B1 | 3B | 3.01 | 2.89 |
| H5A2 | 4A | 3.40 | 2.72 |
| H3A2 | 4B | 3.14 | 2.96 |
| H74E | 5A | 2.80 | 2.78 |
| H23C | 6A | 3.02 | 2.79 |
| H21A | 7A | 2.67 | 2.64 |
| H71F | 8A | 2.93 | 2.75 |
| H71E | 9A | 2.83 | 2.69 |
| H74B | 10A | 2.62 | 2.60 |
| H72D | 11A | 3.08 | 3.00 |
| H72F | 12A | 2.91 | 2.59 |
| H72E | 13A | 2.86 | 2.81 |
| H72B | 14A | 2.99 | 2.78 |
| H73A | 15A | 2.91 | 2.73 |
| H71C | 16A | 2.72 | 2.69 |
| H74F | 17A | 2.93 | 2.68 |
| H73E | 18A | 3.01 | 2.74 |
| H23F | 19A | 3.06 | 3.05 |
| H23D | 20A | 2.60 | 2.59 |

[a] Atom labels follow the corresponding .cif file (CCDC/KIZ deposition number: 2531235).

[b] Acceptor ring codes denote the six membered ring used for centroid calculation:

1A = C14E C24E C34E C44E C120 C94E, 1B = C120 C94E C84E C74E C64E C54E, 2A = C42E C32E C22E C12E C92E C118, 2B = C92E C118 C52E C62E C72E C82E, 3A = C73F C83F C93F C123 C53F C63F, 3B = C93F C13F C23F C33F C43F C123, 4A = C12F C22F C32F C42F C122 C92F, 4B = C122 C92F C52F C62F C82F C72F, 5A = C13D C93D C115 C43D C33D C23D, 6A = C84D C94D C116 C54D C64D C74D, 7A = C51D C61D C71D C81D C91D C113, 8A = centroid: C32D C22D C12D C92D C114 C42D, 9A = centroid: C93C C111 C53C C63C C73C C83C, 10A = centroid: C24C C34C C44C C112 C94C C14C, 11A = centroid: C32C C22C C12C C92C C110 C42C, 12A = centroid: C109 C51C C61C C71C C91C C81C, 13A = centroid: C84B C94B C108 C54B C64B C74B, 14A = C44A C104 C94A C14A C24A C34A, 15A = centroid: C93B C107 C43B C33B C23B C13B, 16A = centroid: C61B C71B C81B C91B C105 C51B, 17A = centroid: C42B C32B C22B C12B C92B C106, 17A = centroid: C93A C103 C43A C33A C23A C13A, 19A = centroid: C101 C91A C11A C21A C31A C41A, 20A = centroid: C72A C62A C52A C102 C92A C82A

[c] H···plane distances were measured to the least squares plane of the full naphthylene unit containing the corresponding acceptor ring: 1A/1B = N1 (C14E C24E C34E C44E C54E C64E C74E C84E C94E C120); 2A/2B = N2 (C12E C22E C32E C42E C52E C62E C72E C82E C92E C118); 3A/3B = N3 (C13F C23F C33F C43F C53F C63F C73F C83F C93F C123); 4A/4B = N4 (C12F C22F C32F C42F C52F C62F C72F C82F C92F C122); 5A = N5 (C13D C23D C33D C43D C53D C63D C73D C83D C93D C115); 6A = N6 (C14D C24D C34D C44D C54D C64D C74D C84D C94D C116); 7A = N7 (C11D C21D C31D C41D C51D C61D C71D C81D C91D C113); 8A = N8 (C12D C22D C32D C42D C52D C62D C72D C82D C92D C114); 9A = N9 (C13C C23C C33C C43C C53C C63C C73C C83C C93C C111); 10A = N10 (C14C C24C C34C C44C C54C C64C C74C C84C C94C C112); 11A = N11 (C12C C22C C32C C42C C52C C62C C72C C82C C92C C110); 12A = N12 (C11C C21C C31C C41C C51C C61C C71C C81C C91C C109); 13A = N13 (C14B C24B C34B C44B C54B C64B C74B C84B C94B C108); 14A = N14 (C14A C24A C34A C44A C54A C64A C74A C84A C94A C104); 15A = N15 (C13B C23B C33B C43B C53B C63B C73B C83B C93B C107); 16A = N16 (C11B C21B C31B C41B C51B C61B C71B C81B C91B C105); 17A = N17 (C12B C22B C32B C42B C52B C62B C72B C82B C92B C106); 18A = N18 (C13A C23A C33A C43A C53A C63A C73A C83A C93A C103); 19A = N19 (C11A C21A C31A C41A C51A C61A C71A C81A C91A C101); 20A = N20 (C12A C22A C32A C42A C52A C62A C72A C82A C92A C102).

Table S7. The aromatic stacking in the crystal structure of cage 1·(OTf)_12_.

| **Ring 1^[a]^** | **Ring 2^[a]^** | **Plane-Plane Distance (Mean Centroid-Plane Distance, Å)^[b]^** | **Centroid-Centroid Distance (C_g_···C_g_, Å)** | **Slippage (Å)^[b]^** | **Dihedral Angle *θ* (°)^[b]^** |
| --- | --- | --- | --- | --- | --- |
| 20A | 21A | 3.56 | 4.09 | 2.01 | 11.95 |
| 21A | 22A | 3.29 | 4.27 | 2.72 | 29.7 |
| 22B | 23A | 3.73 | 3.93 | 1.23 | 12.18 |
| 24A | 25A | 3.54 | 3.90 | 1.62 | 10.13 |
| 25A | 26A | 3.34 | 4.23 | 2.59 | 26.73 |
| 26B | 27A | 3.81 | 3.86 | 0.59 | 3.75 |
| 28A | 29A | 3.34 | 4.07 | 2.32 | 8.6 |
| 29A | 30A | 3.24 | 4.24 | 2.73 | 29.82 |
| 30B | 31A | 3.53 | 3.70 | 1.13 | 6.51 |
| 32A | 33A | 3.59 | 4.13 | 2.05 | 18.55 |
| 33A | 34A | 3.34 | 4.40 | 2.87 | 33.81 |
| 34B | 35A | 3.80 | 3.99 | 1.21 | 12.26 |

[a] Ring codes denote the six membered ring used for centroid calculation, with atom labels follow the corresponding .cif file (CCDC/KIZ deposition number: 2531235): 20A = C5B8 C4B8 N2B8 C8B8 C7B8 C6B8; 21A = N2B9 C4B9 C5B9 C6B9 C7B9 C8B9; 22A = C23E C33E C43E C119 C93E C13E; 22B = C119 C93E C83E C73E C63E C53E; 23A = C7AC C6AC C5AC C4AC N2AC C8AC; 24A = C8BA N2BA C4BA C5BA C6BA C7BA; 25A = C4BB C5BB C6BB C7BB C8BB N2BB; 26A = C117 C91E C21E C11E C41E C31E; 26B = C81E C71E C61E C51E C117 C91E; 27A = C7A7 C6A7 C5A7 C4A7 N2A7 C8A7; 28A = C7B5 C8B5 N2B5 C4B5 C5B5 C6B5; 29A = C8A4 C7A4 C6A4 C5A4 C4A4 N2A4; 30A = C11F C21F C31F C41F C121 C91F; 30B = C121 C91F C81F C71F C61F C51F; 31A = C8A6 C7A6 C6A6 C5A6 C4A6 N2A6; 32A = C4B3 N2B3 C8B3 C7B3 C6B3 C5B3; 33A = C4A2 C5A2 C6A2 C7A2 C8A2 N2A2; 34A = C124 C94F C14F C24F C34F C44F; 34B = C124 C94F C54F C64F C74F C84F; 35A = C8B1 N2B1 C4B1 C5B1 C6B1 C7B1.

[b] Plane-based distances were calculated to the least-squares planes of the full pyridyl or naphthylene unit containing the corresponding ring code. For pyridyl rings, the plane is defined by the same six atoms listed in [a]. For naphthylenes: 22A/22B = N20 (C13E C23E C33E C43E C53E C63E C73E C83E C93E C119); 26A/26B = N21 (C11E C21E C31E C41E C51E C61E C71E C81E C91E C117); 30A/30B = N22 (C11F C21F C31F C41F C51F C61F C71F C81F C91F C121); 34A/34B = N23 (C14F C24F C34F C44F C54F C64F C74F C84F C94F C124).

## 4.2 Analysis of Steric Hindrance

Starting from the crystal structure of **1**·(BF_4_)_12_ in XYZ format, Python with RDKit^[13]^ and NumPy^[14]^ was used to convert all 6-methyl-2-formylpyridine residues into 3-methyl-2-formylpyridine (**C**) residues by removing each methyl substituent at the 6-position and replacing it with a hydrogen atom, while simultaneously substituting the hydrogen atom at the 3-position of the same pyridyl ring with a methyl group. Hydrogen-to-methyl replacements were performed by placing a methyl fragment, pre-embedded in three dimensions using RDKit, at a C–C distance of 1.54 Å along the original C–H bond vector. The methyl fragment was oriented by aligning one of its C–H vectors antiparallel to the newly formed C–C bond. Methyl-to-hydrogen replacements were carried out by removing the four atoms of the methyl group, calculating the vector from the ring carbon to the center of mass of the removed methyl group, and placing a hydrogen atom at a C–H distance of 1.09 Å along this vector. All atomic coordinates not directly involved in these transformations were preserved from the original structure without geometry optimization, thereby maintaining any steric clashes that demonstrate the structural infeasibility of the fully converted 3-formyl-2-formylpyridine analogue.

Figure S34 shows the comparison between the crystal structure of **1**·(BF_4_)_12_ and its corresponding model with 3-methyl-2-formylpyridine (**C**) residues. The model shows that the 3-methyls of **C**-residues generate significant steric clashes with the cage backbone, potentially rendering this geometry unfeasible. Specifically, as highlighted in Figure S34b, short C···C distances between these methyl groups and the nearest naphthylene carbons, ranging from 2.04 to 2.49 Å, were observed. These distances are shorter than the sum of the van der Waals radii of methyl groups (~2.0 Å^[15]^) and carbon atoms (~1.77 Å^[16]^), and would thus indicate steric hindrance that destabilizes the framework of **1**·(BF_4_)_12_.


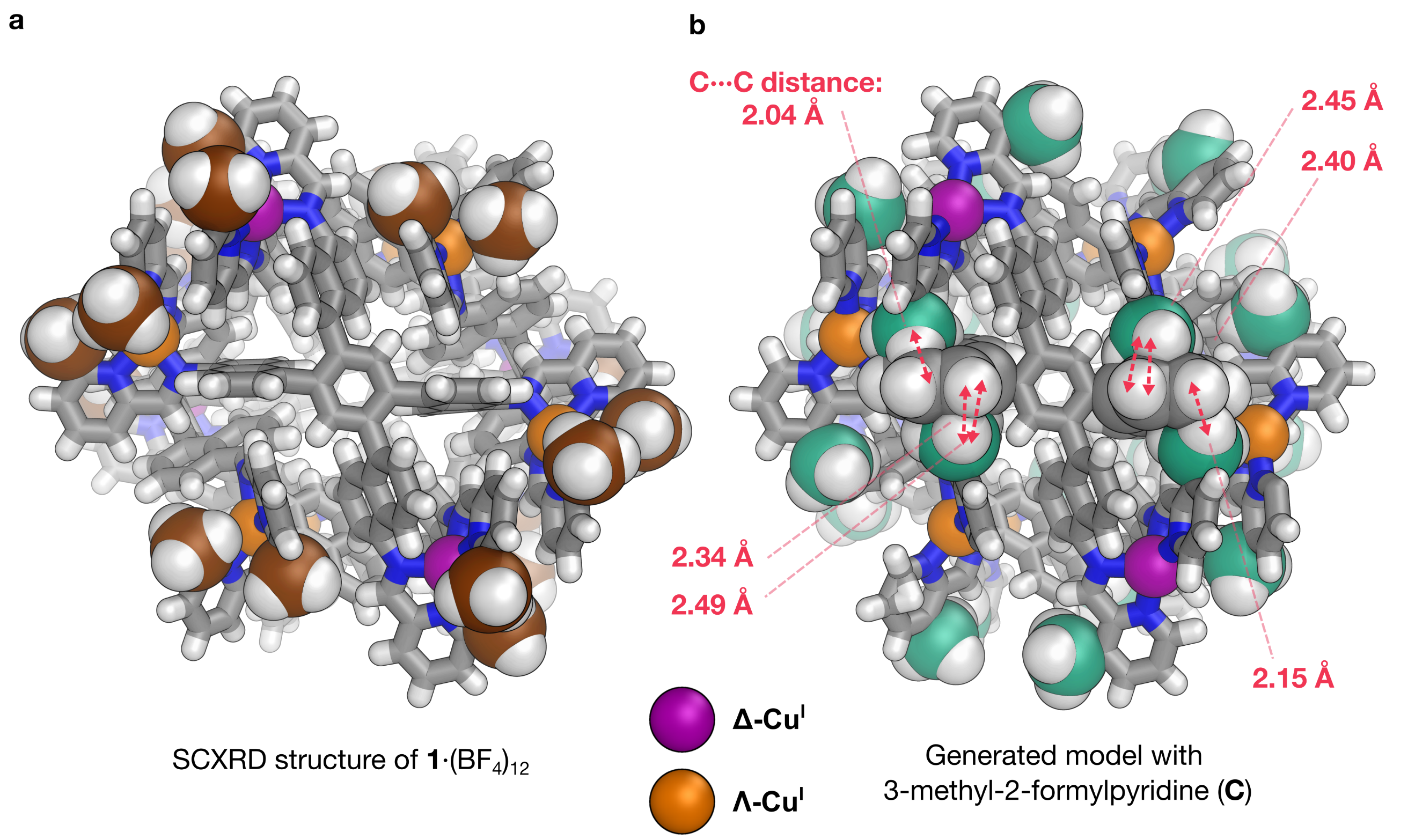


**Figure S35.** a Top view of the crystal structure of 1·(BF_4_)_12_, with methyl groups from the 6-methyl-2-formylpyridine (B) residues highlighted in space-filling models. b Top view of a hypothetical model of -an analogue of 1·(BF_4_)_12_ bearing 3-methyl-2-formylpyridine (C) residues, generated using Python with RDKit^[13]^ and NumPy^[14]^. The model was created by replacing the 6-methyl substituents with hydrogen atoms and the 3-hydrogen atoms of the pyridyl residues with methyl groups, while preserving all other atomic positions.The methyl groups and portions of the naphthylene moieties are rendered as green and grey space-filling models to illustrate steric clashes between certain 3-methyl-2-formylpyridine residues and adjacent naphthylene units. The C···C distances indicating steric clashes are highlighted in red. Colour scheme: C = brown (methyl groups in 6-methyl-2-formylpyridine (B) residues), green (methyl groups in 3-methyl-2-formylpyridine (C) residues), or grey, H = white, N = blue, Δ-Cu = purple, Λ-Cu = orange. Hydrogen atoms are only shown in the space-filling regions, counterions, and solvent molecules have been omitted for clarity.

The self-assembly with 3-methyl-2-formylpyridine (**C**) instead of 6-methyl-2-formylpyridine (**B**) with subcomponent **A** and Cu^I^(MeCN)_4_OTf generated structure **2**. As shown in Figure S35a, the methyls from the **C**-residues were not found to be in close proximity with any other structural moieties of the cage to generate significant steric clashes.

Similarly, we generated a hypothetical model of the analogous structure based on this structure with 6-methyl-2-formylpyridine (**B**) residues to compare the steric effects of the two types of residues on the structure, in order to identify the factors that determine the formation of the structures of **1** and **2** under different conditions. As shown in Figure S35b, when methyl substituents are moved from the 3-positions of the pyridyl residues to the 6-positions, no steric hindrance was observed that would adversely affect the formation of the structure of **2**. Therefore, neither aldehyde residue is considered to potentially generating steric hindrance that disrupts the framework of **2**.


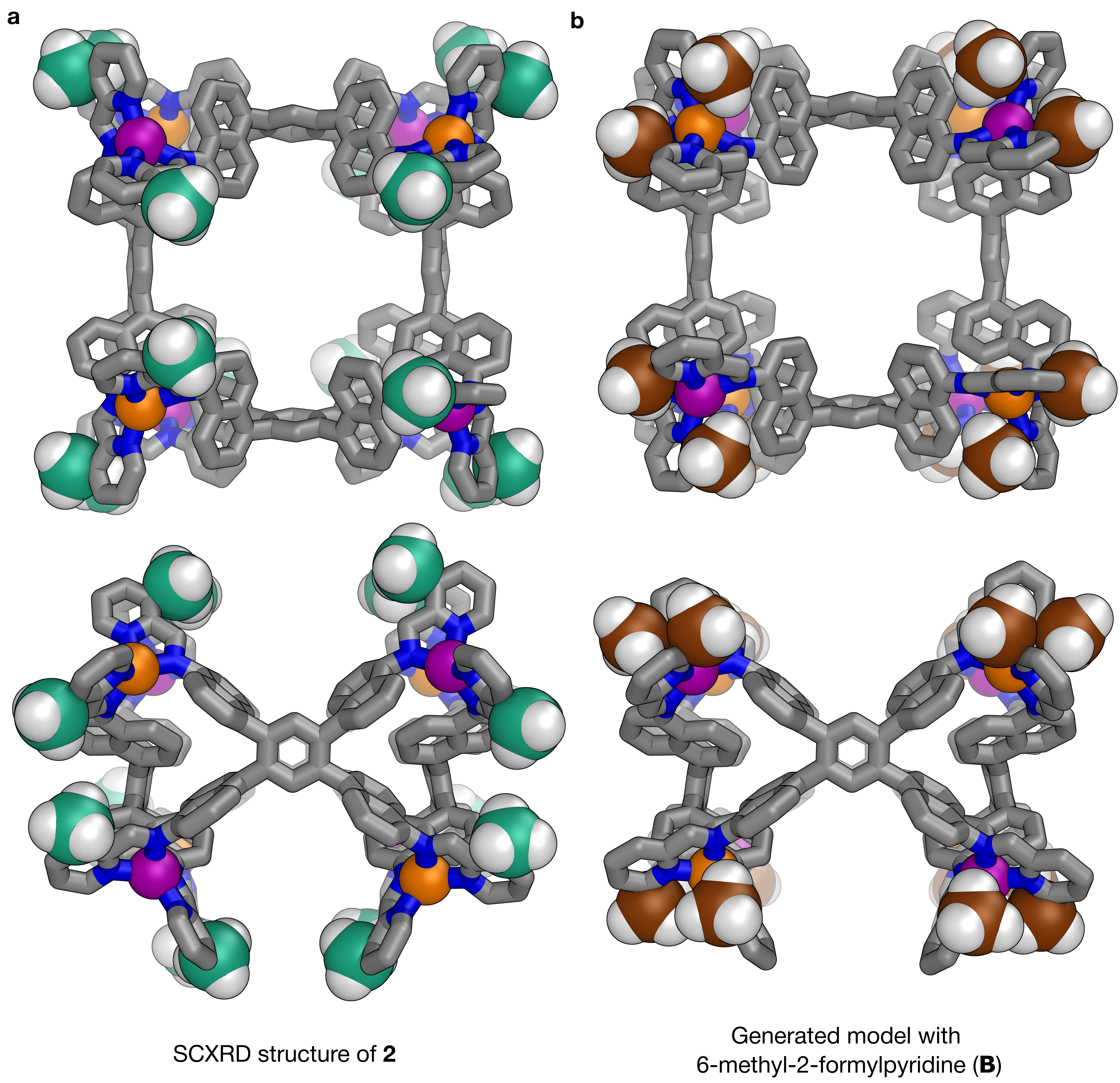


Figure S36**.** a Top view of the crystal structure of 2, with methyl groups from the 3-methyl-2-formylpyridine (C) residues highlighted in space-filling models. b Top view of a hypothetical model of an analogue of 2 bearing 6-methyl-2-formylpyridine (B) residues with methyls rendered in space-filling models, generated using Python with RDKit^[13]^ and NumPy^[14]^. The model was created by replacing the 3-methyl substituents with hydrogen atoms and the 6-hydrogen atoms of the pyridyl residues with methyl groups, while preserving all other atomic positions. The C···C distances indicating steric clashes are highlighted in red. Colour scheme: C = brown (methyl groups in 6-methyl-2-formylpyridine (B) residues), green (methyl groups in 3-methyl-2-formylpyridine (C) residues), or grey, H = white, N = blue, Δ-Cu = purple, Λ-Cu = orange. Hydrogen atoms are only shown in the space-filling regions, counterions, and solvent molecules have been omitted for clarity.

# 5 Reconfiguration from 2 to generate 1

**2**·(OTf)_8_ (10.00 mg, 1.69 μmol) was dissolved in degassed acetonitrile (500 μL). 6-methyl-2-formylpyridine (60.0 mmol·L^-1^ in CH_3_CN, 1013 μL, 36.0 equiv.) was added and the mixture was degassed via three freeze-pump-thaw cycles before heating at 70°C for 64 h and monitored by ESI-MS. ^1^H NMR was recorded directly from this solution and a small portion was taken for ESI-MS analysis (Figures S37, S39 and S41a). The mixture was subsequently filtered through a plug with glass fiber to ensure no visible precipitate was generated, concentrated under N_2_ flow to 0.5 mL, and precipitated with Et_2_O (15 mL). The precipitate was collected by centrifugation, washed with Et_2_O (3 × 15 mL), dried *in vacuo*, and redissolved in CD_3_CN (0.5 mL) for ^1^H NMR analysis (Figure S39 and S41). The comparison of ^1^H NMR spectra before and after workup and the ^1^H DOSY (Figure S40) spectrum of the product are as following:


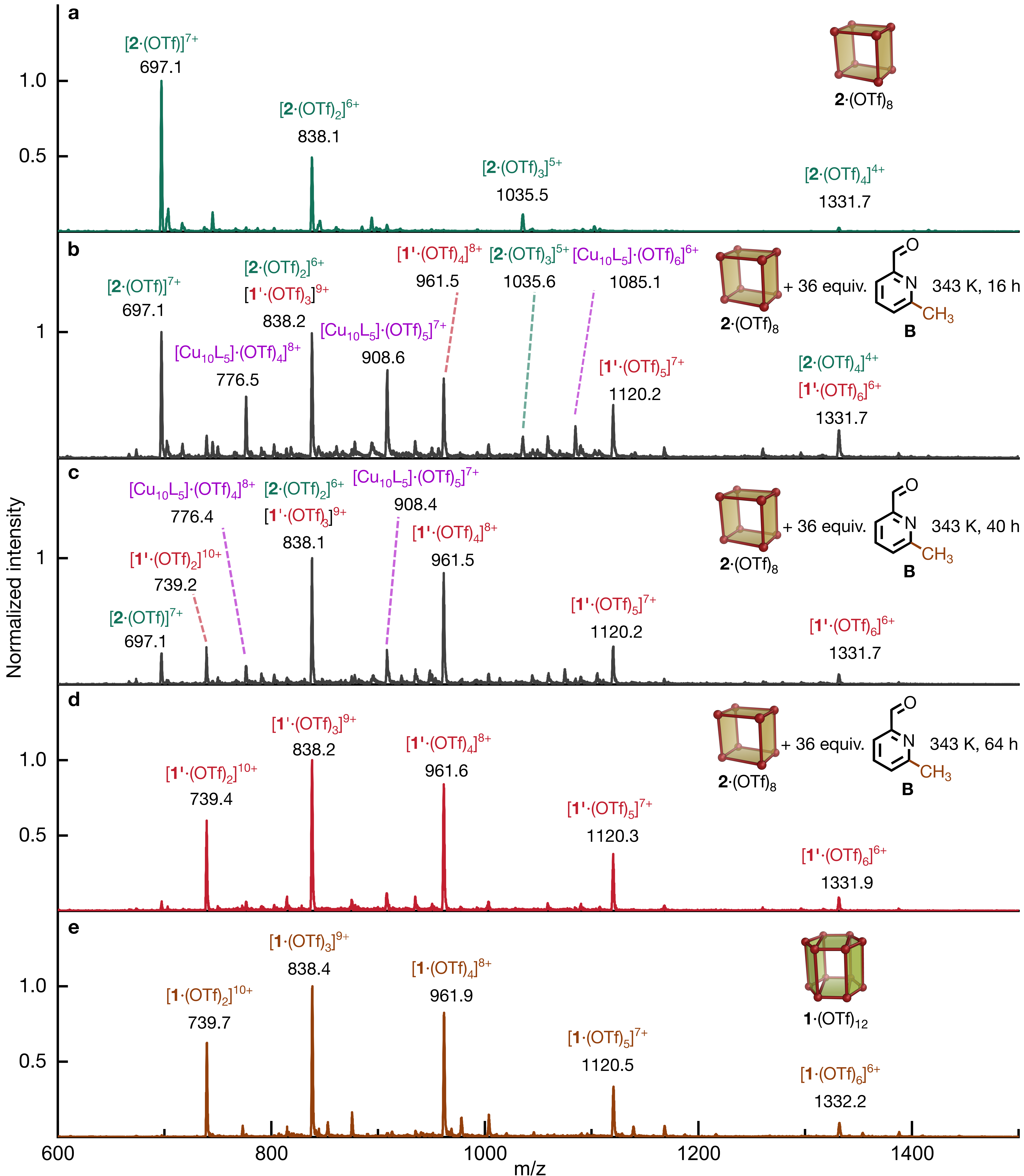


Figure S37. Stacked ESI-MS spectra tracking the structural conversion from 2 to 1. a ESI-MS spectrum of 2·(OTf)_8_. b-d ESI-MS spectrum of the same sample after addition of 36 equiv. of 6-methyl-2-formylpyridine (B) and heating at 343 K for b 16 h c 40 h d 64 h. e ESI-MS spectrum of a sample of 1·(OTf)_12_ for reference.


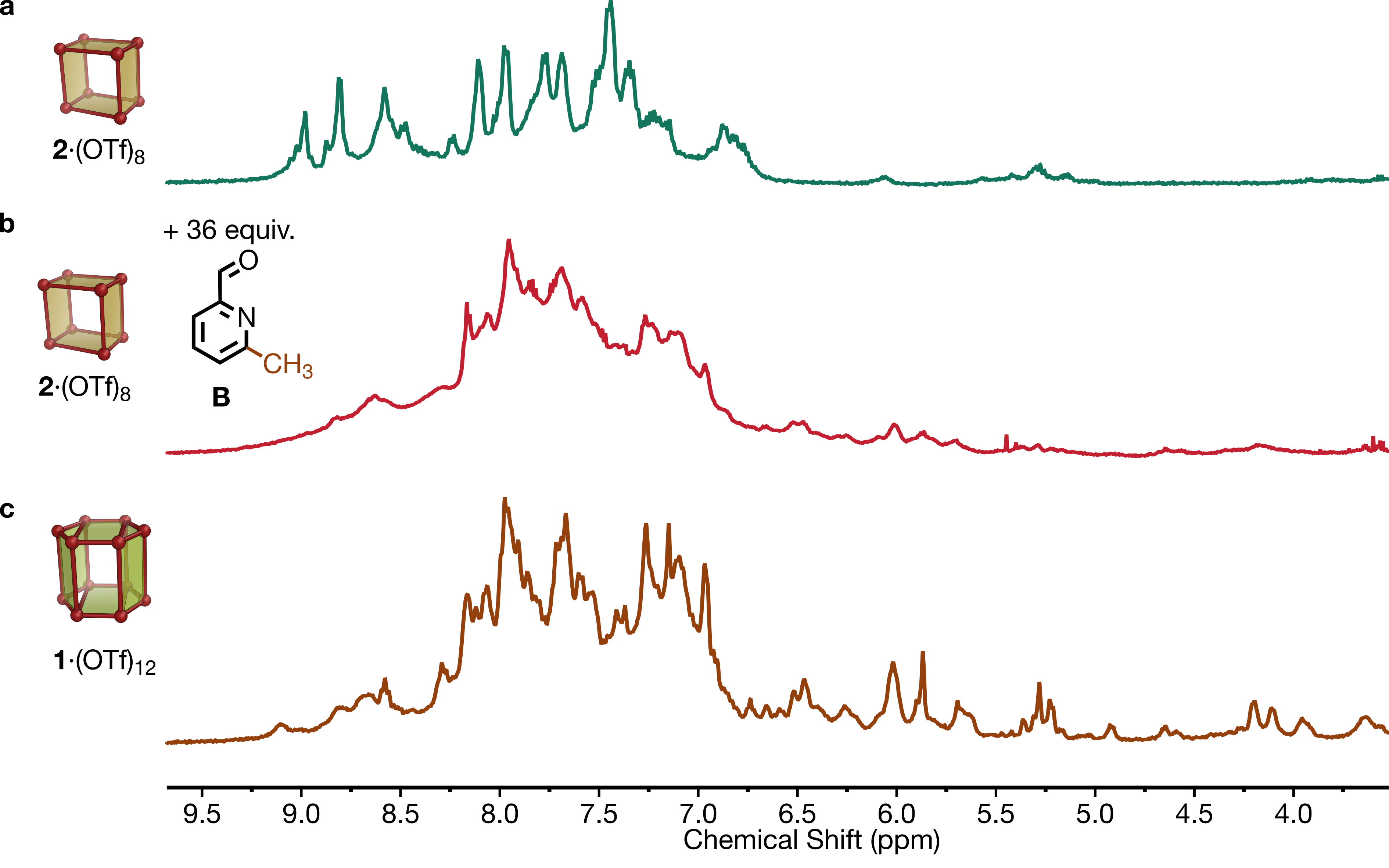


Figure S38. Stacked ^1^H NMR spectra tracking the structural conversion from 2 to 1. a ^1^H NMR spectrum of 2·(OTf)_8_ (500 MHz, CD_3_CN, 298 K). b ^1^H NMR spectrum of the solution of 2·(OTf)_8_ (4.05 mM in CD_3_CN) after addition of a CD_3_CN solution of 36 equiv. of 6-methyl-2-formylpyridine (B, 60.00 mM in CD_3_CN, 1013 μL) and heating at 343 K for 64 h, following workup with Et_2_O washing. c ^1^H NMR spectrum of 1·(OTf)_12_ (500 MHz, CD_3_CN, 298 K). The synthesis of 1·(OTf)_12_ is identical to that of 1·(BF_4_)_12_, except that Cu(MeCN)_4_OTf is used instead of Cu(MeCN)_4_BF_4_.


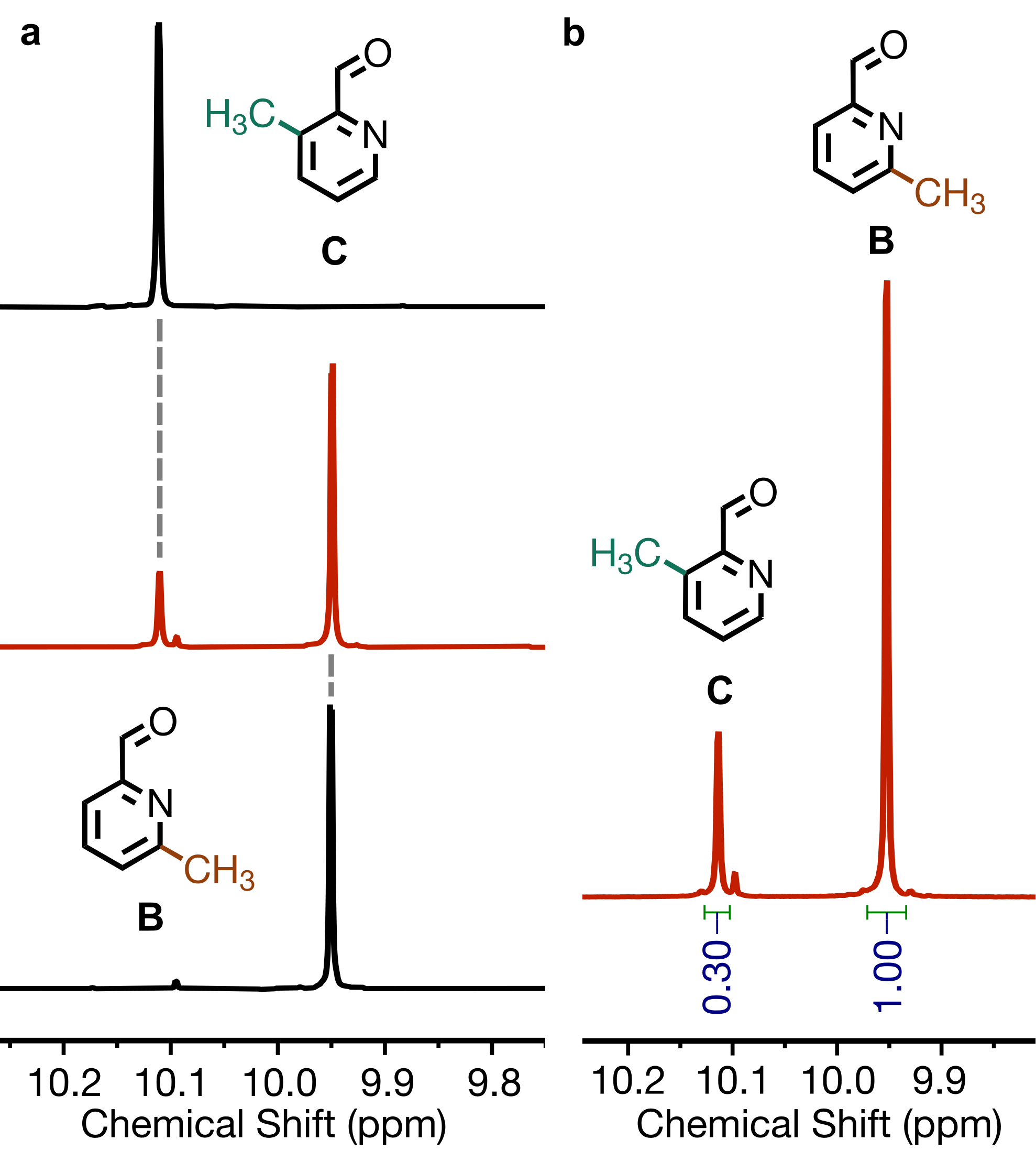


**Figure S39**. Aldehyde region of the ^1^H NMR spectrum of the same sample after addition of 36 equiv. of 6-methyl-2-formylpyridine (B) and heating at 343 K for 64 h, before work-up with Et_2_O washing. a comparison with ^1^H NMR spectra of pure B and C, b integrals of its aldehyde signals.

Figure S39 shows the aldehyde region of the ^1^H NMR spectrum of the sample after the transformation attempt, prior to work-up, showing a 0.30 : 1.00 ratio of displaced free 3-methyl-2-formylpyridine (**C**) to remaining free 6-methyl-2-formylpyridine (**B**). The signal at 10.11 ppm was assigned to free **C** released during exchange, while the signal at 9.95 ppm was assigned to remaining free **B**, based on comparison with the reference spectra of **C** and **B** in Figure S39a. Integrals were measured using MestReNova 12.0.0^[17]^ following standard automatic baseline correction and were normalized to proton count. As a precise amount of **B** was added and no visible precipitate was observed, confirmed by filtration before work-up, the average number of **C** and **B** residues in **1’**, the post-transformation [Cu^I^_12_L_6_]^12+^, or the number of displaced aldehyde residues per cage, can be estimated assuming all species derived from either aldehyde remain in solution throughout the process. This can be done using the equation below:

Let *x* be the number of aldehydes replaced per [Cu^I^_12_L_6_]^12+^ cage analogous to **1**. From Figure S39:

$$\frac{\text{x}}{\text{36 - }\text{x}}\text{=}\frac{\text{0.30}}{\text{1.00}}$$

Solving for *x*:

*x* ≈ 8.3, and 24 - *x* ≈ 15.7.

Therefore, to convert the sample of **2** to a [Cu^I^_12_L_6_]^12+^ structure analogous to **1**, only ~8.3 **C** residues were actually replaced by **B** per [Cu^I^_12_L_6_]^12+^ cage formed from 1.5 [Cu^I^_8_L_4_]^8+^ cages, with the remaining ~15.7 being **C** residues.

Thus, on average, ca. 8.3 **C** residues were replaced by **B** per cage, leaving ca. 15.7 C derived residues in the post-transformation assembly **1’**. This value is approximate because of minor uncertainty in baseline definition.


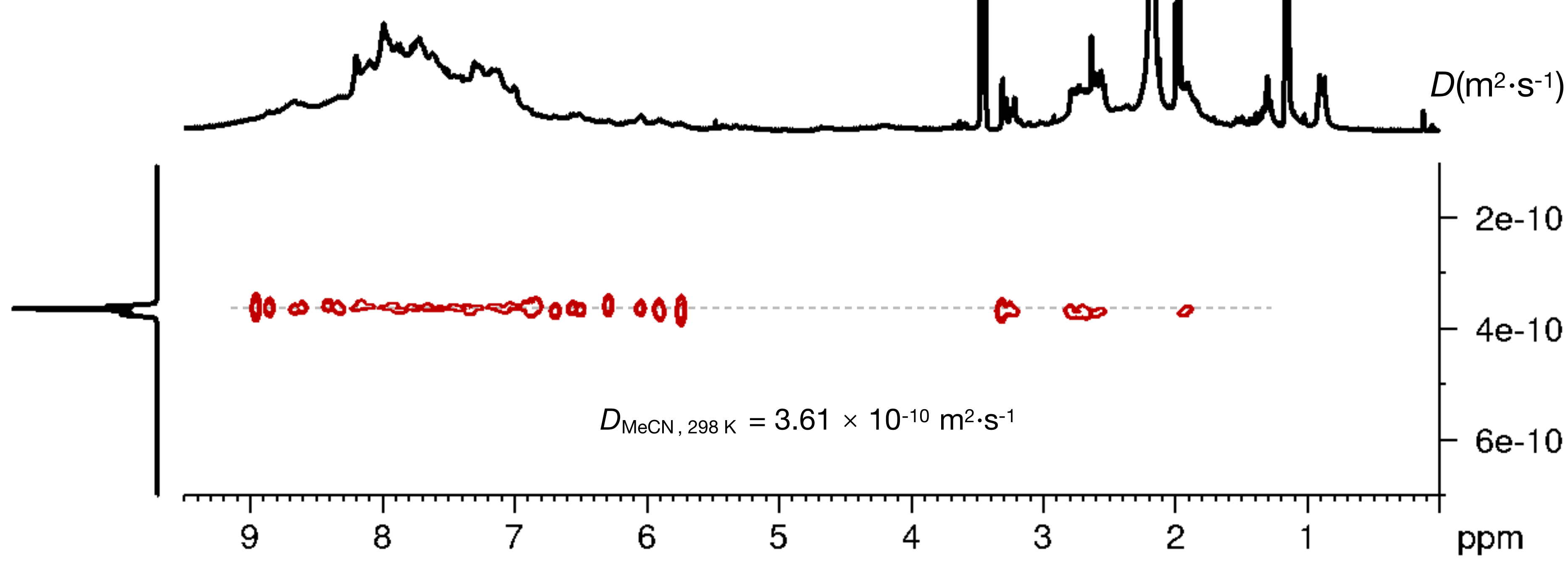


Figure S40. ^1^H DOSY spectrum of the [Cu^I^_12_L_6_]·(OTf)_12_ product after transformation from cage 2·(OTf)_8_ (400 MHz, CD_3_CN, 298 K). The uniform diffusion coefficients of the signals suggest the presence of a single species. An average diffusion coefficient (*D*) of 3.61 × 10^-10^ m^2^·s^-1^ is revealed.


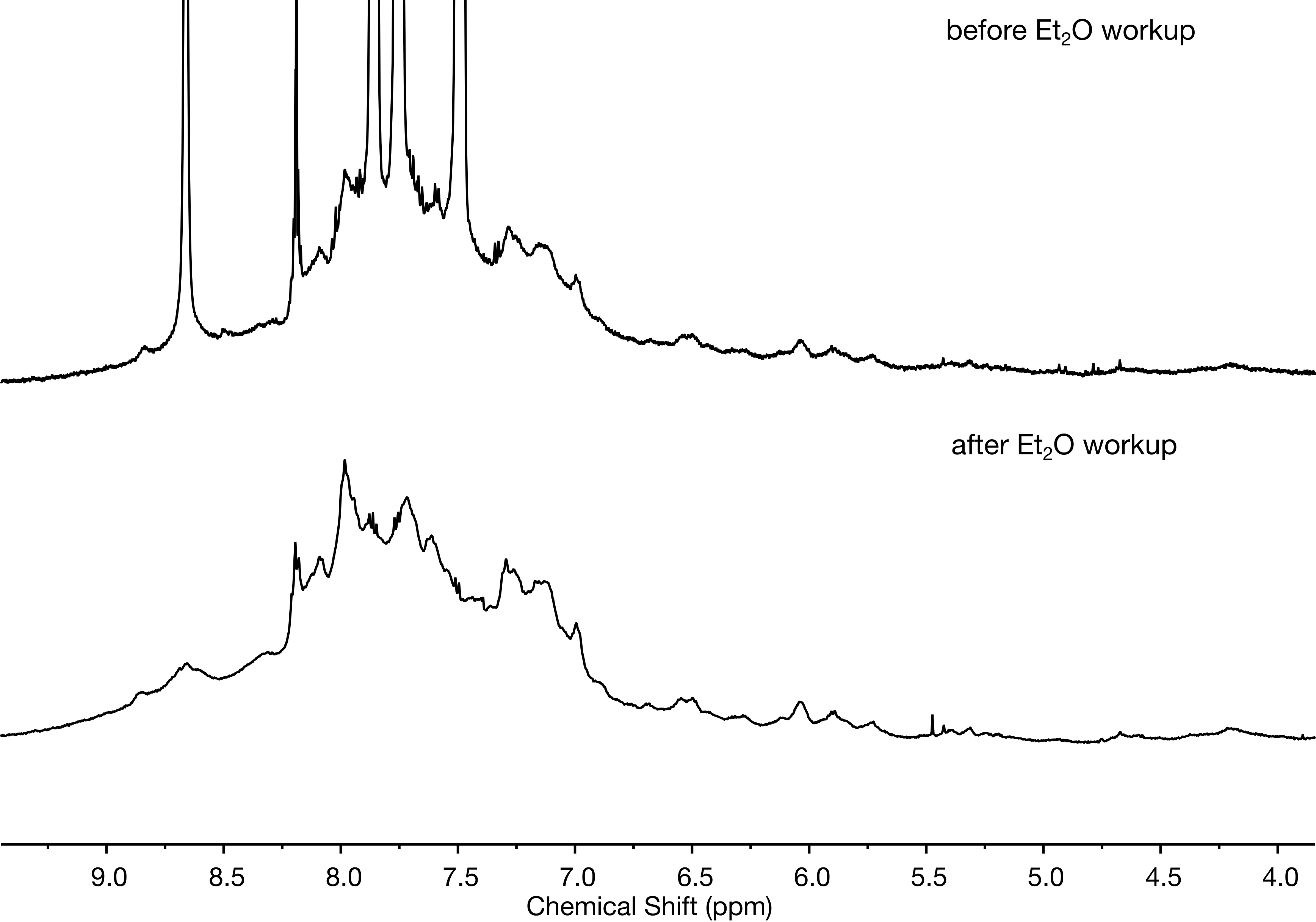


Figure S41. The stacked ^1^H NMR spectra (500 MHz, CD_3_CN, 298 K) of the [Cu^I^_12_L_6_]·(OTf)_12_ product 1’ after transformation from cage 2·(OTf)_8_, before and after workup with Et_2_O. It is evident that the product remains intact.

# 6 Volume calculation

Molovol 1.0.0^[18]^ has been used to calculate the cavity volume and shape of structures **1-2** from crystal structures. The “Probe-occupied volume” (V_occ_) has been obtained via calculation using the “single-probe mode”. The parameters used for the calculation are:

**1**·(BF_4_)_12_:

Probe radius: 1.2 Å

Grid resolution: 0.2 Å

Optimization depth: 4

Element radii: Cu: 2.38 Å, N: 1.66 Å, H: 1.20 Å, C: 1.77 Å.

**1**·(OTf)_12_:

Probe radius: 1.2 Å

Grid resolution: 0.2 Å

Optimization depth: 4

Element radii: Cu: 2.38 Å, N: 1.66 Å, H: 1.20 Å, C: 1.77 Å.

Molovol calculation of the SCXRD structure of **2** using a 3.0 Å diameter probe reveals no isolated cavities, indicating that a continuous pore wider than 3.0 Å runs through the center of the structure. Cage **2** is therefore best described as unenclosed.


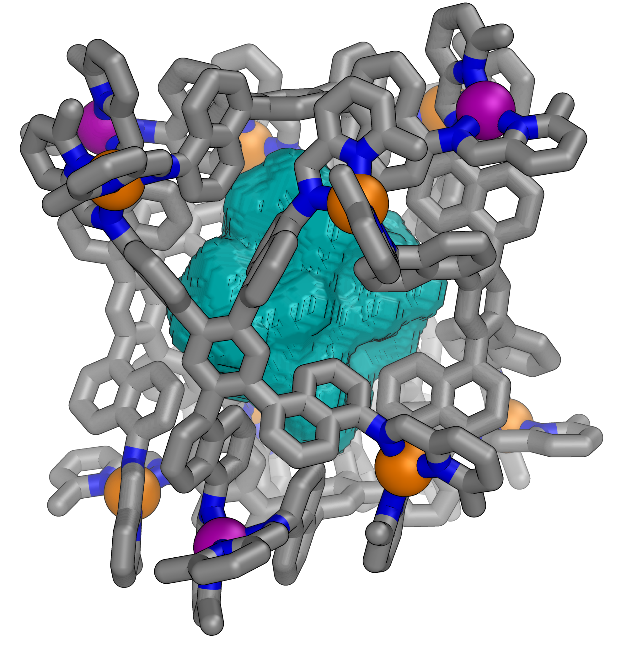


Figure S42. Void space (teal mesh) within the crystal structure of 1·OTf, measuring 486 Å^3^, calculated using MoloVol. ^[18]^

# 7 Self-assembly and reconfiguration attempts with analogous subcomponents

## 7.1 Self-assembly attempt towards analog of cage 1 with 6-fluoro-2-formylpyridine (D)


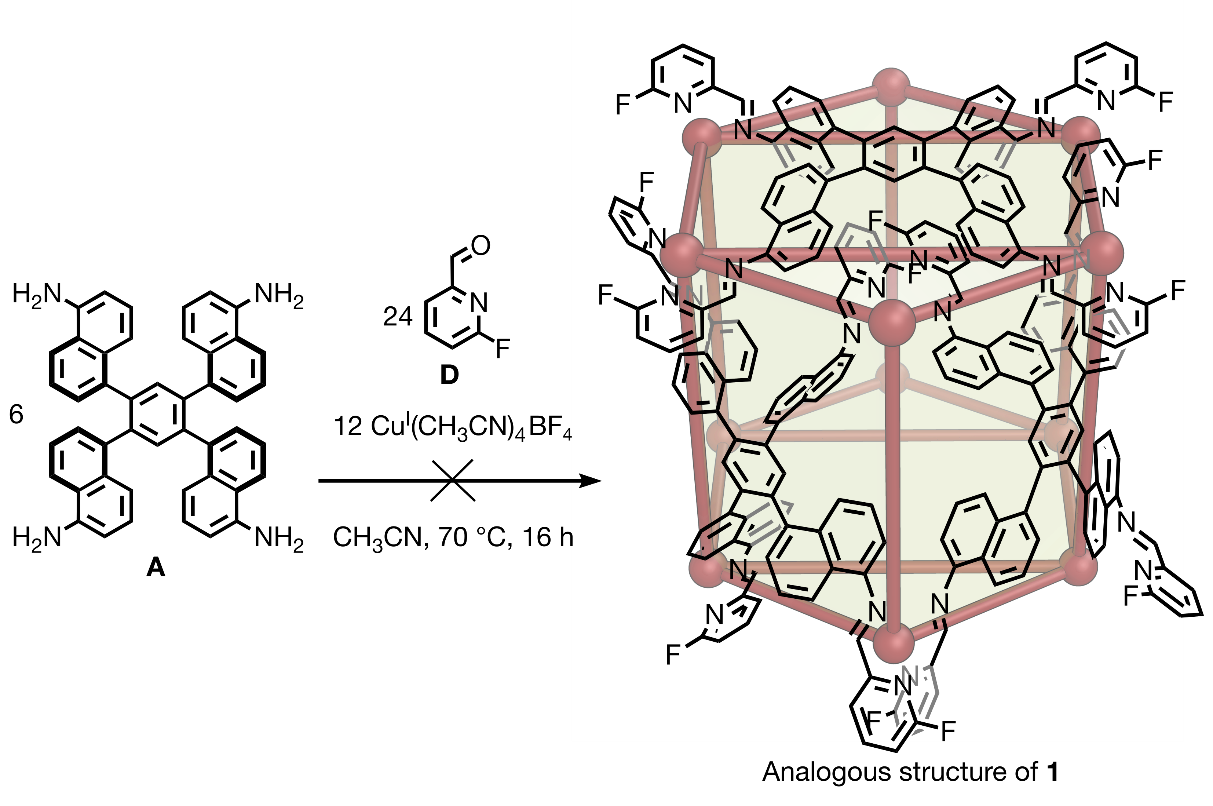


Scheme S4. The self-assembly attempt towards an analogous structure of 1 with 6-fluoro-2-formylpyridine (D).

The attempted preparation and characterization of an analogue of **1** with 6-fluoro-2-formylpyridine (**D**) is summarized as below:

Tetramine subcomponent **A** (4.11 mg, 6.39 µmol, 1.0 equiv.) and Cu(CH_3_CN)_4_BF_4_ (4.02 mg, 12.8 µmol, 2.0 equiv.) were mixed in a Schlenk tube before a solution of 6-methyl-2-formylpyridine (**B**) in freshly distilled acetonitrile (3.52 mg, 28.1 µmol, 4.4 equiv.) was added. The mixture was degassed via three freeze-pump-thaw cycles, before being heated at 70°C for 16 hours. The reaction was cooled to room temperature, concentrated to 0.2 mL under a flow of N_2_, and the product precipitated by addition of diethyl ether (15 mL). The mixture was then centrifuged and the supernatant was discarded. The precipitate was washed with diethyl ether (3 × 15 mL) and dried *in vacuo*, yielding a dark brown solid (9.69 mg).


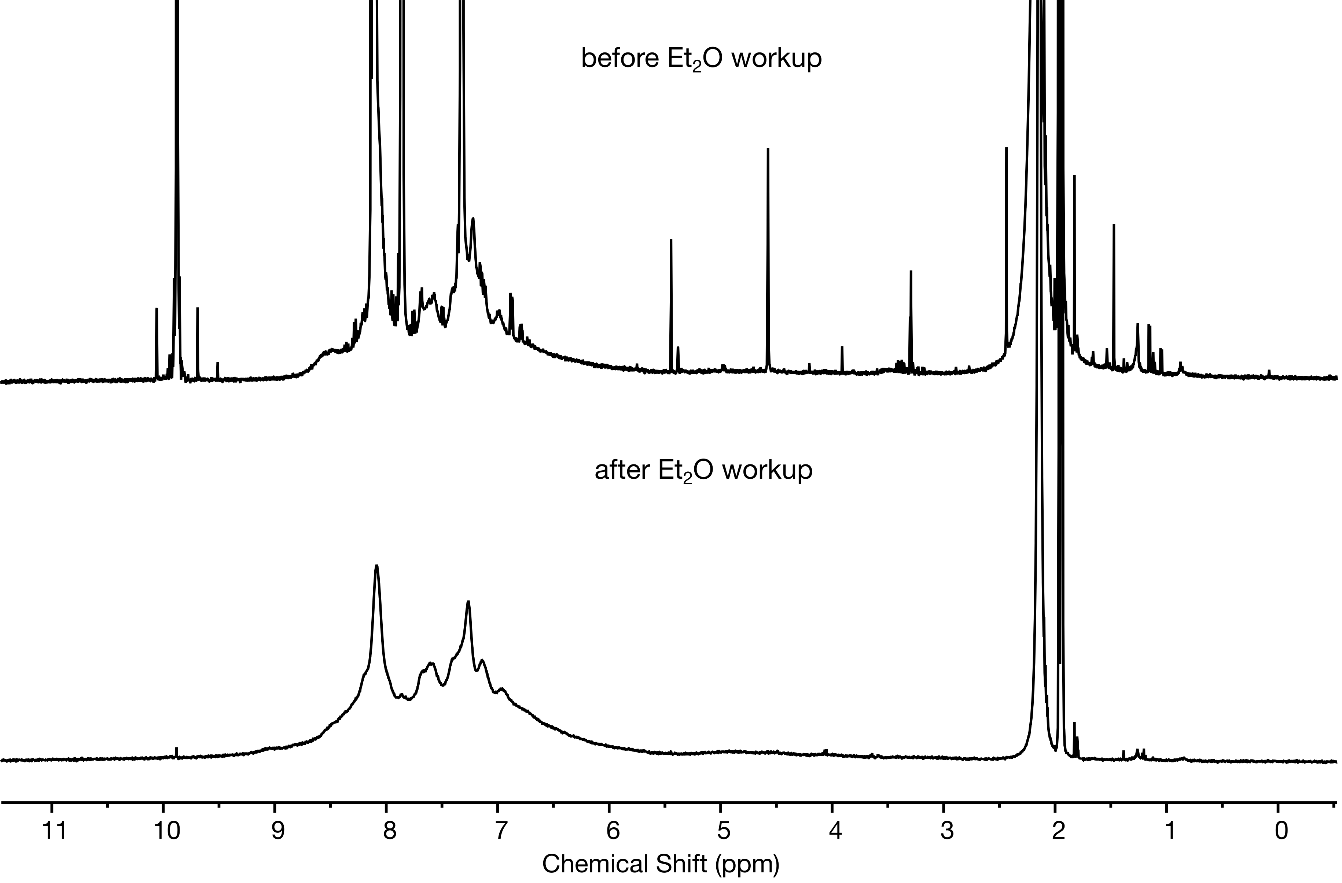


Figure S43. ^1^H NMR Spectrum (500 MHz, CD_3_CN, 298 K) of the reaction mixture of self-assembly attempt towards an analogous structure of 1 with 6-fluoro-2-formylpyridine (D), prior to and after workup with Et_2_O precipitation.


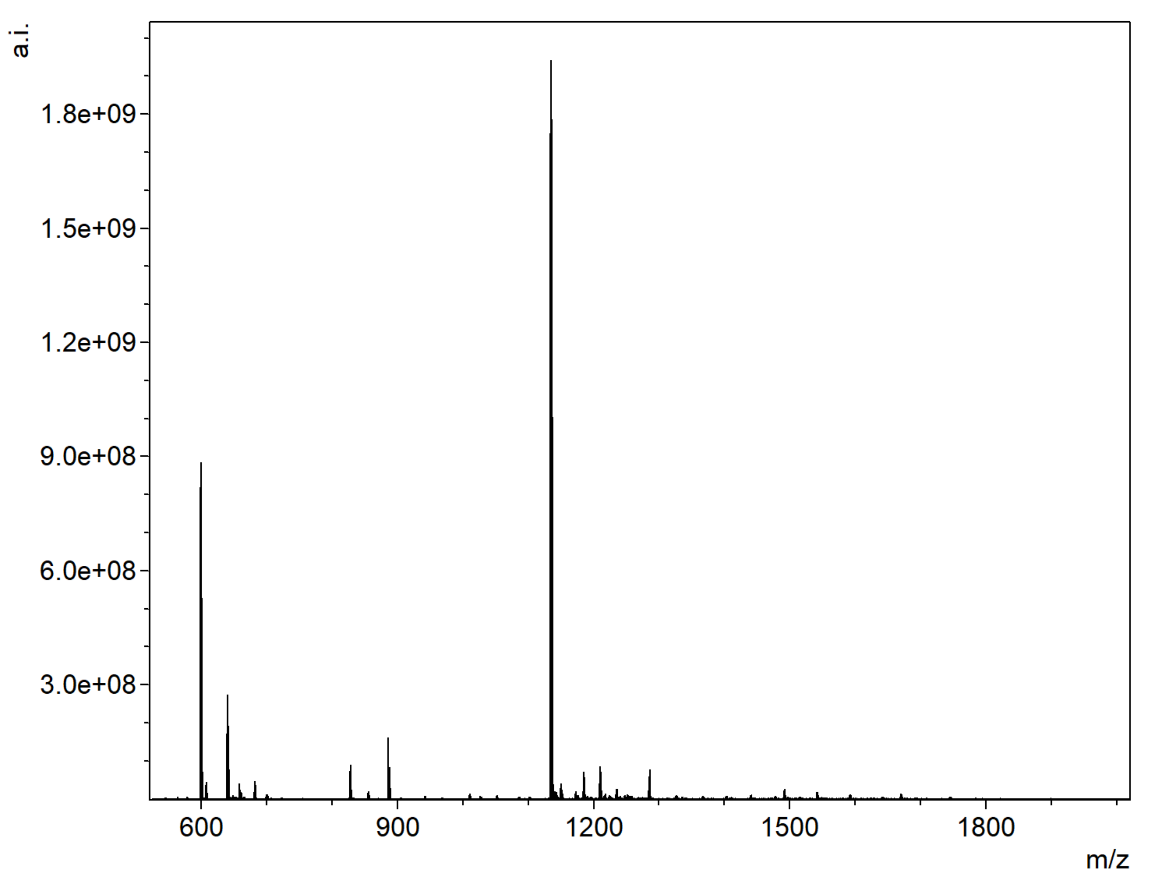


Figure S44. ESI-HRMS Spectrum of the reaction mixture of self-assembly attempt towards an analogous structure of 1 with 6-fluoro-2-formylpyridine. No signals indicating the presence of multiply charged species were observed.

No discrete cage architecture analogous to **1** was identified from the ^1^H NMR and ESI-HRMS data. This result is consistent with the point that the interaction pattern stabilizing **1** is not reproduced in the fluorinated system. The experiment thus supports the importance of the secondary interaction network in the formation of **1**.

## 7.2 Self-assembly attempt towards analog of cage 2 with 3-bromo-2-formylpyridine (E)


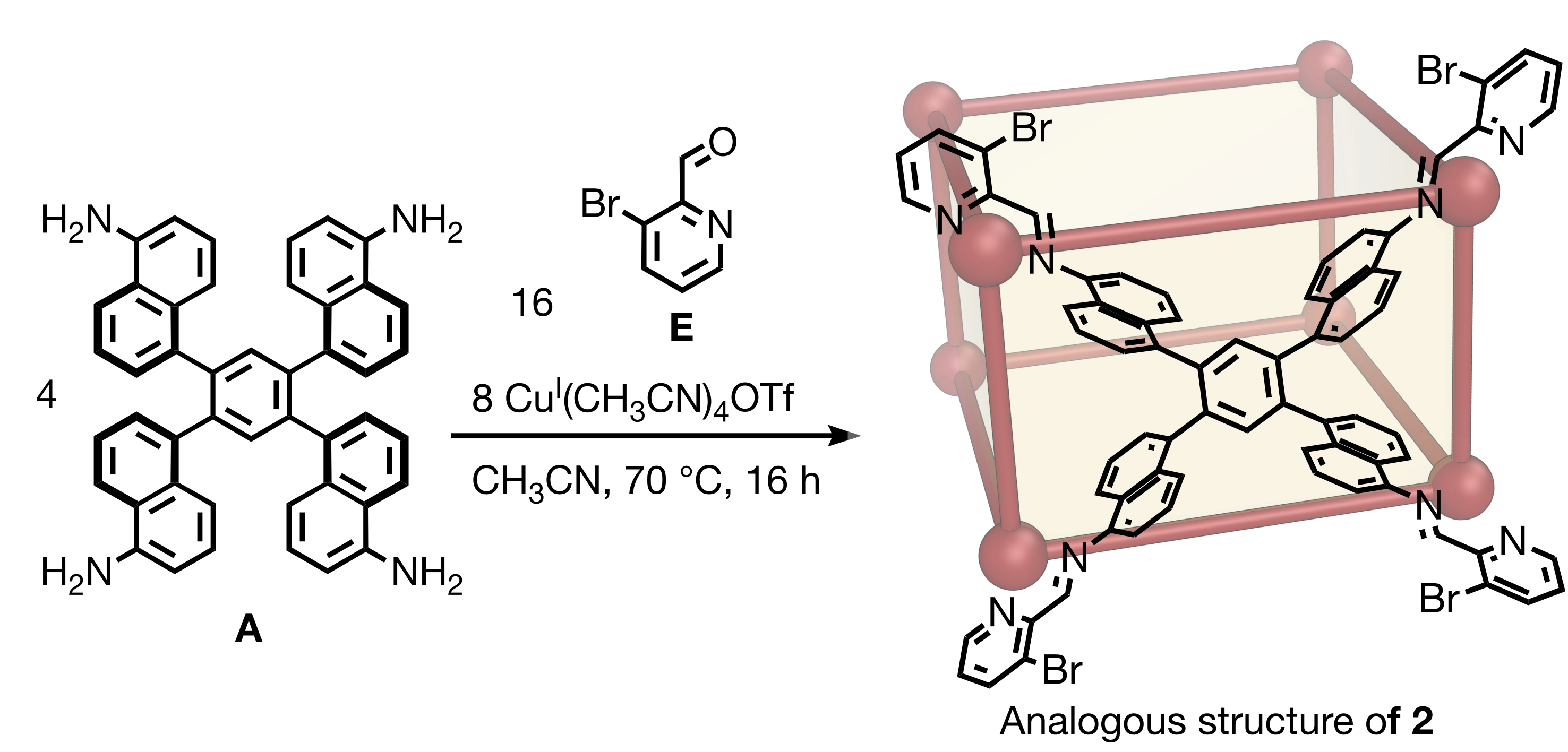


Scheme S5. Self-assembly attempt towards an analog of 2 using 3-bromo-2-formylpyridine (E).

Tetramine subcomponent **A** (4.11 mg, 6.39 µmol, 1.0 equiv.) and Cu(CH_3_CN)_4_OTf (4.82 mg, 12.8 µmol, 2.0 equiv.) were mixed in a Schlenk tube before a solution of 3-methyl-2-formylpyridine (**E**) in freshly distilled CH_3_CN (5.23 mg, 28.1 µmol, 4.4 equiv.) was added. The mixture was degassed via three freeze-pump-thaw cycles, before being heated at 70°C for 16 hours. The reaction was cooled to room temperature, concentrated to 0.2 mL under a flow of N_2_, and precipitated by addition of diethyl ether (15 mL). The mixture was then centrifuged and the supernatant was discarded. The precipitate was washed with diethyl ether (3 × 15 mL) and dried *in vacuo*, yielding a dark brown solid (9.95 mg, 89% assuming a single Cu^I^_8_L_4_·[OTf]_8_ product is generated).


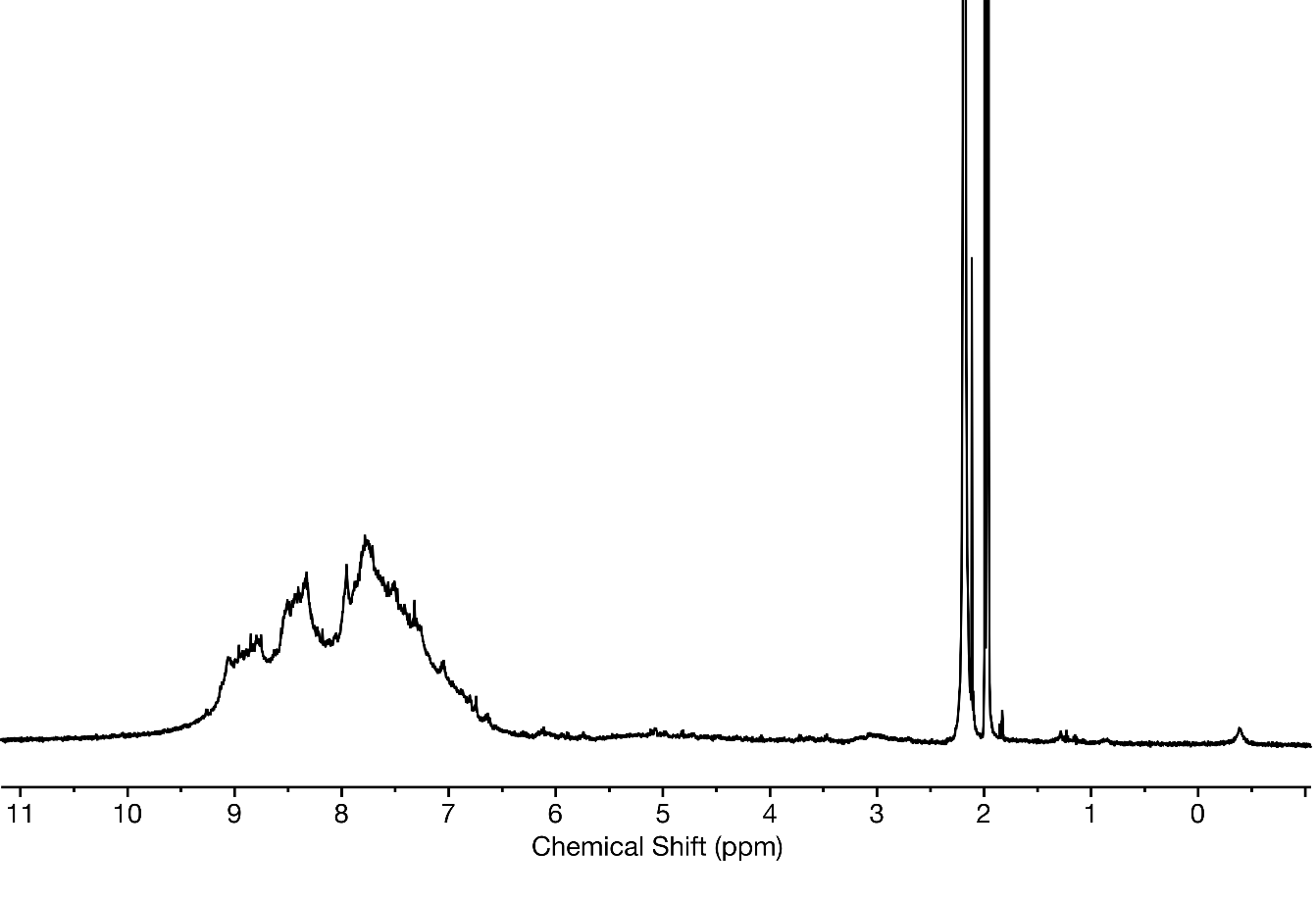


Figure S45. ^1^H NMR Spectrum (500 MHz, CD_3_CN, 298 K) of the reaction mixture obtained from the self-assembly attempt toward an analogue of 2 using 3-bromo-2-formylpyridine (E).


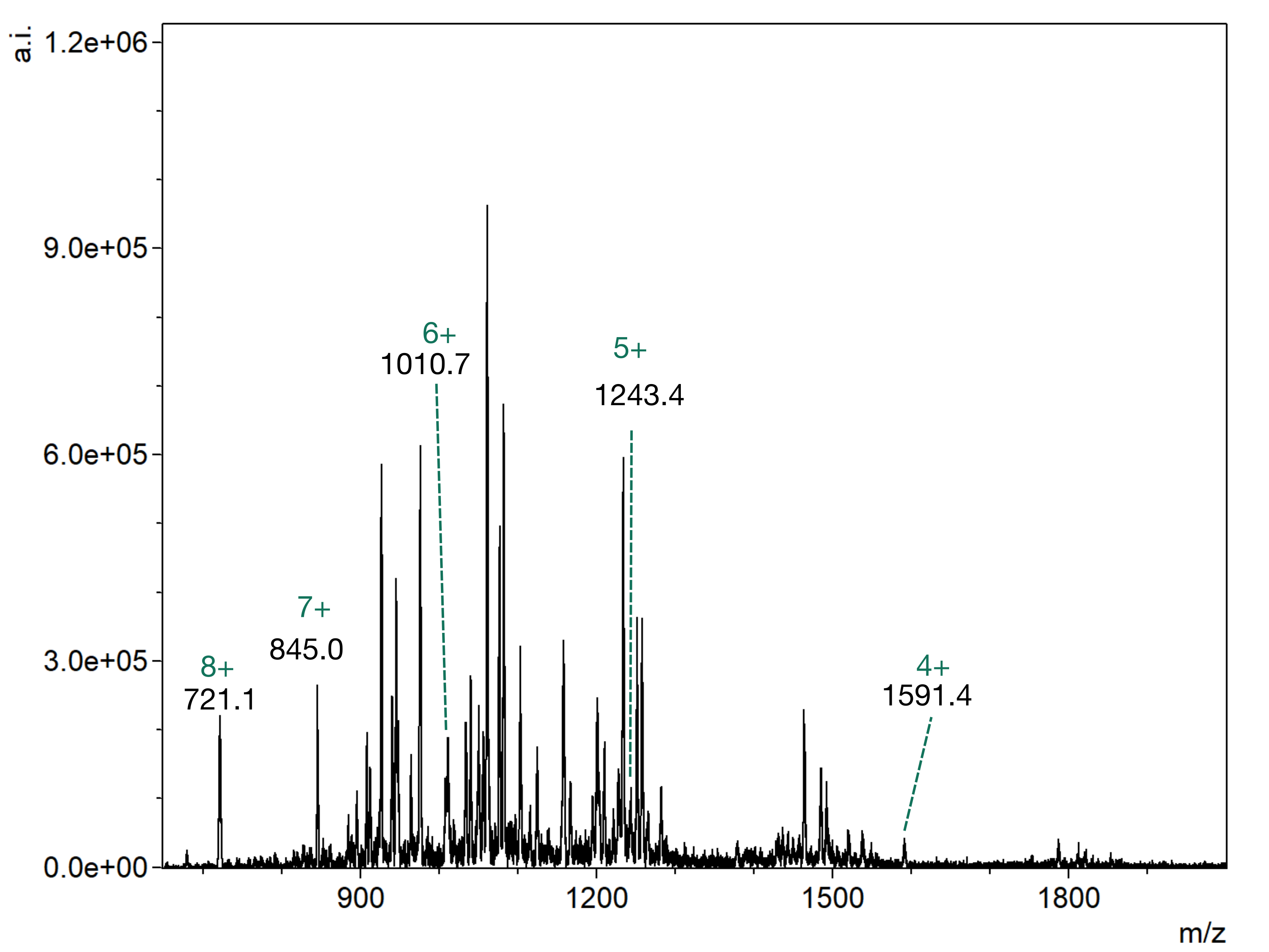


Figure S46. ESI-MS spectrum of the reaction mixture obtained from the self-assembly attempt toward an analogue of 2 using 3-bromo-2-formylpyridine (E). Signals corresponding to the analogue of 2 were identified together with numerous other fragments.


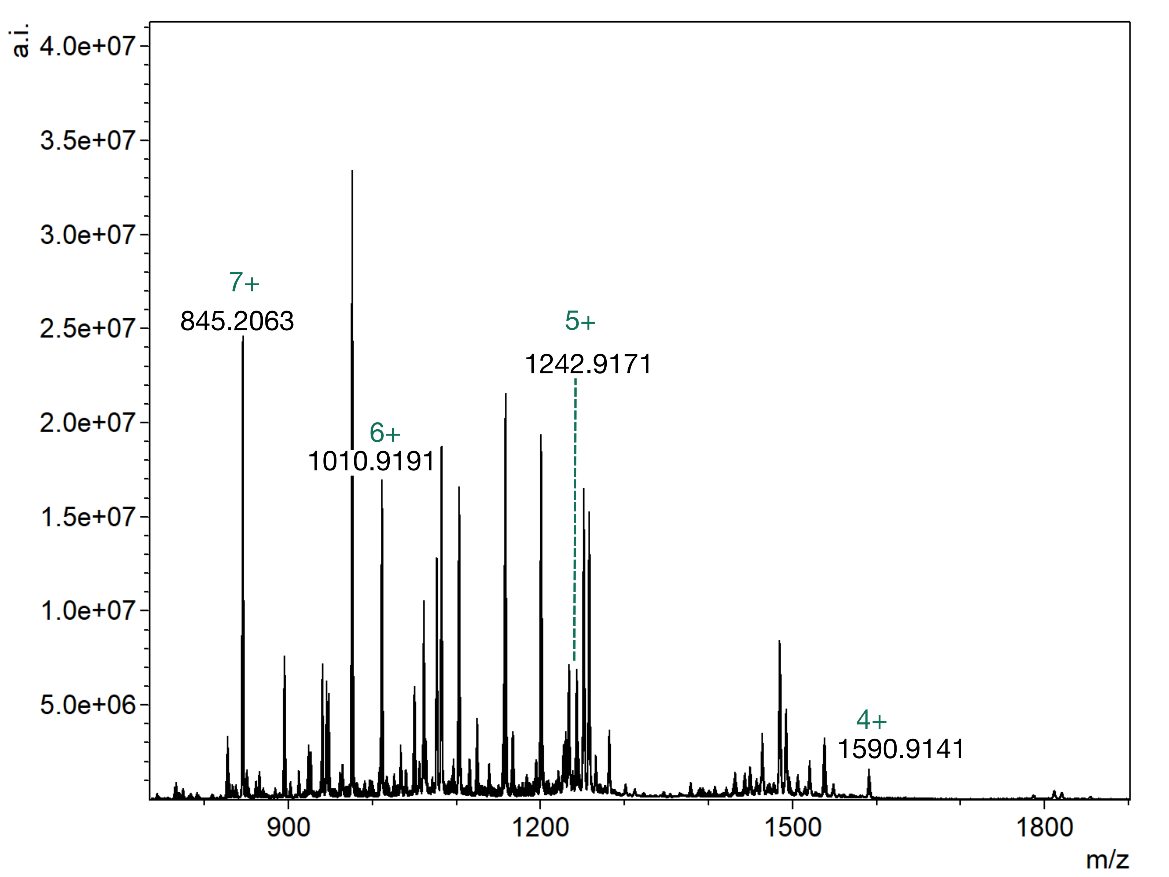


Figure S47. ESI-HRMS spectrum of the reaction mixture obtained from the self-assembly attempt toward an analogue of 2 using 3-bromo-2-formylpyridine (E). Signals corresponding to the analogue of 2 were identified together with numerous other fragments.


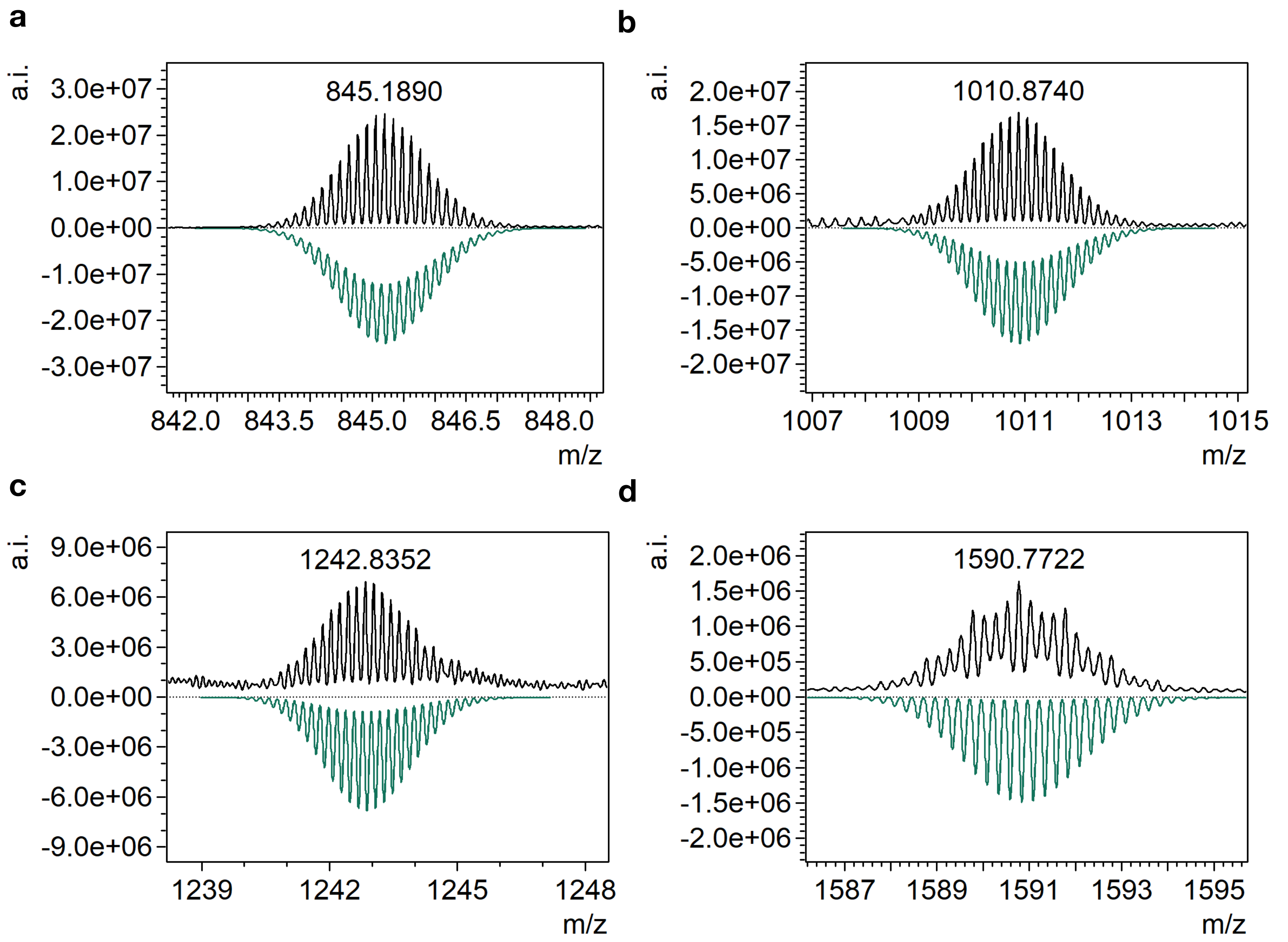


Figure S48. Signals from the high-resolution ESI-mass spectrum for the reaction mixture obtained from the self-assembly attempt toward an analogue of 2 using 3-bromo-2-formylpyridine (E). Experimental (black) and calculated (green) signals for a [Cu_8_L_4_·(OTf)]^7+^ b [Cu_8_L_4_·(OTf)_2_]^6+^ c [Cu_8_L_4_·(OTf)_3_]^5+^ d [Cu_8_L_4_·(OTf)_4_]^4+^.

In contrast to the clean formation of **2** from **C**, the self-assembly with **E** did not give a single discrete product, but instead afforded a mixture containing signals attributable to the Cu^I^_8_L_4_ analogue together with numerous other fragments. The brominated system therefore does not provide a clean differentiated analogue of **2** in terms of mass spectrometry. This point is relevant to the exchange experiment described below, in which **E** serves as an informative but non-innocent mass label.

## 7.3 Reconfiguration from the assembly mixture with 3-bromo-2-formylpyridine (E) to generate analogs of 1


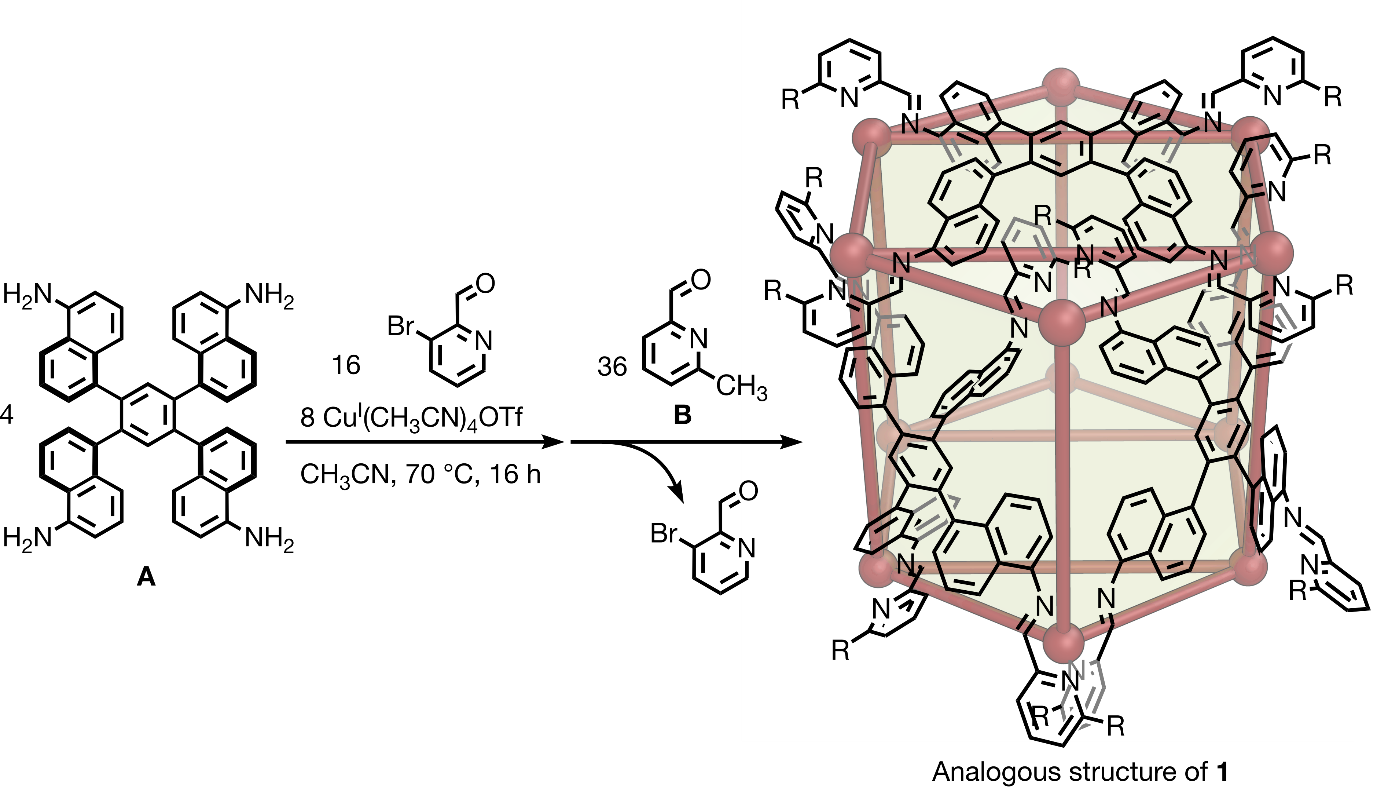


Scheme S6. The structural reconfiguration attempt towards an analogous structure of 1, from the self-assembly with A (4 equiv.), Cu^I^(CH_3_CN)_4_OTf (8 equiv.), and 3-bromo-2-formylpyridine (16 equiv.), by the addition of 6-methyl-2-formylpyridine (36 equiv.).

The self-assembly mixture obtained from Supporting Information Section 7.2 (10.10 mg, 1.45 μmol, assuming a single Cu^I^_8_L_4_·[OTf]_8_ product) was dissolved in degassed acetonitrile (500 μL). 6-methyl-2-formylpyridine (52.5 mmol·L^-1^ in CH_3_CN, 1000 μL, 36.0 equiv.) was added and the mixture was degassed via three freeze-pump-thaw cycles before heating at 70°C for 70 h and monitored by ESI-MS.


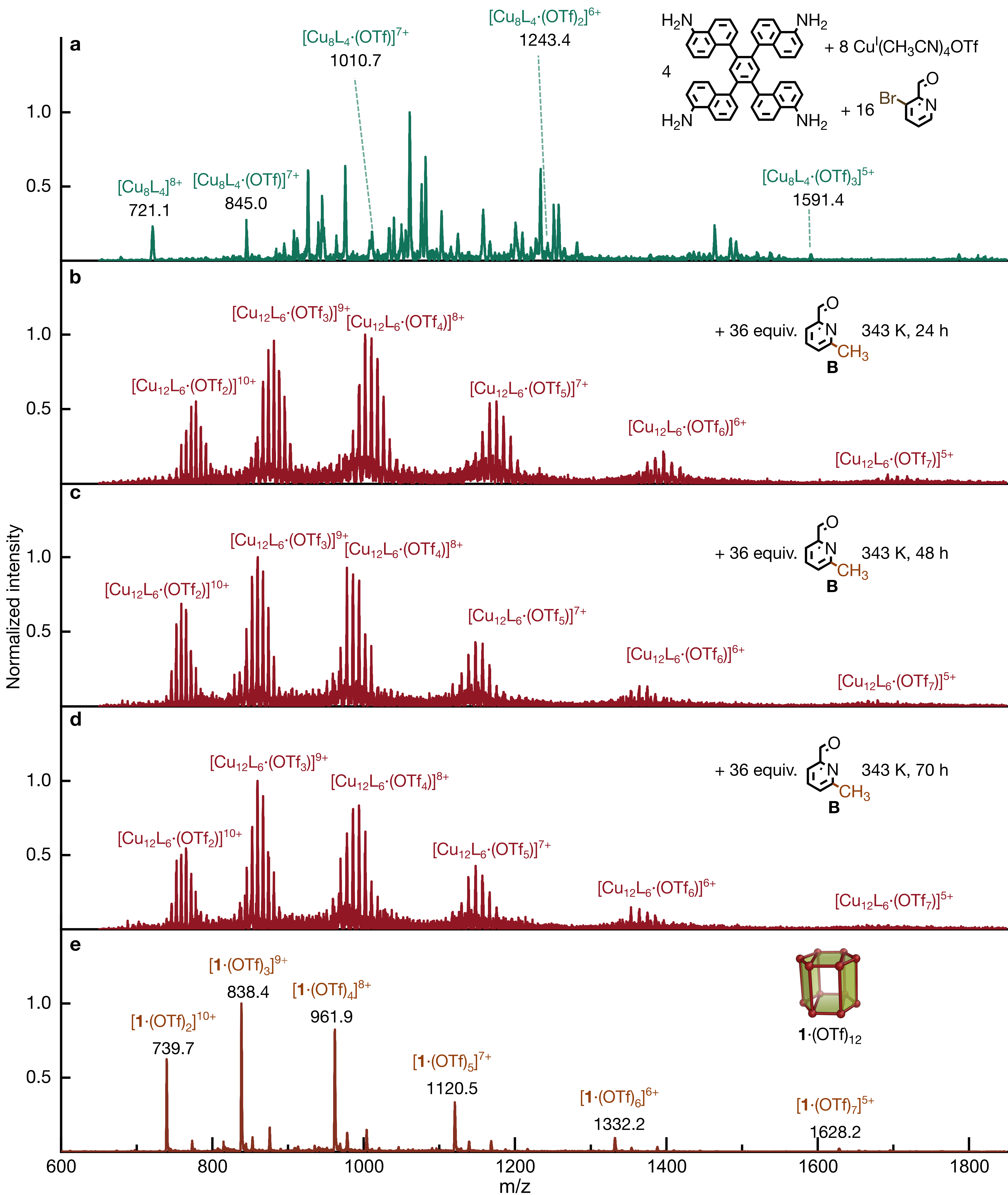


Figure S49. Stacked ESI-MS spectra tracking the reconfiguration of the assembly mixture obtained from A, Cu^I^(CH_3_CN)_4_OTf, and 3-bromo-2-formylpyridine (E) after addition of 6-methyl-2-formylpyridine (B) and heating. a, ESI-MS spectrum of the starting mixture. b-d, ESI-MS spectra of the same sample after addition of B and heating at 343 K for b 24 h, c 48 h, and d 70 h. e, ESI-MS spectrum of 1·(OTf)_12_ for reference.


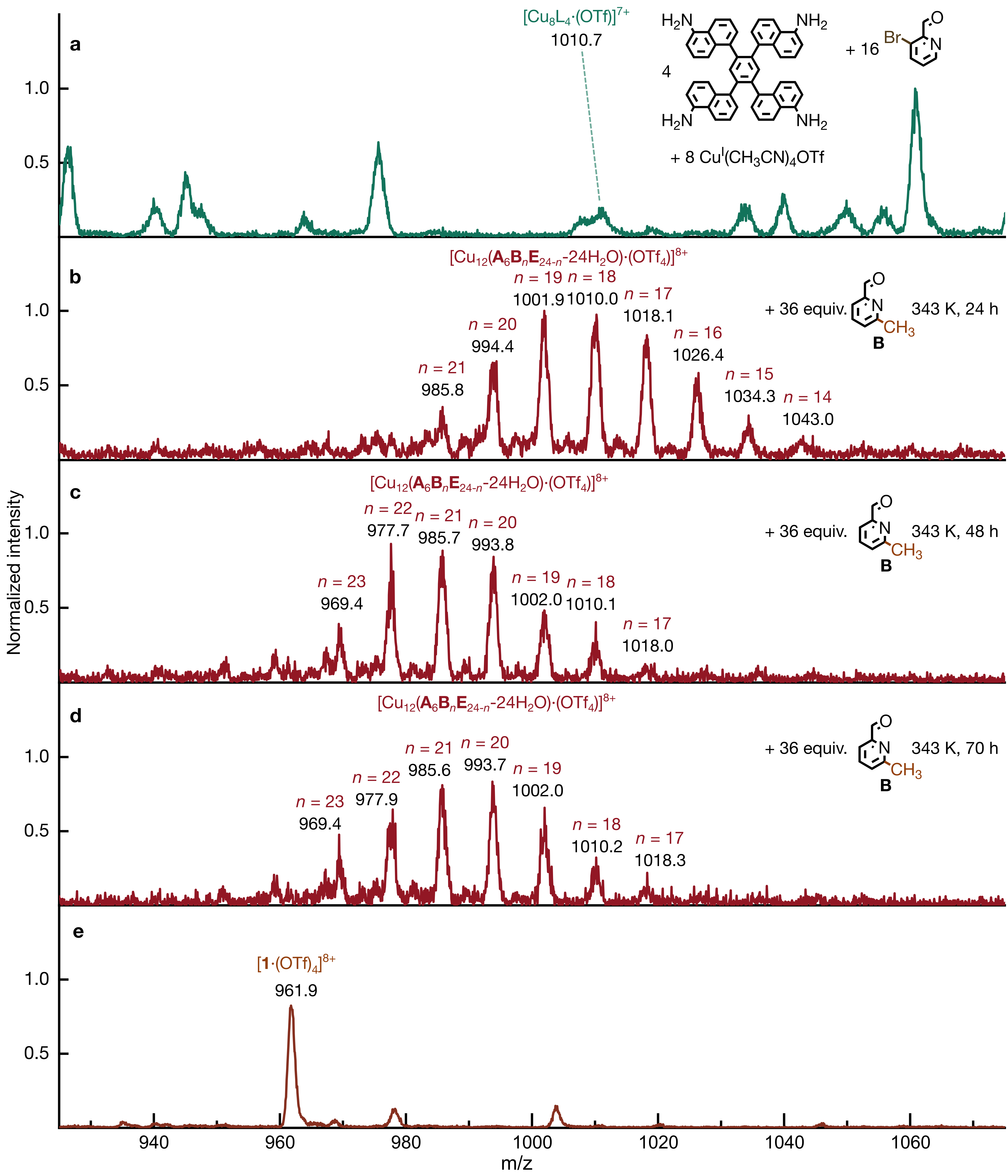


Figure S50. Stacked partial ESI-MS spectra tracking the same reconfiguration shown in Figure S49, highlighting the number of imine residues displaced per cage. a, ESI-MS spectrum of the starting mixture. b-d, ESI-MS spectra of the same sample after addition of B and heating at 343 K for b 24 h, c 48 h, and d 70 h. e, ESI-MS spectrum of 1·(OTf)_12_ for reference.


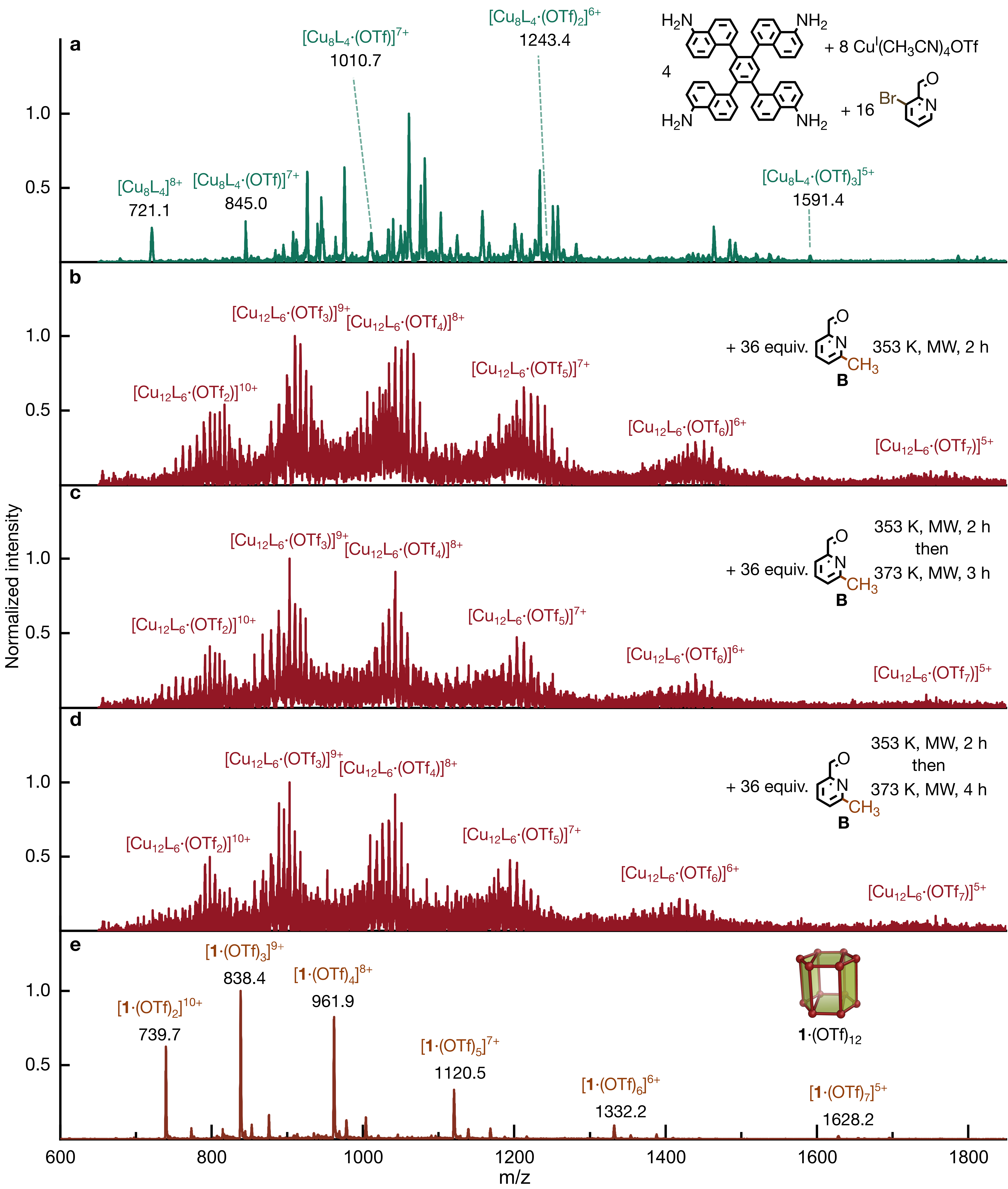


Figure S51. Stacked ESI-MS spectra tracking the reconfiguration of the assembly mixture obtained from A, Cu^I^(CH_3_CN)_4_OTf, and 3-bromo-2-formylpyridine (E) after addition of 6-methyl-2-formylpyridine (B) under microwave-assisted conditions. a, ESI-MS spectrum of the starting mixture. b-d, ESI-MS spectra of the same sample after addition of B and microwave heating at b 353 K for 2 h, then at c 373 K for 3 h d and 373 K for 1 h. e, ESI-MS spectrum of 1·(OTf)_12_ for reference.


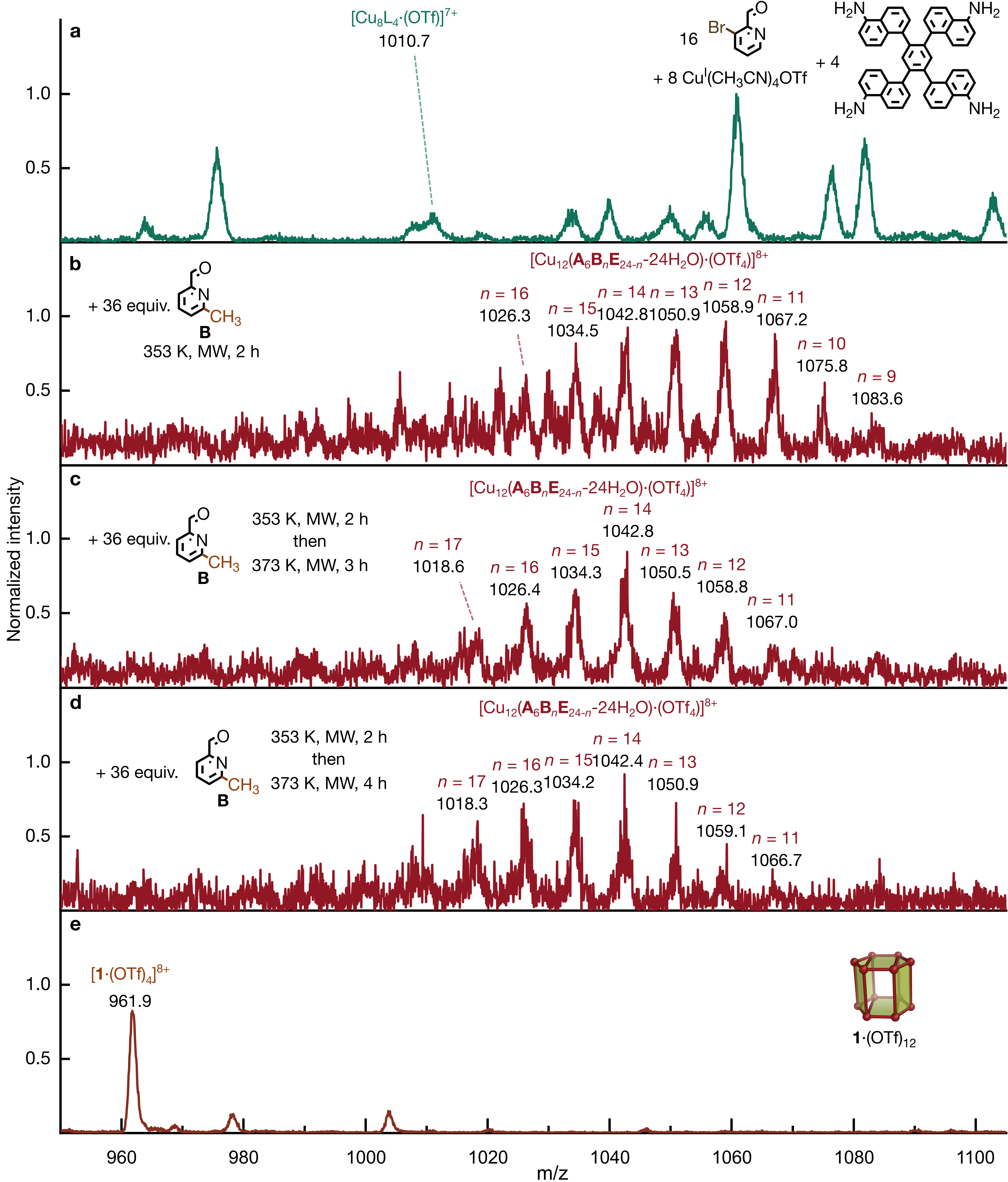


Figure S52. Stacked partial ESI-MS spectra tracking the same microwave-assisted reconfiguration shown in Figure S51, highlighting the number of imine residues displaced per cage. a ESI-MS spectrum of the starting mixture. b-d, ESI-MS spectrum of the same sample after addition of 36 equiv. of 6-methyl-2-formylpyridine (B) and microwave-assisted heating at b 353 K for 2 h, then at c 373 K for 3 h d and 373 K for 1 h. e ESI-MS spectrum of a sample of 1·(OTf)_12_ for reference.

Upon addition of **B**, the brominated assembly mixture reconfigured to a family of [Cu^I^_12_L_6_]^12+^ species with varying numbers of displaced aldehyde residues. The observed displacement numbers were higher, and the reconfiguration was significantly faster, than in the corresponding methyl-substituted system detailed in Section 5. These observations indicate that, although access to a **1**-type framework requires relief of the destabilizing steric hindrances associated with 3-substituted iminopyridyl residues, the brominated system continues to undergo further exchange after framework formation. Importantly, no species with fewer than eight displaced residues were observed.

# References

[1] I. de Toledo, T. A. Grigolo, J. M. Bennett, J. M. Elkins, R. A. Pilli, *J. Org. Chem.* **2019**, *84*, 14187–14201.

[2] D. R. Allan, H. Nowell, S. A. Barnett, M. R. Warren, A. Wilcox, J. Christensen, L. K. Saunders, A. Peach, M. T. Hooper, L. Zaja, S. Patel, L. Cahill, R. Marshall, S. Trimnell, A. J. Foster, T. Bates, S. Lay, M. A. Williams, P. V. Hathaway, G. Winter, M. Gerstel, R. W. Wooley, *Crystals* **2017**, *7*.

[3] a P. Evans, *Acta Crystallogr., Sect. D:Biol. Crystallogr.* **2006**, *62*, 72–82; b G. Winter, *J. Appl. Crystallogr.* **2010**, *43*, 186–190; c G. Winter, D. G. Waterman, J. M. Parkhurst, A. S. Brewster, R. J. Gildea, M. Gerstel, L. Fuentes-Montero, M. Vollmar, T. Michels-Clark, I. D. Young, N. K. Sauter, G. Evans, *Acta Crystallogr., Sect. D:Struct. Biol.* **2018**, *74*, 85–97.

[4] J. Beilsten-Edmands, G. Winter, R. Gildea, J. Parkhurst, D. Waterman, G. Evans, *Acta Crystallogr., Sect. D* **2020**, *76*, 385–399 , ISSN = 2059–7983 , DOI = doi 2010.1107/S2059798320003198.

[5] G. Sheldrick, *Acta Crystallogr., Sect. A* **2015**, *71*, 3–8.

[6] G. Sheldrick, *Acta Crystallogr. C* **2015**, *71*, 3–8.

[7] a O. S. Smart, T. O. Womack, A. Sharff, C. Flensburg, P. Keller, W. Paciorek, C. Vonrhein, G. Bricogne, Grade, version 1.2.20, Global Phasing Ltd., Cambridge, United Kingdom, **2011**; b O. S. Smart, A. Sharff, J. Holstein, T. O. Womack, C. Flensburg, P. Keller, W. Paciorek, C. Vonrhein, G. Bricogne, Grade2, version 1.7.1, Global Phasing Ltd., Cambridge, United Kingdom, **2021**.

[8] a P. van der Sluis, A. L. Spek, *Acta Crystallogr., Sect. A* **1990**, *46*, 194–201; b A. Spek, *Acta Crystallogr. C* **2015**, *71*, 9–18.

[9] A. L. Spek, *J. Appl. Crystallogr.* **2003**, *36*, 7–13.

[10] C. F. Macrae, I. Sovago, S. J. Cottrell, P. T. A. Galek, P. McCabe, E. Pidcock, M. Platings, G. P. Shields, J. S. Stevens, M. Towler, P. A. Wood, *J. Appl. Crystallogr.* **2020**, *53*, 226–235.

[11] D. B. Ninković, J. P. Blagojević Filipović, M. B. Hall, E. N. Brothers, S. D. Zarić, *ACS Cent. Sci.* **2020**, *6*, 420–425.

[12] a T. Steiner, *Angew. Chem. Int. Ed.* **2002**, *41*, 48–76; b G. A. Jeffrey, *An Introduction to Hydrogen Bonding*, UK ed. ed., Oxford University Press, U.S.A., New York, **1997**; c M. Kumar, P. V. Balaji, *J. Mol. Model.* **2014**, *20*, 2136.

[13] G. Landrum, *RDKit: Open-source Cheminformatics*. https://www.rdkit.org.

[14] C. R. Harris, K. J. Millman, S. J. van der Walt, R. Gommers, P. Virtanen, D. Cournapeau, E. Wieser, J. Taylor, S. Berg, N. J. Smith, R. Kern, M. Picus, S. Hoyer, M. H. van Kerkwijk, M. Brett, A. Haldane, J. F. del Río, M. Wiebe, P. Peterson, P. Gérard-Marchant, K. Sheppard, T. Reddy, W. Weckesser, H. Abbasi, C. Gohlke, T. E. Oliphant, *Nature* **2020**, *585*, 357–362.

[15] Y. V. Zefirov, P. M. Zorky, *Russ. Chem. Rev.* **1995**, *64*, 415.

[16] S. Alvarez, *Dalton Trans.* **2013**, *42*, 8617–8636.

[17] M. R. Willcott, *J. Am. Chem. Soc.* **2009**, *131*, 13180–13180.

[18] J. B. Maglic, R. Lavendomme, *J. Appl. Crystallogr.* **2022**, *55*, 1033–1044.
